# Process Design and Greenhouse Gas Analysis of an Electrified Mixed Plastic Waste-to-Olefins Value Chain

**DOI:** 10.1021/acs.iecr.6c01225

**Published:** 2026-08-16

**Authors:** Amvrosios G. Georgiadis, Vasileia-Loukia Yfanti, Ismaël Amghizar, David J. Brown, Azd Zayoud, Guy B. Marin, Kevin Van Geem, Evangelos Delikonstantis, Stavros-Alexandros Theofanidis

**Affiliations:** † AristEng S.à r.l., 20, Avenue Pasteur L-2310 Luxembourg City, Luxembourg; ‡ AVGI bv, Technologiepark-Zwijnaarde 122 9052 Ghent, Belgium; § Laboratory for Chemical Technology, Ghent University 9052 Ghent, Belgium; ∥ Pryme NV 3065 Rotterdam, The Netherlands

## Abstract

This work presents the conceptual process design and life-cycle Greenhouse Gas (GHG) analysis for converting mixed plastic waste (MPW) into polymer-grade olefins (≥99.9 wt % ethylene and propylene) via sequential pyrolysis, e-cracking, compression, and separation. The integrated plant-wide process modeling was performed within a Python-based framework as an alternative to conventional commercial simulators. Eight scenarios were assessed by combining centralised and decentralised configurations, combined heat and power (CHP)-powered and fully electrified operation, and grid versus renewable electricity supply. Under fully electrified conditions, the carbon footprint strongly depends on the electricity source. When onshore wind-harvested electricity is assumed, GHG emissions decrease to ∼0.08 kg CO_2‑eq_ per kg of high-value chemicals (HVCs), placing the process within the EU’s 2040 climate objective of a 90% reduction relative to the sector’s conventional benchmark (i.e., naphtha steam cracking, ∼1 kg CO_2‑eq_ per kg of HVC). Sensitivity analysis showed that transport-related emissions are a significant contributor in the fully electrified scenario. In particular, maintaining the targeted 90% GHG reduction relative to the conventional benchmark is only feasible for MPW transport distances of ∼ 160–180 km.

## Graphical abstract

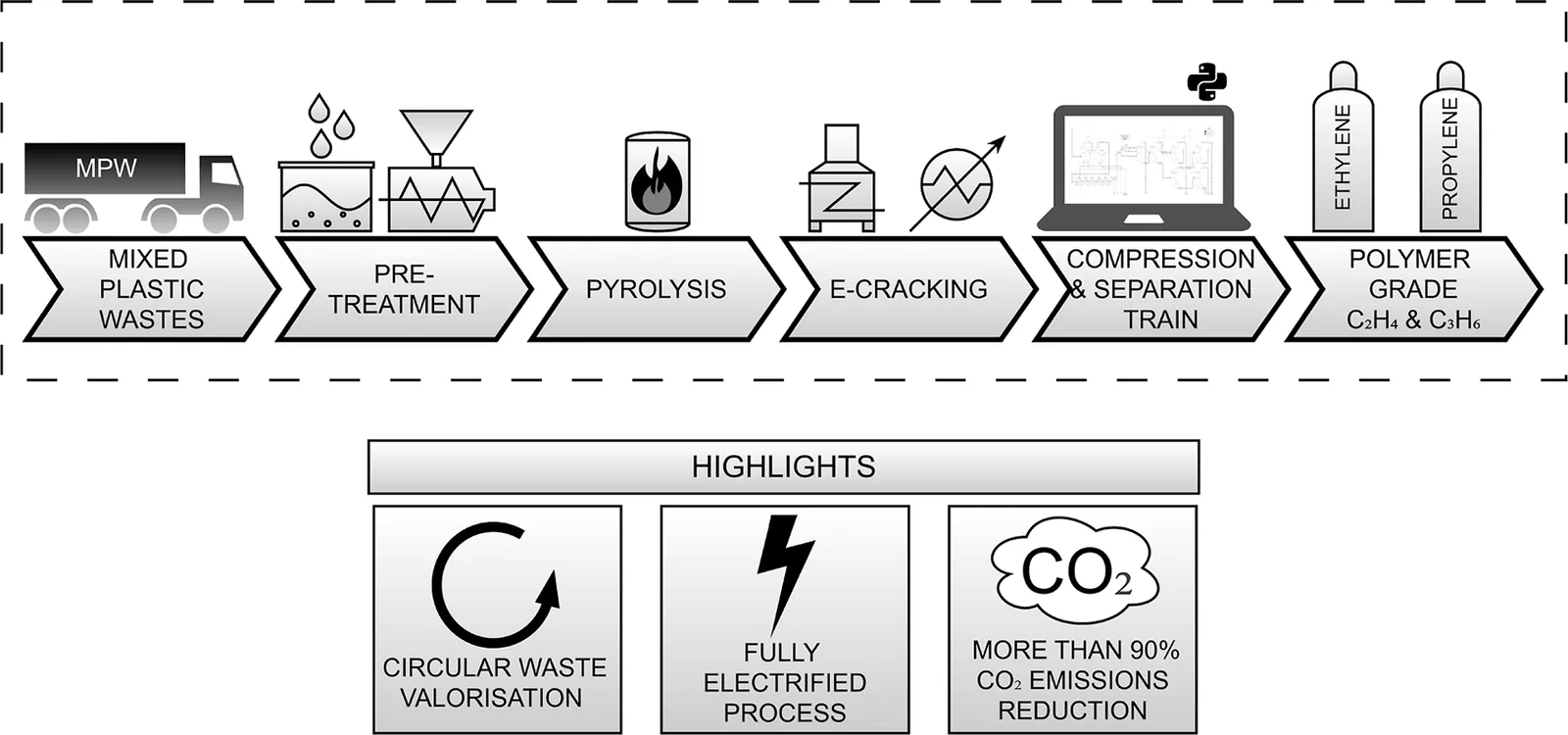

## 1 Introduction

Plastics have become indispensable in modern society due to their versatility, durability, and cost-effectiveness. Global production exceeded 400 million tonnes in 2022 ^1^ and reached approximately 414 million tonnes in 2023, driven largely by demand in packaging, automotive, and construction sectors.^2^ Despite this growth, plastic waste management remains a major global challenge: only about 9% of plastic waste is recycled, while 19% is incinerated, nearly 50% is landfilled, and around 22% is mismanaged or leaked into the environment.^3^ Mixed plastic waste (MPW), which contains heterogeneous polymers and contaminants, is particularly difficult to recycle mechanically because such processes require well-sorted, high-quality feedstocks.^4,5^ Consequently, large fractions of postconsumer plastics remain unsuitable for conventional (mechanical) recycling, posing persistent barriers to achieving plastic circularity.^6^

Among chemical routes, thermal (i.e., noncatalytic) pyrolysis has attracted significant attention for its ability to thermally degrade a wide range of polymers into gas, oil and wax fractions without the need for precise sorting or pretreatment.^7,8^ In case of plastic waste pyrolysis, the resulting pyrolysis oil closely resembles fossil-derived oil and consists of hydrocarbons (e.g., paraffins, olefins, naphthenes, and aromatics).^9^ The pyrolysis gas mainly contains H_2_, CH_4_, C_2_–C_4_ hydrocarbons, CO, and CO_2_.^10^ Both pyrolysis oil and gas be converted into high-value chemicals (HVCs), through a sequence of downstream processing steps. These HVCs include ethylene, propylene, benzene, hydrogen, and butadiene, which are essential feedstocks for the chemical industry (e.g., polymer production). Nevertheless, the yield, composition, and quality of pyrolysis oil can vary, depending on feedstock type, additives, and applied conditions (i.e., temperature, catalyst use etc.).^6,11^ Recent reviews have highlighted both the opportunities and challenges associated with plastic waste pyrolysis, including product variability, process integration, and downstream upgrading requirements for circular plastics production.^12,13^

Realizing the environmental benefits of plastic waste pyrolysis remains challenging because both the pyrolysis process and the downstream upgrading/separation steps are highly energy-intensive and still depend heavily on fossil-derived utilities and fuels.^6,14^ In particular, steam cracking is among the most carbon-intensive processes in the petrochemical industry and is widely used for the conversion of fossil- and pyrolysis oil-derived saturated hydrocarbons into olefins. Conventional steam crackers are optimized for fossil-based feedstocks, operate at high temperatures (750–850 °C) and are predominantly fired with methane-rich off-gases generated during the cracking process.^15–17^ They account for ∼260 million tonnes of CO_2_ emissions globally each year.^15,18^ Incorporating plastic waste pyrolysis oils introduces additional operational challenges due to elevated levels of nitrogen, oxygen, halogens, and metals that may lead to fouling and corrosion.^19^

Electrification of both plastic waste pyrolysis and the subsequent steam cracking step, offers a promising pathway for reducing GHG emissions when coupled with low-carbon electricity sources (e.g., wind-harvested electricity).^15,17,20^ Recent studies have shown that fully electrified steam cracker furnaces, using pyrolysis oil as a feedstock and powered by renewable electricity could reduce direct GHG emissions (Scope 1 emissions) by up to 90% compared to conventional fossil-fuel-fired furnaces.^15,21,22^ Pilot-scale initiatives by industrial consortia (e.g., Cracker of the Future,^23^ e-Furnace with direct resistive heating by BASF, SABIC and Linde,^24,25^ patented electrified steam cracker by Haldor Topsoe^26^ etc.) further highlight growing industrial interest in electrified cracking technologies.^27–29^ However, the large-scale implementation of electrified pyrolysis and steam cracking systems remains limited, and comprehensive system-level assessments considering the carbon intensity of electricity, feedstock variability, process integration, and upstream–downstream interactions are still lacking.^30,31^ Recent reviews^15^ further indicate that microwave-assisted pyrolysis (MAP) currently represents one of the most mature electrified plastic waste pyrolysis technologies, with reported TRLs approaching ∼7, whereas nonthermal plasma-assisted pyrolysis remains at lower maturity levels (TRL ∼ 3–4). Despite promising laboratory- and pilot-scale performance, significant scalability, energy efficiency, and process integration challenges still hinder widespread industrial deployment of electrified chemical recycling technologies.^15^

Along these lines, Gu et al.^20^ performed a techno-economic and life-cycle assessment, comparing conventionally fire-heated steam cracking with resistively heated steam cracking, using fossil-derived naphtha as feedstock. The boundary limits include steam cracking as foreground process and all the background processes (i.e., feedstock, utilities etc.). They reported CO_2_ emissions as low as 2.84 kg CO_2‑eq_·kg C_2_H_4_^–1^ for renewable-electricity-powered electrified steam cracking, compared with 3.21–3.73 kg CO_2‑eq_·kg C_2_H_4_^–1^ for conventional naphtha steam cracking, corresponding to GHG emission reductions of up to ∼24% depending on the electricity supply pathway.

Additional studies^32^ have focused on process simulation and environmental assessment of plastic waste conversion pathways, such as pyrolysis and hydrocracking of high-density polyethylene (HDPE). Specifically, Azam et al.^32^ used Aspen Plus to simulate these pathways for the production of hydrocarbon mixtures including pyrolysis oils, gaseous fuels, and gasoline-range hydrocarbons. Distributed conversion and supply chain configurations have also been explored for thermochemical processing systems operating under decentralised configurations, where smaller processing facilities located closer to feedstock sources produce intermediate oils that are subsequently transported to centralized upgrading or distribution facilities.^33^ However, most previous studies focus on individual process units or specific subsystems, limiting holistic evaluation of the complete MPW-to-olefin value chain and the interactions between pyrolysis, downstream upgrading, utility integration, electrification strategies, and overall life-cycle environmental performance. In particular, few studies combine experimentally derived MPW pyrolysis products, detailed downstream e-cracking and cryogenic separation modeling, utility integration, electrification scenarios, and life-cycle GHG assessment within a unified framework.^20,30,34^ Moreover, decentralised process layouts and the influence of varying electrification strategies on utility demand and process integration remain largely unexplored.^15,19^

To address these gaps, this study evaluates an integrated and electrified pathway for converting MPW into olefins (i.e., polymer grade ethylene and propylene) through pyrolysis, steam cracking, and downstream separation. The system encompasses the full conversion chain, from feedstock preprocessing to final product recovery and utility generation. It is evaluated across eight scenarios that combine centralized and decentralised layouts, CHP-powered and fully electrified configurations, and fossil-based and renewable electricity supplies. Particular emphasis is placed on the compression and separation train (CST), where process integration, utility demand, and electrification strategy critically influence overall system performance.

This study further presents, to the authors’ knowledge, one of the first integrated system-level assessments of an electrified MPW-to-olefins process using a fully custom, Python-based simulation framework developed independently of commercial software. The framework enables rigorous mass and energy balance calculations for the CST and supports complete process integration, including automated heat integration and scenario-driven evaluation across a broad configuration space. By coupling this modeling platform with a life-cycle GHG assessment, the study identifies viable decarbonisation pathways. It provides actionable insights for industrial process design and policy development in the context of circular feedstocks and electrified chemical infrastructure.

## 2 Approach & Methodology

### 2.1 Process Description

The conversion of mixed plastic waste into polymer-grade olefins is evaluated through a plantwide process design and projected GHG emissions analysis (i.e., CO_2_ emissions). The process model is developed on the basis of an MPW feedstock equal to 37.5 ktonnes per annum, and all reported flows and energy duties are consistently normalised to one tonne of MPW. Figure 1 presents the aggregated block scheme of the process, highlighting the main transformation steps from plastic waste to olefins.

**Figure 1:**
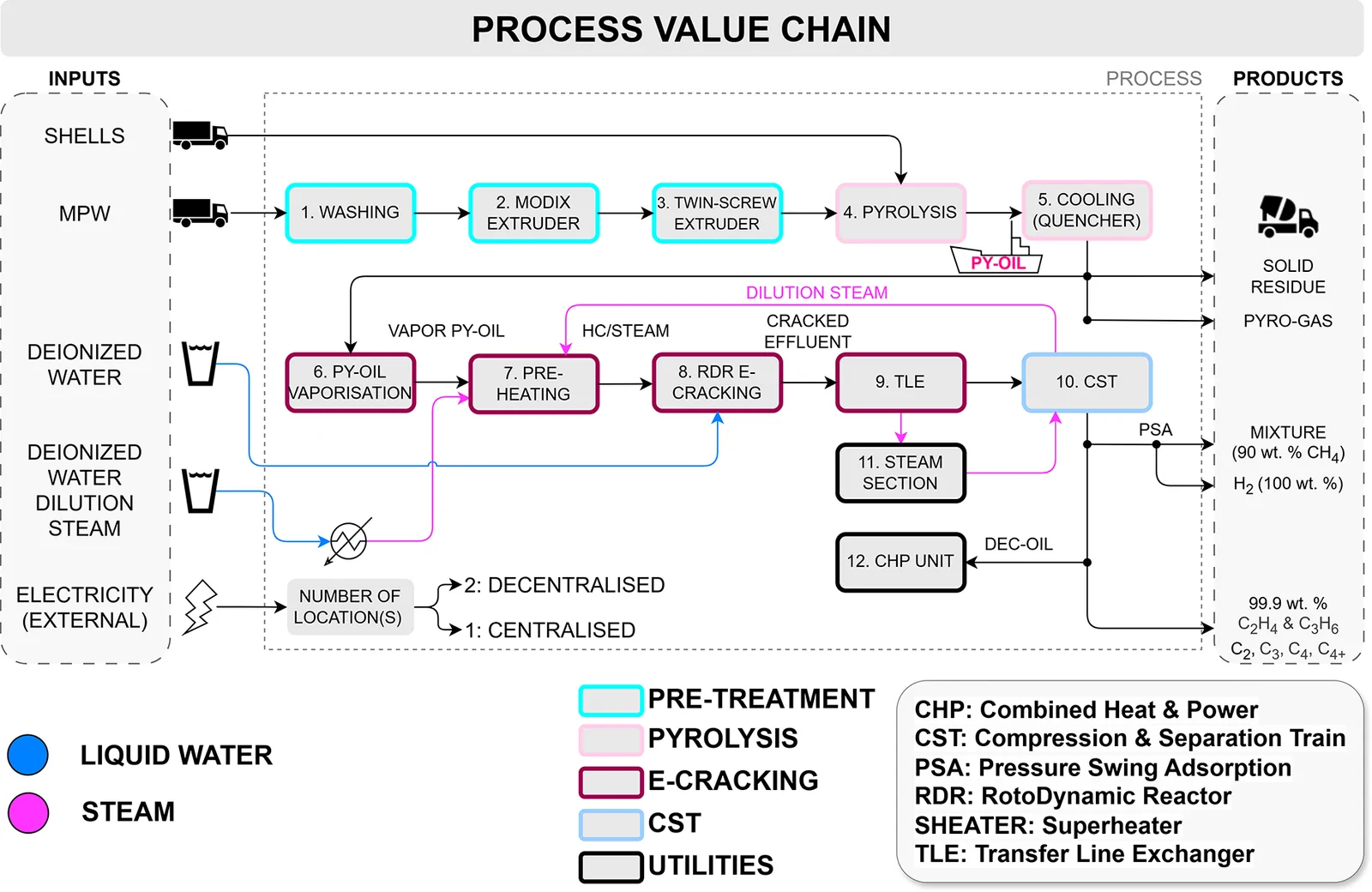
Value chain of the process. Key operating conditions (e.g., temperature, energy demand) for each step are provided in Section 2.3.

The process begins with pretreatment, which includes washing and extrusion. Washing removes surface contaminants and residual nonplastic fractions, while extrusion homogenizes the feed, removes volatile halogenated compounds, and densifies the waste stream into a form suitable for pyrolysis.

The pretreated MPW then undergoes electrified pyrolysis. In this step, the plastics are thermally decomposed into three main product streams: (i) a condensable liquid fraction (pyrolysis oil, PY-OIL), (ii) a noncondensable gaseous fraction (pyrolysis gas, PYRO-GAS), and (iii) a solid residue (SOLID-RESIDUE). The pyrolyser operates at ∼500 °C, consistent with typical plastic waste pyrolysis practice (∼400–1000 °C),^9^ with a PYRO-GAS outlet temperature of ∼430 °C. Crushed seashells containing calcium carbonate are added as a multifunctional medium: they improve heat distribution, provide abrasive action that reduces fouling, and may also capture impurities or exert a mild catalytic effect.^35,36^ The hot effluent is rapidly cooled in a quenching unit, where pyrolysis vapors are condensed into PY-OIL, while PYRO-GAS is separated and directed to downstream handling.

The liquid PY-OIL, the primary intermediate product, is heated in a vaporisation unit to 235 °C and subsequently mixed with superheated dilution steam at 200 °C. The resulting HC/steam mixture is preheated to 500 °C before entering the rotodynamic reactor (RDR). The RDR is an electrified steam-cracking technology based on turbomachinery designed to replace fossil-fired cracker furnaces in olefin production.^29,37^ Here, the cracked effluent contains the main target products, ethylene and propylene, as well as byproducts such as methane, butadiene and benzene. The cracked effluent is then rapidly cooled in a transfer line exchanger (TLE), where high-pressure saturated steam is generated (105 bar, 315 °C). This steam is subsequently superheated (∼500 °C) and integrated into the plant’s utility system for heating duties and, if necessary, for driving compressors via steam turbines.

PYRO-GAS can be directed to the CST in centralized configurations, flared, or valorised through industrial symbiosis in neighboring processes. SOLID-RESIDUE is recovered from the pyrolyser. Furthermore, hydrogen is separated via a pressure swing adsorption (PSA) unit. In contrast, a Combined Heat and Power (CHP) unit can use the decanter oil (DEC-OIL) to generate electricity and heat.

After cracking, the effluent enters the CST, where multistage compression and cryogenic distillation separate the cracked gas into polymer-grade ethylene and propylene (≥99.9 wt %). This section also yields the decanter oil used in the CHP along with a pure hydrogen stream and a methane-rich mixture.

The mass and energy balances for the process value chain are derived from literature data (i.e., pretreatment), experimental measurements (i.e., pyrolysis), or process simulations (e-cracking and CST), and they fall within typical ranges reported for comparable MPW-processing systems.^6,13,32,38,39^

A comparable agreement is also observed for the overall specific energy consumption, with the mixed plastic waste to ethylene route (39–43 MJ per kilogram of ethylene) lying within the range reported for existing mixed plastic waste to oil to olefins pathways, once results are harmonized to a common functional basis (Figure 2).^40,41^ To enable comparison, literature values are converted to MJ·kg C_2_H_4_^–1^ using representative ethylene yields where required. Lange et al.^40^ report a steam-cracking energy demand of 14–17 MJ·kg HVC^–1^; assuming typical ethylene yields of 30–45 wt % for plastic-derived pyrolysis-oil feeds,^34^ this corresponds to approximately 31–57 MJ·kg C_2_H_4_^–1^. Yadav et al.^41^ report a central supply chain energy demand of 46.3 MJ·kg C_2_H_4_^–1^ for catalytic fast-pyrolysis-based plastics-to-olefins conversion. When harmonized for comparison, an uncertainty range of 34–48 MJ·kg C_2_H_4_^–1^ is applied to reflect a typical ±15–20% variability arising from the cumulative uncertainties of multistep pyrolysis–cracking pathways and their underlying process-energy contributions, while retaining 46.3MJ·kg C_2_H_4_^–1^ as the central value. Although these studies differ in system boundaries, from a cracker-only analysis^41^ to a broader catalytic fast-pyrolysis cradle-to-gate system,^41^ they each include the core thermal cracking and separation steps that dominate the energy demand of plastics-to-olefins conversion, making them suitable for contextual comparison.

**Figure 2:**
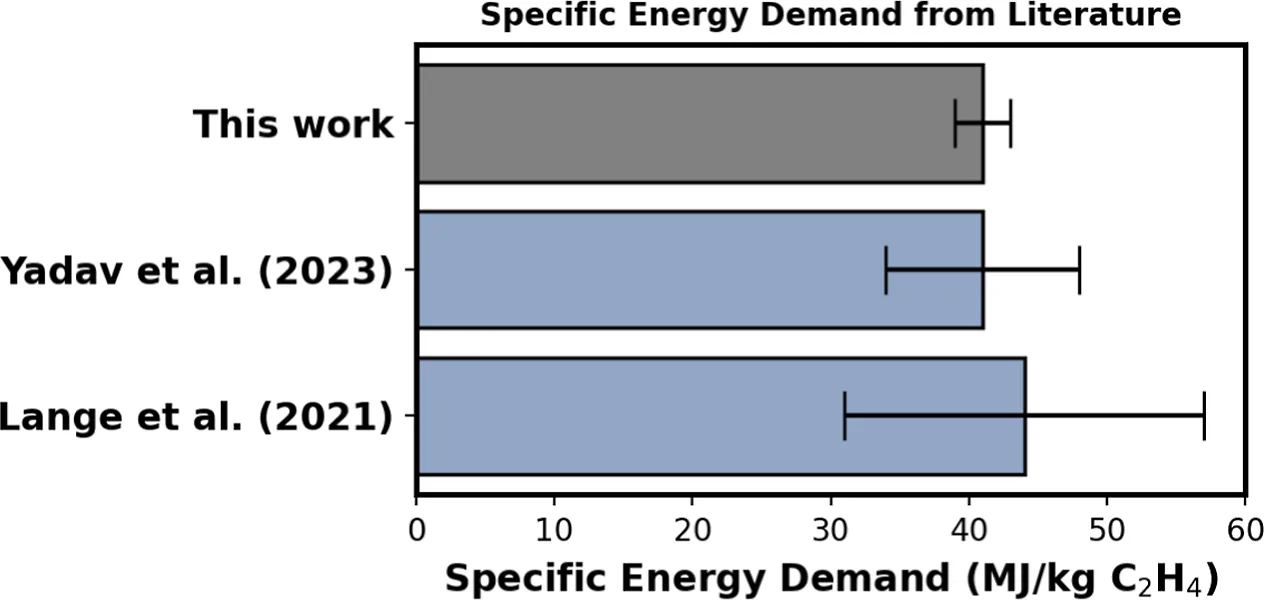
Specific energy demand of mixed plastic waste pyrolysis-and-cracking routes from the literature compared with this work. Error bars indicate reported or derived ranges for pyrolysis, upgrading, and cracking stages.^40,41^

The system configurations under investigation, along with details of the individual steps in the process value chain, are presented in Figure 3.

**Figure 3:**
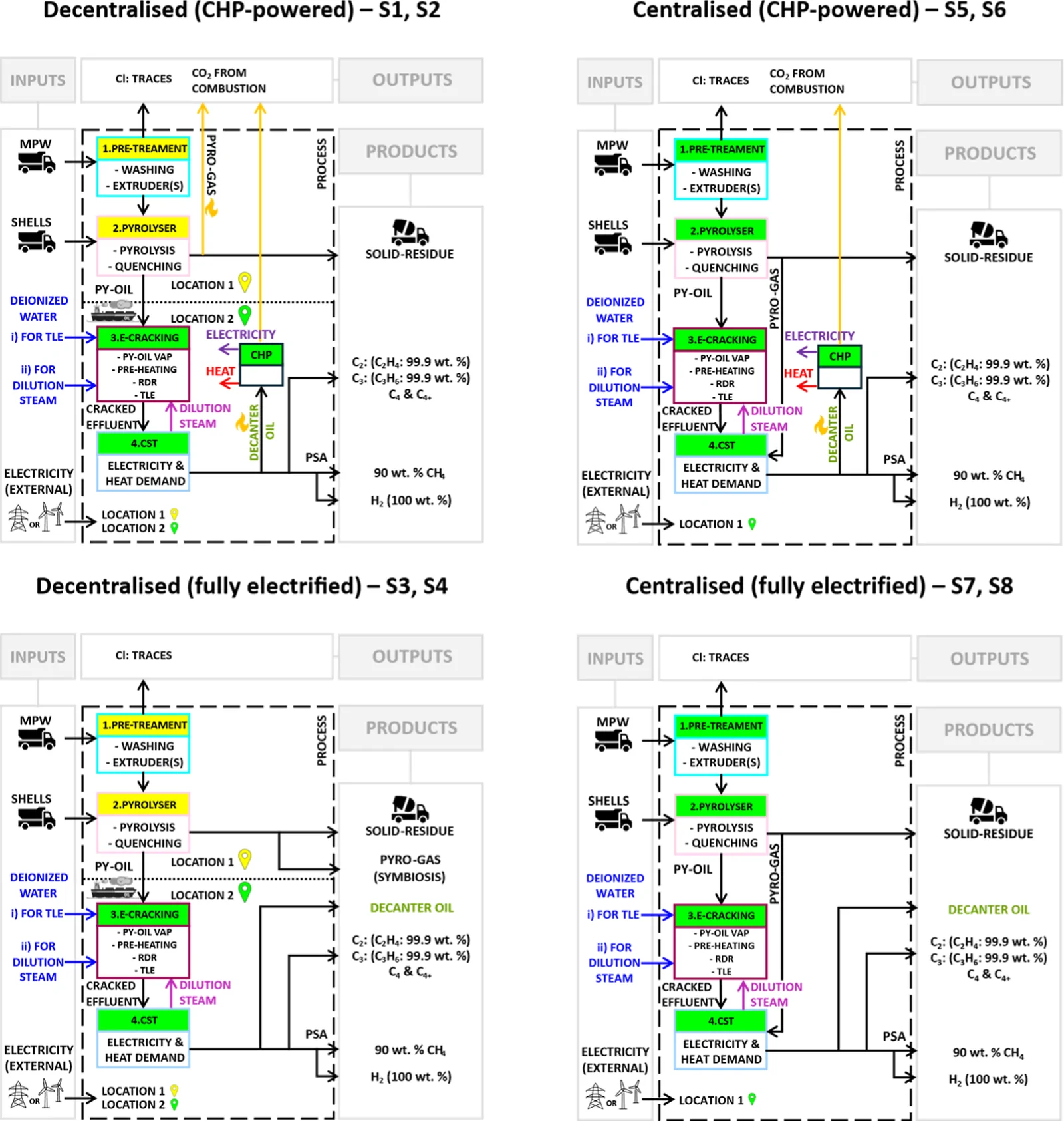
Simplified block scheme of the decentralised (left) and centralized (right) configurations under CHP-powered (top) and fully electrified (bottom) scenarios. In the decentralised configuration, yellow and green boxes indicate Location 1 (pretreatment and pyrolysis) and Location 2 (e-cracking and CST), respectively. In the centralized configuration, all process units are colocated at a single site and shown in green.

### 2.2 System Configuration & Scenarios

This study investigates eight scenarios that combine two process configurations (decentralised and centralized), two electrification levels (CHP-powered and fully electrified), and two electricity sources (grid and wind power), and different pyrolysis-product handling (i.e., PY-OIL, PYRO-GAS, SOLID-RESIDUE) factors, all of which significantly influence GHG emissions. Table 1 summarizes the key characteristics of each scenario. Products are streams treated as saleable or credited coproducts of the system (e.g., SOLID-RESIDUE, DEC-OIL, and PYRO-GAS when not combusted, polymer-grade olefins from the CST, pure hydrogen, and methane-rich mixture).

**Table 1:** Overview of Process Scenarios by configuration, Energy Source, and Pyrolysis Product Destination

scenario	configuration	electrification	electricity source	heat source	PY-OIL	PYRO-GAS	SOLID-RESIDUE	DEC-OIL
S1	decentralised	CHP-powered	grid/CHP	CHP	transported to RDR location	flared	byproduct	fuel at CHP
S2	decentralised	CHP-powered	wind/CHP	CHP	transported to RDR location	flared	byproduct	fuel at CHP
S3	decentralised	fully electrified	grid	–	transported to RDR location	byproduct	byproduct	byproduct
S4	decentralised	fully electrified	wind	–	transported to RDR location	byproduct	byproduct	byproduct
S5	centralised	CHP-powered	grid/CHP	CHP	integrated feed to RDR	input to CST	byproduct	fuel at CHP
S6	centralised	CHP-powered	wind/CHP	CHP	integrated feed to RDR	input to CST	byproduct	fuel at CHP
S7	centralised	fully electrified	grid	–	integrated feed to RDR	input to CST	byproduct	byproduct
S8	centralised	fully electrified	wind	–	integrated feed to RDR	input to CST	byproduct	byproduct

In the decentralised configuration, the pyrolyser is located at a separate site (Location 1) from the steam cracker and CST (Location 2). The PY-OIL is transported by tanker for further processing, while the PYRO-GAS is either flared on-site or treated as a valuable stream. In practice, this would require industrial symbiosis, in which nearby facilities (e.g., district heating plants, cement kilns, or combined heat and power units) could directly use the PYRO-GAS as a fuel or heat source. Such arrangements are only feasible when the pyrolysis site is colocated or in proximity to compatible industries, minimizing the need for costly gas compression and transport infrastructure. The SOLID-RESIDUE is treated as a potential byproduct for cement applications.^42,43^

The decentralised scenarios are defined as follows.

- Scenario 1 (CHP-DEC S1): CHP-powered (CHP heat and grid electricity).
- Scenario 2 (CHP-DEC S2): CHP-powered (CHP heat and wind electricity).
- Scenario 3 (FE-DEC S3): Fully electrified (grid electricity).
- Scenario 4 (FE-DEC S4): Fully electrified (wind electricity).

In scenarios 1–2, a CHP unit combusts DEC-OIL to cogenerate heat and electricity. Steam is generated via heat recovery in the TLE and subsequently superheated using hot flue gases from DEC-OIL combustion in the CHP. The same flue-gas stream is also used for PY-OIL vaporisation and for generating dilution steam via the dedicated HEX-DELW heat exchanger. As the steam produced in the TLE is insufficient to fully meet the combined requirements of steam-driven compressors and CST heating duties, additional steam is supplied by an auxiliary boiler. The resulting superheated steam is expanded through a two-stage pressure let-down system to supply medium-pressure (40 bar) and low-pressure (11 bar) steam for CST heating duties. Electricity produced in the CHP unit is distributed across the value chain, while any electricity deficit is supplied externally from the grid in Scenario 1 or onshore wind in Scenario 2. These CHP-powered configurations are illustrated in Figure 3 (top).

In scenarios 3–4, the CHP unit is eliminated and all compressors are electrically driven. Under these conditions, the high-pressure steam generated in the TLE, used exclusively as a heat-transfer medium for CST heating, is slightly superheated. Fully electrified operation, therefore, refers to the elimination of combustion-based heat and mechanical drives, while retaining steam as a heat-transfer medium generated without fuel combustion. Electricity is supplied entirely by the grid (Scenario 3) or onshore wind power (Scenario 4). The fully electrified configurations are shown in Figure 3 (bottom).

In the centralized configurations, all major processing units, including the pyrolyser, RDR steam cracker, and CST, are colocated at a single site. This configuration eliminates the need for PY-OIL transportation, and enables direct thermal and material integration, including the use of PYRO-GAS within the CST rather than flaring. The SOLID-RESIDUE remains a potential byproduct, e.g., for cement applications.

The centralized scenarios are defined as follows.

- Scenario 5 (CHP-CEN S5): CHP-powered (CHP heat and grid electricity).
- Scenario 6 (CHP-CEN S6): CHP-powered (CHP heat and wind electricity).
- Scenario 7 (FE-CEN S7): Fully electrified (grid electricity).
- Scenario 8 (FE-CEN S8): Fully electrified (wind electricity).

In scenarios 5–6, DEC-OIL–fired CHP supplies process heat and part of the electricity demand, with the remainder drawn from the grid or wind, whereas in scenarios 7–8 the CHP is eliminated and all mechanical energy is electrified, with process heat covered by internally recovered steam (Figure 3-right).

Across all scenarios, the TLE provides the baseline high-pressure (i.e., 105 bar) steam directly from heat recovery, while the key difference is whether supplementary steam generation and superheating rely on CHP flue gas (CHP-powered) or on electrically driven units such as electric boilers (fully electrified). The fully electrified configurations represent idealized decarbonisation scenarios, reflecting the maximum GHG reduction potential achievable under low-carbon electricity supply conditions. In this study, renewable-powered cases assume a constant and fully reliable supply of grid-connected wind-based electricity and do not account for renewable intermittency or hourly dispatch constraints.

### 2.3 Foreground LCI (Life-Cycle Inventory): Data Sources & Collection

Process data for the pretreatment section are obtained from the literature and falls within typical ranges.^9,44,45^ The pyrolysis inventory is based on experimental data, while the PY-OIL composition is characterized through comprehensive GC × GC analysis and subsequently used as input to the process model.^46,47^ The steam cracking section is rigorously modeled using COILSIM1D,^47^ which incorporates the detailed CRACKSIM kinetic framework specifically adapted for PY-OIL cracking. The steam cracker model accounts for elementary radical reaction kinetics and enables prediction of cracked-gas yields, product distributions, fuel demand, steam generation, as well as overall mass and energy balances under different operating conditions. The overall process modeling framework, including the compression and separation train (CST), is implemented in Python using in-house developed simulation tools. Within this framework, literature-derived, experimental, and model-generated inputs are integrated to calculate stream properties, unit operations, heat integration, and recycle loops based on rigorous thermodynamic and process-engineering equations. The framework can additionally incorporate kinetic, empirical, or machine-learning-derived correlations, enabling its application under both data-rich and first-principles-limited conditions.^6,9,13,32,38,39^

The data collection approach is presented in Figure 4.

**Figure 4:**
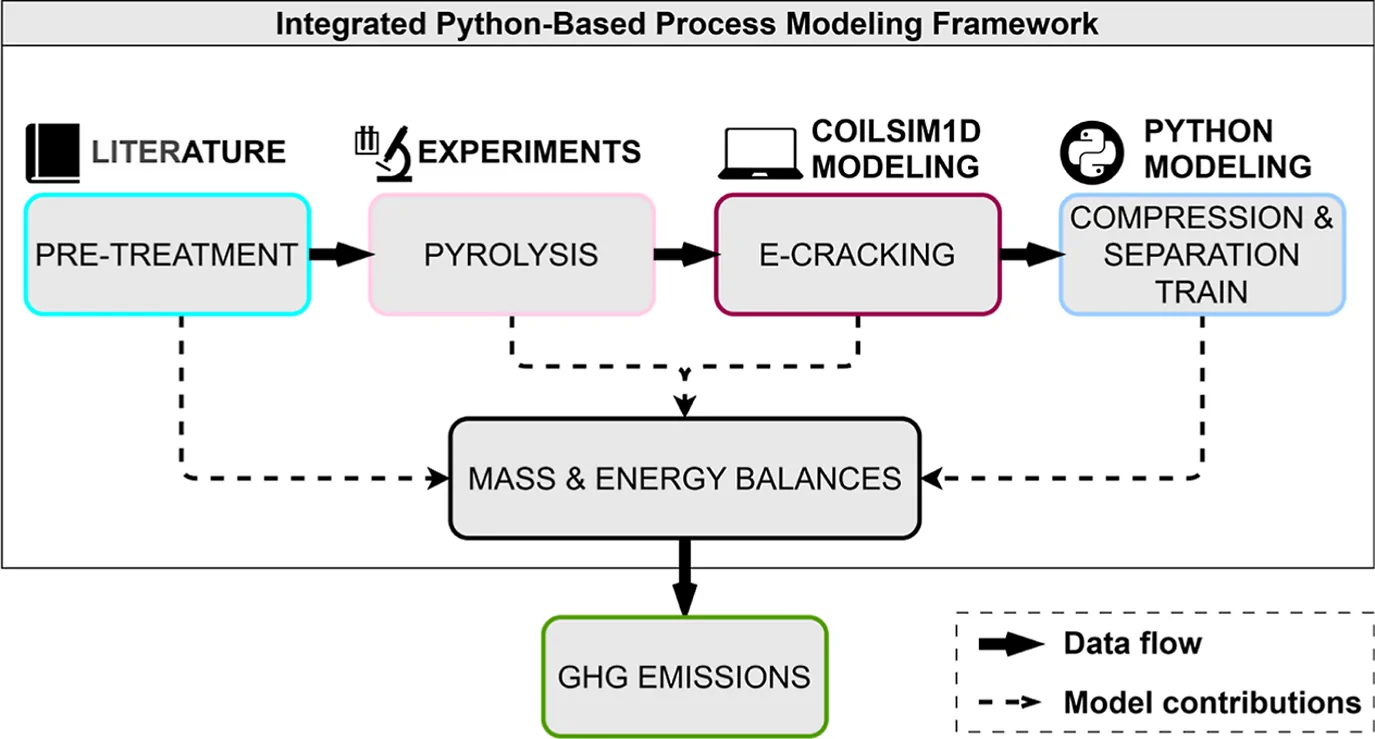
Data collection and modeling workflow adopted in this study. Pretreatment data are sourced from literature, pyrolysis data are obtained from experimental measurements, and the e-cracking and CST sections are modeled using process simulations. Mass and energy balances from each block are subsequently used to assess greenhouse gas (GHG) emissions.

#### 2.3.1 Pretreatment

The total electricity demand for the extrusion units is 500 kWh·t MPW^–1^, consistent with literature values for mechanical conditioning and high-temperature extrusion of mixed plastic waste.^9,44,45^ These inputs are summarized together with the pyrolysis outputs in Table 2.

**Table 2:** Mass Flows and Energy Requirements for the Pre-Treatment and Pyrolysis of MPW. 4281 kg h^–1^ of MPW are used for Normalisation

description	mass flow rate (kg·t MPW^–1^)	description	energy requirements (·t MPW^–1^)
MPW ^(IN)^	1000	extruder	500.0
PY-OIL ^(OUT)^	747.5	pyrolyser	350.0
PYRO-GAS ^(OUT)^	150.0	pumps (pyrolysis)	150.0
SOLID-RESIDUE ^(OUT)^	130.0		
shells ^(IN)^	27.5		
chlorides	traces		

#### 2.3.2 Pyrolysis

The pyrolysis inventory is based on experimentally validated operating conditions obtained from an electrified, continuous pilot-scale pyrolysis unit. A detailed description of the experimental setup and operating procedure is provided elsewhere.^48,49^ Briefly, prior to operation, the reactor and downstream condensation system are purged with nitrogen to ensure inert conditions. The condensation section consists of two water-filled condensers maintained at 45 °C (first condenser) and 5 °C (second condenser). The MPW feedstock is melted in a single-screw extruder at approximately 280 °C and continuously fed to the pyrolysis reactor via a heated transfer line.

The reactor operates at 450 °C, and once feeding stops, it is held at temperature for 1 h to ensure complete decomposition of the remaining material. Crushed seashells (27.5 kg·t MPW^–1^) are added directly to the pyrolyser as a CaCO_3_-based medium, consistent with literature on additive-assisted waste-plastic pyrolysis.^35,36^ PY-OIL is recovered from the condensation section, while PYRO-GAS is sampled and analyzed using a Refinery Gas Analyzer. The mass composition of PYRO-GAS is shown in Table S1. The PYRO-GAS is primarily comprised of propylene and C_4_ hydrocarbons.^44,50^ Solid residue is collected after reactor cooldown. The reduction of contaminants (i.e., heteroatom- and halogen-containing species) associated with the MPW feed prior to pyrolysis is facilitated by the two in-series extrusion stages. GCxGC-based analysis shows that nitrogen-containing species are below the analytical limit of detection, chlorine-containing species are below the limit of quantification, bromine-containing species are below the limit of detection, and sulfur is present only at low ppm concentrations. Since the thermal conversion route considered in this work is based on noncatalytic steam cracking, the system’s sensitivity to residual heteroatom species is expected to be significantly lower than that of catalytic upgrading processes. Furthermore, sulfur-containing compounds at ppm concentrations are generally compatible with conventional steam cracking practice, where sulfur-containing compounds are commonly used to passivate active nickel sites in cracking coils.^39^ Any H_2_S formed during downstream processing is subsequently removed in the acid-gas treatment section upstream of drying and cryogenic separation.^39^ Consequently, the experimentally characterized PY-OIL^19,51,52^ used in this study exhibited sufficiently low contaminant levels for explicit modeling of heteroatom-containing species to be neglected.

Since the MPW feed is pretreated prior to pyrolysis and the conversion route is noncatalytic, feedstock variability is expected to primarily influence the detailed composition of the produced PY-OIL and PYRO-GAS rather than the overall liquid yield. Experimental studies on polyolefin-rich MPW streams have reported relatively similar yields of condensable products (typically around 85–89 wt % within a reported experimental uncertainty of approximately 10%). At the same time, the main differences lie in hydrocarbon composition and contaminant levels.^52,53^ In addition, previous studies have shown that pretreated or washed waste streams can exhibit similar condensable-product yields despite significant reductions in ash content, suggesting that noncatalytic pyrolysis is relatively robust toward residual inorganic impurities.^52^ Nevertheless, substantially different feed compositions, particularly those containing higher fractions of PET, PVC, multilayer materials, or residual contaminants, may affect product distributions and downstream processing requirements.^52,53^

System-dependent routing of PYRO-GAS and downstream handling of PY-OIL are treated according to the decentralised and centralised configurations defined earlier.

The combined energy demand of pretreatment and pyrolysis is approximately 1.0 MWh·t MPW^–1^, consistent with published ranges for MPW pyrolysis systems.^9^ The mass and energy balances used in the LCI are summarized in Table 2.

#### 2.3.3 e-Cracking

The condensed PY-OIL is heated (at ∼ 235 °C) before entering the RDR. This vaporisation step requires ∼ 114 kWh·t MPW^–1^. The composition of PY-OIL used for modeling is in line with literature data. It includes paraffins (12.8 wt %), isoparaffins (3.1 wt %), linear olefins (17.9 wt %), branched olefins (42.3 wt %), linear diolefins (3.2 wt %), branched diolefins (9.7 wt %), naphthenes (6.8 wt %), monoaromatics (3.9 wt %), naphthenoaromatics (0.3 wt %), and diaromatics (4.1 × 10^–2^ wt %).^51,52^

Prior to e-cracking, the PY-OIL is mixed with dilution steam at a steam-to-PY-OIL mass ratio of 0.4,^34,54^ and the resulting hydrocarbon/steam mixture is preheated from ∼208 °C to ∼ 500 °C before entering the RDR (∼265.0 kWh·t MPW^–1^). The simulated cracked-gas composition is presented in Table 4, obtained using COILSIM1D. The cracking severity is optimized by maximizing the combined weight fraction of ethylene (26.75 wt %) and propylene (17.92 wt %).

**Table 3:** Mass Flows and the Energy Requirements for the e-Cracking and Cooling in TLE of PY-OIL. 4281 kg·h^–1^ of MPW are used for Normalisation

description	mass flow rate (kg·t MPW^–1^)	description	energy requirements (kWh·t MPW^–1^)
PY-OIL ^(IN)^	747.5	PY-OIL vaporisation	∼95.0
dilution steam ^(IN)^	299.0	HC/steam preheating	265.0
cracked RDR effluent ^(OUT)^	1046.5	RDR	710.0
deionized water (TLE inlet) ^(IN)^	598.0		
high pressure steam (HPS – steam drum) ^(OUT)^	598.0		

**Table 4:** Cracked RDR Effluent Composition Dry Basis

component	wt %	component	wt %
H_2_	0.96	C_4_H_10_	0.24
CO	0.02	C_4_H_8_	3.52
CH_4_	9.41	C_4_H_6_	8.21
C_2_H_4_	26.75	C_6_H_6_ (benzene)	6.65
C_2_H_6_	2.65	C_7_H_8_ (toluene)	2.32
C_2_H_2_	0.13	C_8_H_10_ (ethylbenzene)	0.08
CO_2_	0.00	C_8_H_10_ (xylenes)	5.42
C_3_H_6_	17.92	C_8_H_8_ (styrene)	0.58
C_3_H_8_	0.54	C_5_ - C_9_ (pygas)	9.81
C_3_H_4_	0.52	C_10+_ (fuel oil)	4.28

The electricity demand, at optimized cracking conditions for the electrified RDR corresponds to approximately 710 kWh·t MPW^–1^. Inventory data for PY-OIL input, dilution steam, and cracked effluent flows are summarized in Table 3.

Following e-cracking, the effluent is rapidly quenched in the transfer line exchanger (TLE). Thermal energy recovered from the hot effluent is transferred to approximately 598 kg·t MPW^–1^ of boiler-feedwater (105 bar, 68 °C), producing saturated high-pressure steam (105 bar, 315 °C) and corresponding to ∼403 kWh·t MPW^–1^ of recovered thermal duty.

In CHP-powered configurations, an auxiliary boiler provides boiler feedwater. The generated steam is then superheated to ∼510 °C, requiring an additional 170–190 kWh·t MPW^–1^, and integrated into the plant’s utility system.

#### 2.3.4 Compression & Separation Train

The CST represents the central focus of the process design, as it governs key separation, compression, and recycle operations that ultimately determine the feasibility and performance of the overall MPW-to-olefins system. To capture this, the CST block is modeled using an in-house simulation framework based on the Peng–Robinson equation of state (PREOS) for thermodynamic property estimation of hydrocarbon mixtures, while steam properties are calculated using the IAPWS-95 formulation.^55,56^ PREOS provides reliable vapor–liquid equilibrium predictions for light and heavy hydrocarbons, whereas IAPWS-95 is the reference-standard equation of state for water and steam and offers significantly higher accuracy in the high-pressure and high-temperature ranges relevant for the process steam balance. Both decentralised and centralised layouts follow a demethaniser-first flowsheet with tail-end hydrogenation, as commonly applied in industrial steam cracking systems.^9,17,39,57,58^

As illustrated in Figure 5, the RDR cracked gas is first quenched in oil (Q-101) and water towers (Q-102), with overhead gases (C_1_–C_5_) routed to a five-stage compression train (C-101 to C-105) and bottom liquids sent to a decanter. The compressed gas mixture is then separated in a sequence of distillation columns (demethaniser, deethaniser, C2 splitter, depropaniser, debutaniser, C3 splitter) and two hydrogenation reactors (R-201, R-202) that remove acetylene and MAPD under equilibrium conditions. Ethane and propane are recycled to a dedicated steam cracker, whose effluent is treated as a fixed-composition inlet to the CST in this work. In the centralised configuration, PYRO-GAS is additionally introduced as an input stream, enabling higher integration and reducing flaring losses (Figure 5-bottom). Distillation columns and hydrogenation reactors are optimized through sensitivity analyses of key variables, such as the number of stages, operating pressures, and reflux ratios, to ensure polymer-grade purity (≥99.9 wt % ethylene and propylene) and an optimal operating range. The PSA unit is treated as part of the CST simulation, and the separation of the hydrogen–methane mixture (stream T201-GA) is modeled using the same Python-based framework employed for the CST. The CST model also explicitly accounts for the cryogenic refrigeration duties associated with the propylene and ethylene refrigeration cycles, including compressor electricity consumption, flash separation, expansion, and low-temperature condensation requirements necessary for polymer-grade olefin recovery. In addition, hydrogen consumption associated with the front-end hydrogenation reactors is accounted for in the CST mass and energy balances. These utility demands are subsequently included in the overall electricity consumption and cradle-to-gate GHG assessment. Additional subsystem descriptions and detailed stream data are provided in Section 3.1.

**Figure 5:**
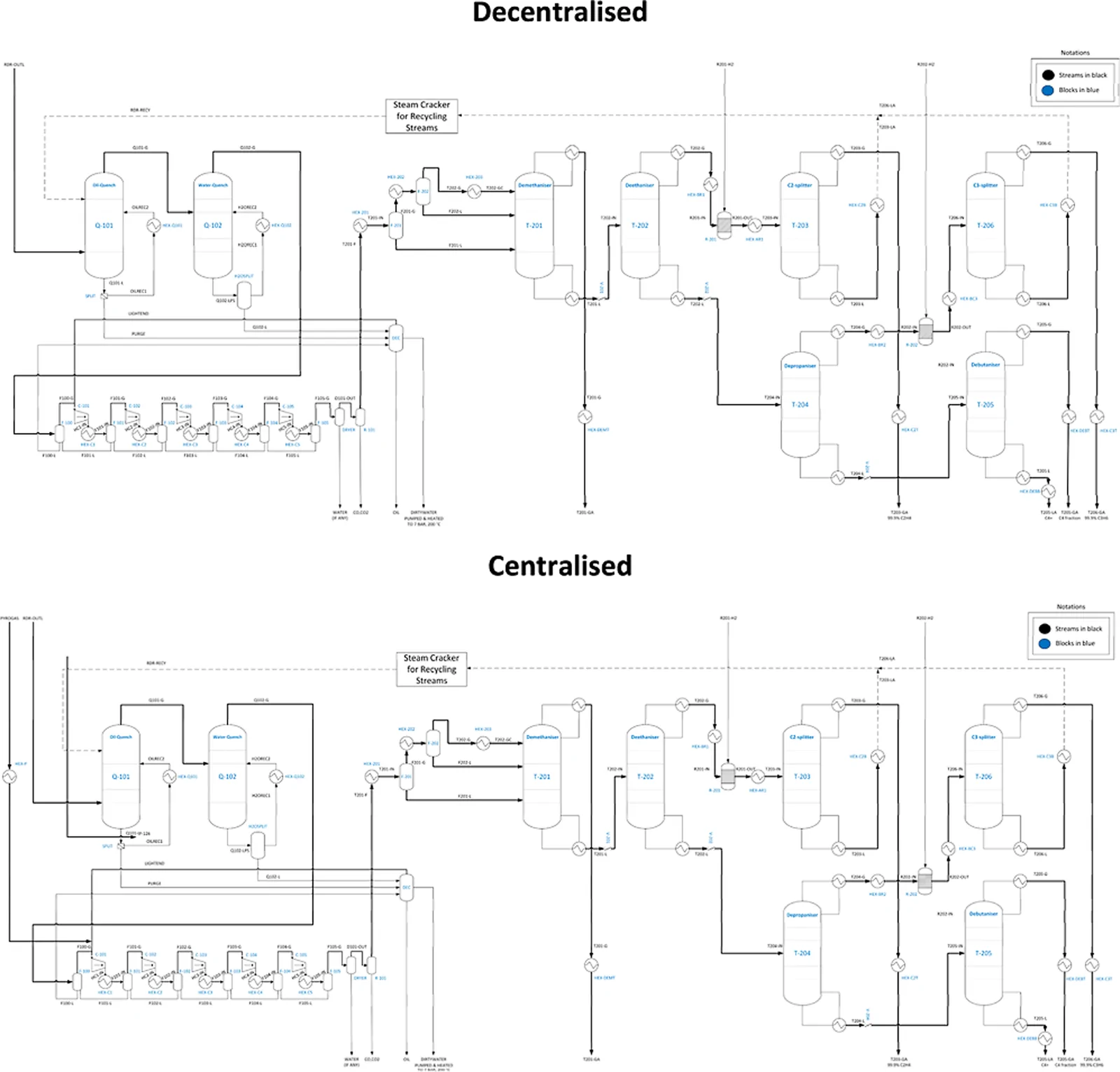
sPFD of the CST for the decentralised (top) vs centralised (bottom) process.

The CST balances are generated using custom Python models based on first-principles thermodynamics and process calculations^59^ (see Equations for Developed Code in Supporting Information). The resulting heating and cooling duties are later evaluated through pinch-based energy analysis,^60,61^ with detailed results presented in the Technical Results section.

### 2.4 Life-Cycle GHG Emissions

Following the completion of the process design and the collection of all required mass and energy balances, an LCA is carried out to quantify the GHG emissions associated with each system configuration. The analysis focuses on the climate change impact category. The LCA follows the ISO 14040 and ISO 14044 guidelines,^62–64^ encompassing the four standard phases: (i) goal and scope definition, (ii) life-cycle inventory (LCI), (iii) life-cycle impact assessment (LCIA), and (iv) interpretation of results. Environmental burdens are allocated among saleable products using a mass-based allocation approach, consistent with the attributional LCA framework applied in this study. This method is selected for its physical transparency when dealing with material coproducts, while avoiding the price volatility associated with economic allocation and the limited relevance of energy-based allocation for chemical products.^63^

Sensitivity analyses are conducted on key parameters, particularly electricity supply and MPW transportation distance, due to their dominant influence on overall GHG emissions. Consequently, the absolute environmental impacts reported may vary depending on feedstock characteristics, electricity mix, logistics conditions, and future infrastructure and energy-system developments.

#### 2.4.1 Goal and Scope

This study aims to quantify the GHG emissions and identify the key environmental drivers of the process, under both decentralised and centralised configurations. As the process represents a pathway for converting MPW into high-value olefins, particular emphasis is placed on evaluating how the electricity source and electrification level influence its carbon footprint (i.e., CO_2‑eq_).

To capture these design and energy variations, eight distinct scenarios are examined, combining.

- Two process configurations: decentralised and centralised,
- Two electrification levels: CHP-powered (with steam-driven compressors) and fully electrified (with electric compressors),
- Two electricity sources: (1) grid electricity (EU mix), (2) renewable electricity (onshore wind power, Netherlands). Onshore wind electricity is selected as a representative low-carbon renewable electricity source to highlight the GHG emission reduction potential of the electrified MPW-to-olefins value chain. The renewable-powered scenarios assume a constant, and fully reliable supply of grid-connected wind-based electricity, rather than direct physical coupling between the process and the wind turbines.

The scenario matrix and its key defining features are summarized in Table 1.

To evaluate the environmental advantages of the process, a sensitivity analysis is first performed (Section 3.3) using a functional unit of kg HVC^–1^, which enables the assessment of specific process variables such as transportation impacts. Subsequently, a benchmarking analysis is conducted (Section 4) to compare the system’s performance against (i) conventional MPW management pathways, landfilling, incineration, and open burning, and (ii) established or emerging HVC production technologies, including naphtha and ethane steam cracking, oxidative coupling of methane, and pyrolysis-based routes (followed by steam cracking or catalytic upgrading). The benchmarking employs two main functional units: t MPW^–1^ processed and kg HVC^–1^ produced.

An attributional cradle-to-gate LCA focusing on the GHG emissions impact category is conducted in accordance with ISO 14040 and 14044 standards. The system boundaries include both foreground and background processes: MPW transportation from collection sites to the pyrolysis unit, the full CST, and upstream utility supply (e.g., electricity, deionized water), up to the final product delivery (olefins) meeting target specifications.

#### 2.4.2 System Boundaries & Functional Units

The system boundaries cover all foreground operations in the process, upstream supply of electricity, water and materials, transportation of MPW, PY-OIL (decentralised), and final product delivery (olefins and byproducts).

Based on the available data, the following assumptions are made for estimating GHG emissions.

- MPW and shells are considered waste with zero upstream burden; only transport emissions are included.
- MPW and PY-OIL (if decentralised) are transported 100 km by truck and tanker, respectively; shells travel 25 km.
- DEC-OIL is combusted in a CHP unit, achieving approximately 35% electrical and 50% thermal efficiencies in CHP-powered cases.^65,66^ Electricity generated in the CHP unit is treated as internally produced energy and does not receive avoided-burden credits against the grid.
- The handling and utilization of pyrolysis products (PY-OIL, PYRO-GAS, SOLID-RESIDUE,^67,68^ DEC-OIL) varies across scenarios depending on the configuration and electrification level, as detailed in Table 1.
- CHP flue gases (∼650 °C) are utilized as a high-temperature heat source across multiple process heating duties, including steam generation, steam superheating and PY-OIL heating.^65,66^ The flue gas contains CO_2_: 13.4,H_2_O: 11.1, N_2_: 73.5, and O_2_: 2 wt %.^69^
- Superheated and dilution steam loops assume 5% water loss requiring deionized water makeup.
- Foreground mass and energy flows are derived from internal process simulations and literature sources. Background inventory data are sourced from ecoinvent v3.10.1 (cutoff system model), attributing burdens to the primary waste producer. Accordingly, MPW is treated as a burden-free waste stream, attributing the environmental impacts fully to the waste producer. However, the MPW treatment activities, as depicted in Figure 1, are included within the boundary limits of this work. The cutoff approach is consistent with the attributional LCA framework and is commonly applied to model waste-derived systems and recycling pathways.^62,63,70^ A full list of data sets is provided in Table S14.

Two functional units are defined to enable a comprehensive evaluation of system performance.

- 1 tonne of MPW processed, used for comparison against alternative waste management routes (e.g., landfilling, incineration).
- 1 kg of high-value chemicals (HVCs: ethylene, propylene, benzene, hydrogen, and butadiene)^9^ produced, used to benchmark against conventional and emerging olefin production technologies. For this study, the carbon-intensity reference for naphtha-based olefin and HVC production, taken as the current best available technology (BAT), is 1.0 kg CO_2‑eq_·kg HVC^–1^.^9^ Using this baseline, the EU’s climate-reduction targets can be translated into process-specific benchmarks. A 55% reduction corresponds to approximately 0.45 kg CO_2‑eq_·kg HVC^–1^,^71^ and a 90% reduction corresponds to approximately 0.10 kg CO_2‑eq_·kg HVC^–1^.^72^

The environmental burdens are distributed among the process output streams using mass-based allocation, consistent with an attributional LCA framework. In this approach, all saleable products contributing to high-value chemicals (HVCs) jointly form the allocation basis. In contrast, streams that are combusted or flared (e.g., DEC-OIL in CHP-powered scenarios or PYRO-GAS in decentralised configurations) are excluded. Byproducts that retain material value are treated as coproducts and included in the allocation in proportion to their mass contribution.

When impacts are subsequently normalised per kilogram of HVC, the resulting impact intensity is identical for all coproducts included in the allocation basis, i.e., each kilogram of product carries the same share of the total system-wide emissions. Differences in total allocated impacts therefore arise solely from differences in product mass. In contrast, scenario-dependent variations in product valorisation (e.g., whether PYRO-GAS or DEC-OIL are flared, reused, or credited as byproducts) are reflected in the inclusion or exclusion from the allocation basis. This approach ensures physical consistency and comparability between decentralised and centralised configurations.

#### 2.4.3 Geographical Boundary

Background processes such as transportation and grid electricity (market group for high-voltage electricity) are representative of the European market. For deionized water, Europe excluding Switzerland is considered, while for renewable electricity supply (electricity production, wind, > 3MW turbine, onshore), The Netherlands market is considered.

#### 2.4.4 Impact Category

Based on the Environmental Footprint v3.1 (EF v3.1) method of the European Commission,^73^ the climate change impact category is chosen for the LCIA of this study. The environmental indicator of the climate change impact category, along with its measurement unit is summarized in Table 5.

**Table 5:** Climate Change Impact Category is Included in EF v3.1 Method, and the Indicator Used to Assess it

impact category	indicator	unit
climate change (CC)	global warming potential (GWP100)	kg CO_2‑eq_

## 3 Results and Discussion

### 3.1 Technical Results

The technical performance indicators for the decentralised and centralised MPW-to-olefins configurations are summarized in Figure 6 and presented in detail in Figures S7–S18. The corresponding carbon footprints are also reported in these figures and are discussed in Section 3.2.

**Figure 6:**
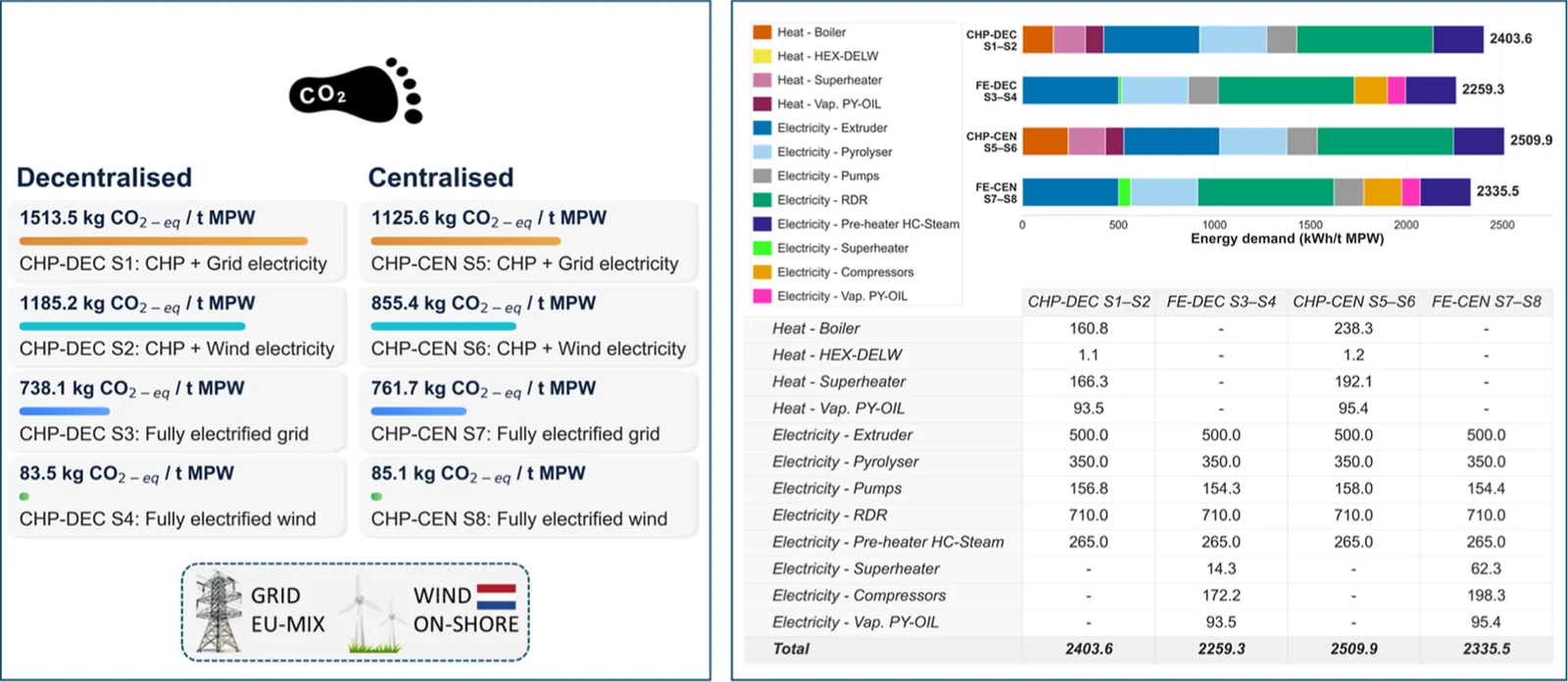
Comparison of the investigated scenarios, as presented in Table 1. The left panel summarizes the total life-cycle GHG emissions per tonne of mixed plastic waste (MPW). In contrast, the right panel presents the corresponding breakdown of process energy demand by unit operation and utility contribution. CHP-powered cases include steam generation (e.g., steam-driven compressors) and superheating through CHP integration, whereas fully electrified cases rely exclusively on electricity-driven operation. Values are normalised per tonne of MPW processed.

Processing 1 tonne of MPW yields 747.5 kg of PY-OIL, approximately 150 kg of PYRO-GAS, and roughly 130 kg of solid residue. In addition, 27.5 kg·t MPW^–1^ of crushed seashells are incorporated as a functional pyrolysis medium. The decentralised and centralised configurations differ primarily in vapor routing: while the preheated HC/steam mixture enters the RDR in both layouts, only the centralised configuration additionally routes PYRO-GAS to the CST together with the cracked effluent. This difference increases hydrocarbon throughput in the centralised scenario and directly translates into higher olefin yields. Across both configurations, the CST produces 203–213 kg·t MPW^–1^ of ethylene and 134–166 kg·t MPW^–1^ of propylene. These values correspond to total olefin yields of 338 kg·t MPW^–1^ (33.8 wt.%) in the decentralised configuration and 380 kg·t MPW^–1^ (38.0 wt.%) in the centralised configuration, with the latter yielding approximately 5% more ethylene and 24% more propylene due to the additional integration of PYRO-GAS.

#### 3.1.1 Electricity Demand

Electricity consumption remains consistent across scenarios for several key operations. All configurations require ∼ 94–95 kWh·t MPW^–1^ for PY-OIL vaporisation, 265 kWh for preheating the HC/steam mixture to 500 °C, 710 kWh·t MPW^–1^ for the RDR, 500 kWh·t MPW^–1^ for pretreatment, and 500 kWh·t MPW^–1^ for pyrolysis. Pump electricity covers all pumping services across the pyrolysis block, CST, and steam-balance subsystem (Figure 6). Electricity use for PSA off-gas handling is negligible in all scenarios because the feed enters the adsorption step at 36 bar and no vacuum or recompression step is required.

#### 3.1.2 Heat Integration and Utilities Use

Pinch-based heat integration significantly reduces heating and cooling requirements across all configurations. The centralised layout benefits from a high-temperature PYRO-GAS stream, which provides a higher available duty for heat recovery and contributes to a larger absolute reduction in hot utility use compared to the decentralised configuration (393.0 vs 353.8 kWh·t MPW^–1^. The resulting Grand Composite Curves (Figure 7) highlight notable shifts in both hot- and cold-utility targets. They represent the two process configurations considered in this study: the decentralised GCC applies to Scenarios 1–4 (from Table 1), while the centralised GCC applies to Scenarios 5–8 (from Table 1), since the process hot and cold streams are determined primarily by the plant configuration. Differences in electrification level and electricity source mainly influence utility supply and associated GHG emissions rather than the underlying heat-recovery targets. As summarized in Table 6, heat integration reduces hot-utility demand by approximately 35–36% and cold-utility demand by 34–37%, depending on the configuration. Detailed thermal data underlying the pinch analysis are provided in the Supporting Information (Tables S4 and S5), while utility demands by temperature level are summarized in Table 7. Heating demand is dominated by steam generation and HC/steam mixture preheating duties, whereas cooling demand is mainly associated with cooling water and refrigeration cycles. The centralised configuration exhibits higher cooling utility requirements, particularly for cooling water (521.3 vs 403.0 kWh·t MPW^–1^ and the propylene stage of the refrigeration cycle (199.7 vs 172.7 kWh·t MPW^–1^, due to the additional PYRO-GAS cooling and separation load (Tables S9 and S13). All units operate above 1 bar to avoid vacuum conditions.

**Figure 7:**
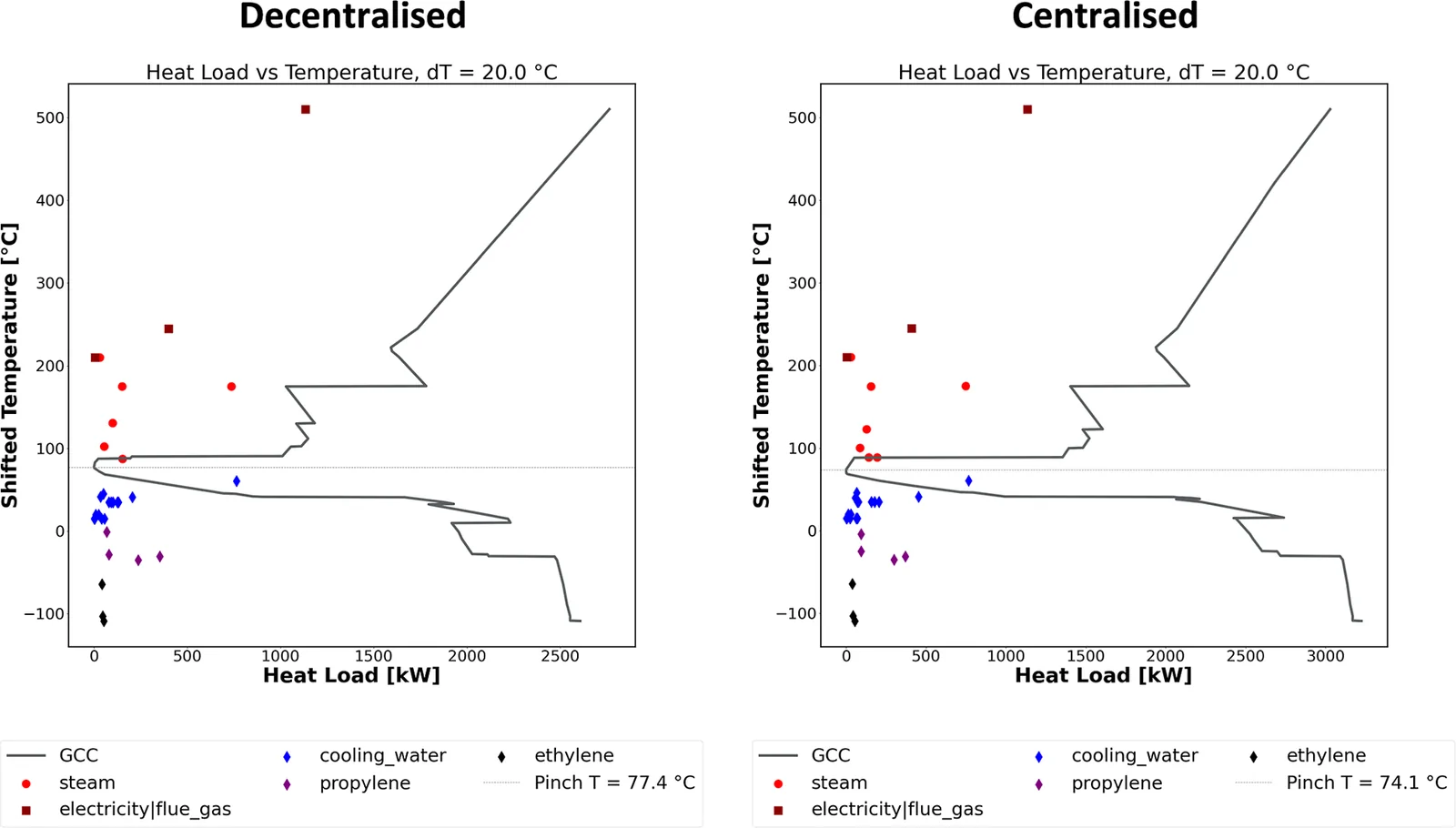
Grand Composite Curves (GCCs) for the decentralised configuration, representative of Scenarios 1–4, and the centralised configuration, representative of Scenarios 5–8. The dark gray line represents the GCC, the dashed light gray line indicates the pinch temperature, and the colored markers indicate the utility levels considered: dark red = electricity or CHP flue gas, red = MP/LP steam, blue = cooling water, purple = propylene refrigeration, and black = ethylene refrigeration. The *x*-axis position indicates heat load.

**Table 6:** Utility Demands with and without Integration^a^

utility type	drop	without integration (kWh·t MPW^–1^)	with integration (kWh·t MPW^–1^)
decentralised			
cold	37%	963.0	609.2
hot	35%	999.5	645.7
total		36.5	36.5
centralised			
cold	34%	1146.1	753.0
hot	36%	1100.3	707.3
total		45.8	45.8

a 4281 kg h^–1^ of MPW are used for Normalisation. Drop Indicates the Percentage Reduction in Utility Demand Achieved Through Heat Integration, Calculated Relative to the Corresponding Case without Integration.

**Table 7:** Overview of Utility Demands. 4281 kg h^–1^ of MPW are Used for Normalisation

utility type	typical use	decentralised (kWh·t MPW^–1^)	centralised (kWh·t MPW^–1^)
hot utilities (heating)			
electricity	HC/PY-OIL preheating (500 °C)	265.0	265.0
CHP flue gases (650 °C)	heat source steam generation and steam-superheating as well as PY-OIL vaporisation	94.6^a^	96.6
MP steam (40 bar)	up to 200 °C	214.6	217.6
LP steam (11 bar)	up to 121 °C	71.5	128.1
heaters’ demand		645.7	707.3
cold utilities (cooling)			
cooling water (1 bar)	down to 25 °C	403.0	521.3
propylene (1–13 bar)	25 to −48 °C	172.7	199.7
ethylene (1–13 bar)	–48 to −103 °C	33.5	32.0
coolers’ demand		609.2	753

a Corresponds to extra dilution steam (HEX-DELW) for RDR and PY-OIL vaporisation.

#### 3.1.3 CHP Fuel Input and Energy Supply Mode

In CHP-powered configurations, decanter oil serves as the fuel input for the cogeneration of flue-gas heat and electricity. The decentralised and centralised configurations consume 210 and 255 kg DEC-OIL·t MPW^–1^, corresponding to 2424 and 3001 kWh·t MPW^–1^ of calorific input, respectively. In both configurations, the resulting CHP flue gas provides the PY-OIL vaporisation duty in addition to supplying the heat required for steam superheating, boiler operation, and the generation of dilution steam via HEX-DELW.

#### 3.1.4 Steam Generation and Superheating

In CHP-powered scenarios, high-pressure steam generated in the TLE (105 bar, 315 °C) is further superheated in the SHEATER using CHP flue gas. When additional steam is required, supplementary steam is generated in the boiler, which is also heated by the hot CHP flue gases. In both decentralised and centralised configurations, a fraction of the available flue-gas heat is additionally recovered in HEX-DELW to supply the dilution steam required by the CST.

Taken together, the duties associated with the steam system (i.e., superheater, boiler, and dilution-steam heat exchanger) correspond to total steam demands of 1181.5 MJ·t MPW^–1^ in the decentralised configuration and 1553.6 MJ·t MPW^–1^ in the centralised configuration.

In fully electrified scenarios, compressors are electrically driven and the steam generated from the TLE is routed exclusively to CST heating, with no steam demand for power generation or mechanical drives. Consequently, the remaining steam-system duty (i.e., the electricity required for steam superheating) is drastically reduced, to 14.3 kWh·t MPW^–1^ in the decentralised configuration and 62.3 kWh·t MPW^–1^ in the centralised one. Although the centralised layout retains a higher CST thermal load, the associated steam requirements remain substantially lower than in the CHP-powered cases.

#### 3.1.5 Compressors Use

The influence of electrification on compressor energy use is clearly reflected in the CST electricity profile. In fully electrified configurations, compressor electricity demands amount to 172 kWh·t MPW^–1^ in the decentralised layout and 198 kWh·t MPW^–1^ in the centralised layout. In contrast, CHP-powered configurations do not explicitly report compressor electricity, as mechanical power is delivered through steam turbines and is therefore embedded in the steam-generation duties.

#### 3.1.6 Supporting Information Overview

Additional details on unit functions (Table S6), reactor conditions (Table S7), and mass balances used in the LCI (Table S8) and full subsystem descriptions of the Heat Exchanger Network (HEN), refrigeration, steam, and cooling-water sections (Figures S19–S23 and Tables S9–S13) are provided in the Supporting Information. The tables containing the detailed stream compositions are provided in a separate.xlsx file due to their large size.

#### 3.1.7 Synthesis of Findings and Link to GHG Analysis

Overall, process configuration determines the distribution of energy demand between process units. Electrification determines how this energy is supplied, shifting mechanical-drive and heating duties from steam and combustion to electricity. The implications of these differences for cradle-to-gate GHG performance, including the kg CO_2‑eq_ values for inputs and outputs shown in Figures 6 and S7–S18, are examined in detail in the Section 3.2.

To provide a consolidated comparison across all configurations, Table 8 summarizes key process outputs and total specific energy demands for the decentralised and centralised configurations under both CHP-powered and fully electrified operation.

**Table 8:** Comparison of Product Yields and Utility Demands for Decentralised and Centralised configurations

description	decentralised	centralised	unit
yields			
ethylene (99.9 wt.%)	203.43	213.24	kg·t MPW^–1^
propylene (99.9 wt.%)	134.17	166.32	
olefins	337.61	379.56	
specific energy demand			
CHP-powered			
total	2403.6	2509.9	kWh·t MPW^–1^
	25.6	23.8	MJ·kg_OLEF_^–1^
fully electrified			
total	2259.3	2335.5	kWh·t MPW^–1^
	24.1	22.2	MJ·kg_OLEF_^–1^

### 3.2 Life-Cycle GHG Emissions: Scenario Analysis

Figure 8 presents the GHG emissions per tonne of MPW processed across all process scenarios, comparing decentralised (top) and centralised (bottom) configurations under different electrification levels (CHP-powered vs fully electrified) and electricity sources (grid vs renewable/wind). Pie charts show the relative contribution of key emission sources: electricity, transportation of MPW, and direct process emissions (e.g., CHP combustion, PYRO-GAS flaring). It is important to note that PYRO-GAS is flared only in the decentralised, CHP-powered scenarios (1–2), contributing directly to scope 1 emissions. In the fully electrified decentralised scenarios (3–4), the PYRO-GAS is utilized through industrial symbiosis, serving as an energy carrier in adjacent processes, whereas in all centralised scenarios (5–8), it is directly injected downstream to the CST (see also Table 1). This variation in PYRO-GAS handling significantly influences the share of combustion-related emissions across scenarios.

**Figure 8:**
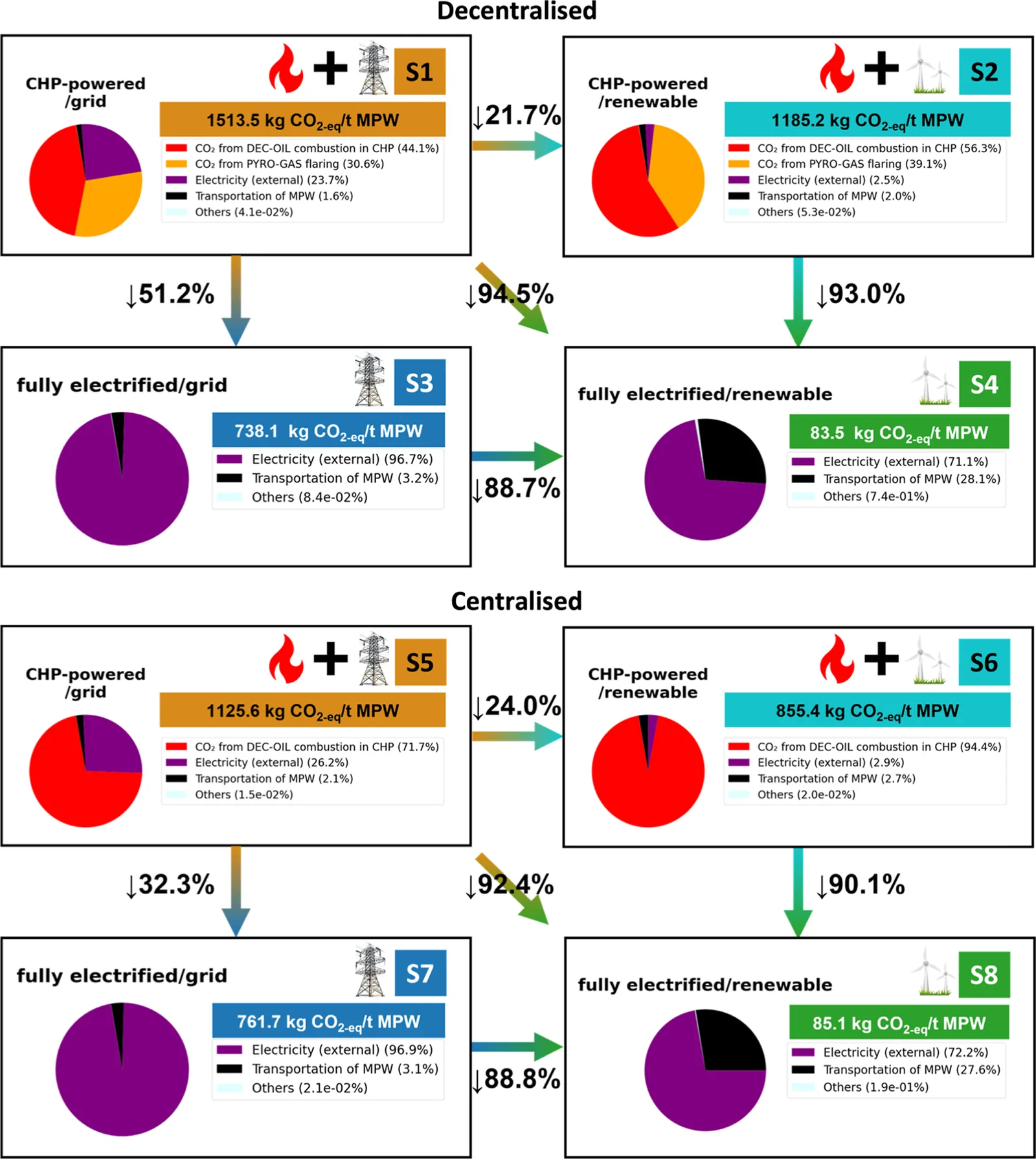
GHG emissions of process across eight scenarios: impact of configuration, electrification level, and electricity source (decentralised configurations at the top, centralised configurations at the bottom). Colored arrows indicate the percentage reduction in emissions achieved when transitioning between energy supply scenarios. A complete breakdown of all the categories (bar charts), including all minor sources, is reported in the Supporting Information (Figure S24).

In decentralised configurations, the CHP-powered/grid scenario represents the current nonoptimized state of the system, yielding 1513.5 kg CO_2‑eq_·t MPW^–1^. Emissions are dominated by direct combustion of DEC-OIL in the CHP unit (44.1%) and flaring of PYRO-GAS (30.6%). Replacing grid electricity with wind power (CHP-powered/renewable) results in a modest 21.7% reduction (1185.2 kg CO_2‑eq_·t MPW^–1^) due to the limited contribution of electricity to total emissions. Full electrification significantly improves performance: emissions drop by 51.2% to 738.1 kg CO_2‑eq_·t MPW^–1^ when powered by grid. Using renewable electricity further reduces emissions by 88.8%, reaching 85.1 kg CO_2‑eq_·t MPW^–1^, mainly from electricity (72.2%) and MPW transport (27.6%).

In centralised configurations, the CHP-powered/grid case results in 1125.6 kg CO_2‑eq_·t MPW^–1^, largely due to CHP emissions (71.7%) and electricity (26.2%). Switching to renewable electricity has a limited effect (−24.0%), yielding 855.4 kg CO_2‑eq_·t MPW^–1^. Fully electrifying the system brings substantial improvements: emissions fall to 761.7 kg CO_2‑eq_·t MPW^–1^ (−32.3%) and drop further to 85.1 kg CO_2‑eq_·t MPW^–1^ with renewable power, reflecting a 92.4% total reduction.

### 3.3 Sensitivity Analysis: MPW and PY-OIL Transportation (Scope 3) for Fully Electrified Renewable-Powered Configurations

To assess whether electrified pathways can meet EU decarbonisation milestones, the sensitivity analysis considers only the fully electrified, renewable-powered configurations, where process-related emissions are minimized and the contribution of scope 3 emissions becomes significant (i.e., ∼ 28%). This provides a stringent test of whether Scope 3 transport emissions could undermine alignment with climate targets. Using naphtha steam cracking (∼1.0 kg CO_2‑eq_·t MPW^–1^) as the 1990 best available technology (BAT) baseline, the EU targets translate to process-specific thresholds of ∼ 0.45 kg CO_2‑eq_·t MPW^–1^ by 2030 (55% reduction) and ∼ 0.1 kg CO_2‑eq_·t MPW^–1^ by 2040 (90% reduction).

Within this framing, transport-related emissions become a critical determinant of whether these systems (decentralised and centralised) remain compliant with EU-aligned emission constraints. Scope 3 emissions include MPW transport to the pyrolysis site (Location 1), which applies to both centralised and decentralised configurations, and PY-OIL transport to downstream steam crackers (Location 2), relevant only for the decentralised configuration where the pyrolyser and CST are spatially separated. The availability and proximity of downstream crackers therefore directly influence the GHG footprint of decentralised systems, potentially determining whether the system meets, the EU reduction targets.

#### 3.3.1 MPW Transport: Decentralised vs Centralised Configuration

Figure 9 shows the sensitivity of cradle-to-gate emissions to the MPW transport distance for the fully electrified, wind-powered scenarios. In order to ensure comparability between layouts, PY-OIL transport in the decentralised case is held constant at 100 km and therefore contributes a fixed background burden; only MPW transport is varied. In both configurations, emissions increase linearly with distance, but the threshold distances at which EU climate-policy benchmarks are exceeded differ between decentralised and centralised layouts.

**Figure 9:**
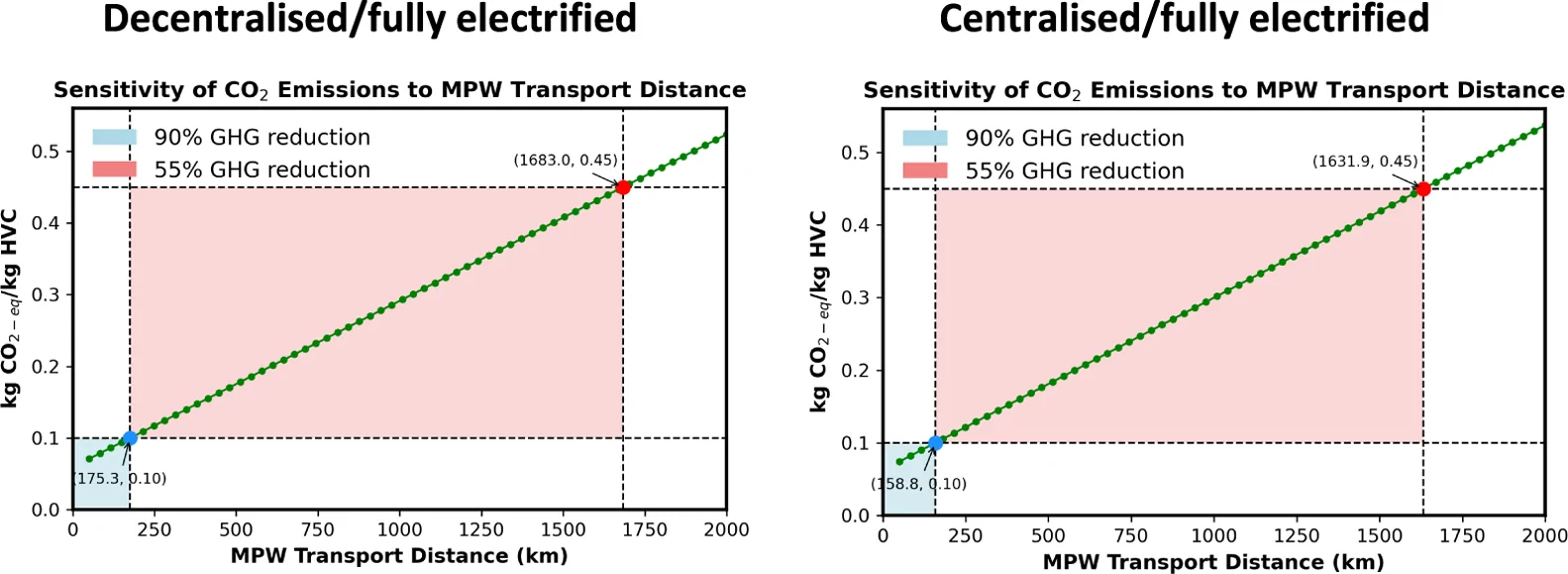
Sensitivity of CO_2‑eq_ emissions kg HVC^–1^ to MPW transport distance for decentralised (left) and centralised (right) configurations under fully electrified, renewable-powered conditions. Shaded areas indicate compliance with EU climate policy thresholds: blue = 90% reduction from BAT (0.10 kg CO_2‑eq_·kg HVC^–1^, 2040 target), red = 55% reduction (0.45 kg CO_2‑eq_·kg HVC^–1^, 2030 target). Decentralised systems reach the 90% reduction boundary at MPW distances of ∼175 km compared to centralised systems (∼159 km), while the 55% threshold is exceeded at ∼1683 km and ∼ 1632 km, respectively.

In the decentralised configuration (Figure 9-left), the 90%-reduction target for 2040 (0.10 kg CO_2‑eq_·kg HVC^–1^) is reached at approximately 175 km of MPW transport, while the 55%-reduction target for 2030 (0.45 kg CO_2‑eq_·kg HVC^–1^) is exceeded only beyond 1683 km. In the centralised system (Figure 9-right), the crossover points occur at ∼159 km (2040) and ∼ 1632 km (2030), respectively. The very similar slopes and the small differences in threshold distances indicate that, once the process is fully electrified and powered by renewable electricity, residual emissions are governed largely by MPW logistics rather than by the process configuration (i.e., decentralised or centralised). Although the centralised configuration avoids PY-OIL transport and achieves slightly higher olefin yields, these advantages do not substantially affect transport sensitivity under fully renewable conditions.

Figure 10 provides further insight by decomposing the contributions of individual emission sources as a function of MPW transport distance. At short distances (<200 km), electricity-related upstream emissions constitute the largest share of total CO_2‑eq_ emissions, reflecting the absence of direct process emissions in the fully electrified system and the comparatively small contribution from MPW transport. As MPW distance increases, transport-related emissions rise linearly and overtake electricity as the dominant contributor at ∼ 250 km. Beyond ∼ 500 km, MPW transport alone accounts for more than 80% of total cradle-to-gate emissions, while all other contributions, including PY-OIL transport in the decentralised configuration, remain nearly constant and collectively represent only a minor fraction of the total.

**Figure 10:**
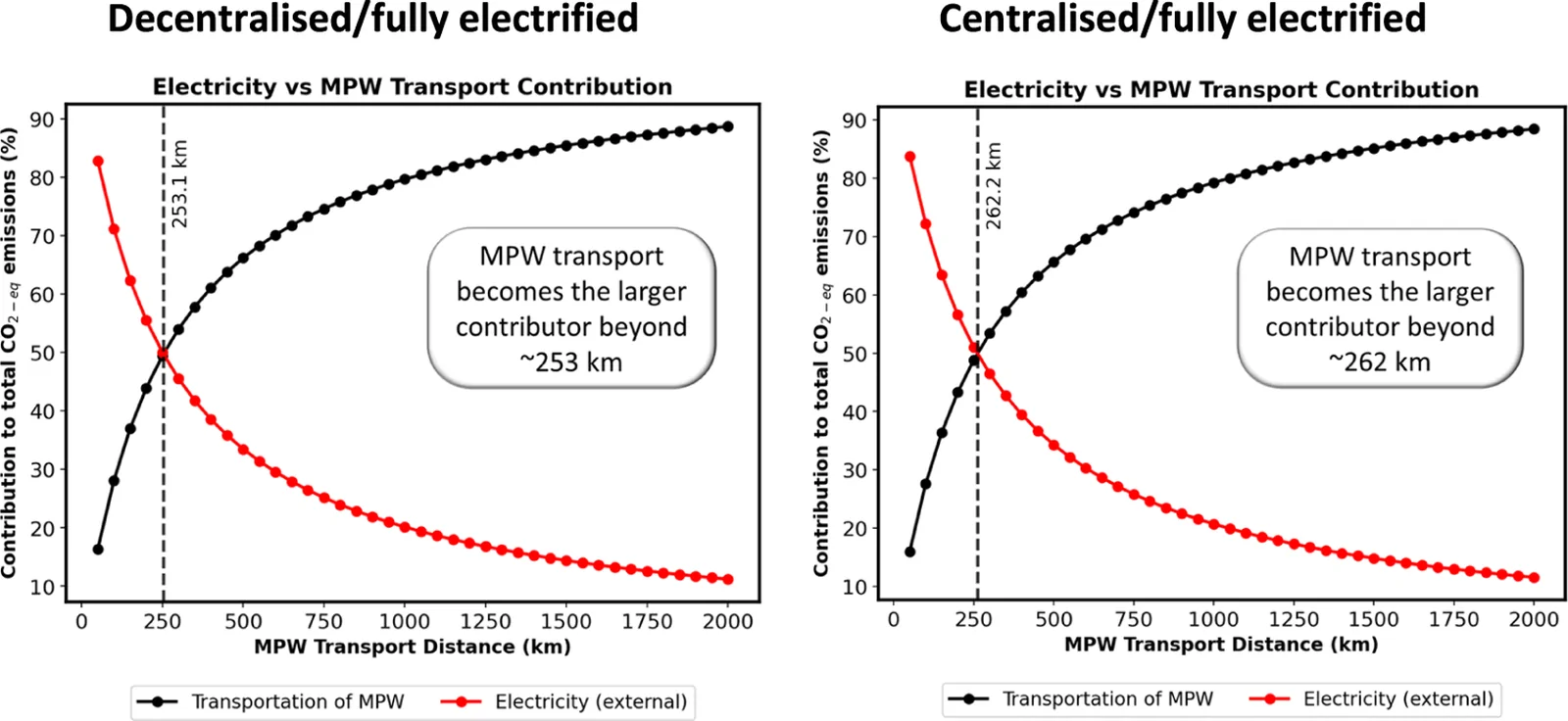
Contribution of each source to total CO_2‑eq_ emissions versus MPW transport distance for decentralised (left) and centralised (right) configurations under fully electrified, renewable-powered conditions. Electricity dominates at low distances, but MPW transport becomes the largest contributor beyond ∼ 250 km, determining whether systems remain within EU-aligned emission levels. For clarity, only the contributions of external electricity consumption and MPW transportation are highlighted, as all other sources exhibited negligible contributions across the evaluated transport distance range.

Overall, the 2030 target remains largely unaffected by MPW transport distance, since exceeding the 0.45 kg CO_2‑eq_ (kg HVC)^−1^ threshold would require transport distances well beyond realistic European collection ranges. By contrast, the 2040 target becomes strongly transport-dependent. Beyond roughly 200 km, logistics dominate the total emissions and the differences between decentralised and centralised layouts become marginal. As a result, both configurations exceed the 2040 threshold at near distances (∼159–175 km), showing that long-term decarbonisation target becomes increasingly constrained by feasible MPW sourcing radii rather than by the process configuration itself. This finding is consistent with recent supply chain optimization studies on thermochemical plastic waste upcycling. Ma et al.^74^ and Erickson et al.^75^ showed that thermochemical upcycling infrastructures involving collection, sorting, pyrolysis, steam cracking, and polymerization must be designed around geographically distributed waste availability, transportation requirements, and facility siting. Similarly, Badejo et al.^76^ demonstrated that transportation mode, facility deployment, and supply chain configuration strongly influence both emissions and economic performance, while electrification of transportation fleets reduced GHG emissions by ∼ 23% in their case study. The authors further showed that meeting Paris Agreement-aligned 2050 decarbonisation targets requires simultaneous optimization of transportation strategy, facility deployment, and technology selection under carbon-policy constraints, and concluded that current carbon-tax levels alone are insufficient to achieve these targets without broader deployment of recycling and upcycling infrastructure. Therefore, the present results support the broader conclusion that, under deeply electrified low-carbon operation, logistics and regional feedstock sourcing become the dominant constraints for further GHG reduction. In other words, the limiting factor for achieving ∼ 0.10 kg CO_2‑eq_·kg HVC^–1^ is not technology, but geography.

#### 3.3.2 PY-OIL Transport: Decentralised Configuration

The second transport stream, applied only in the case of the decentralised configuration, is PY-OIL, which is shipped from the pyrolysis site to the CST. A key distinction between MPW and PY-OIL transport is the disparity in their carbon intensities. Transporting MPW by truck emits 1.006 kg CO_2‑eq_·km^–1^,^77^ whereas PY-OIL marine transport emits only ∼ 0.0213 kg CO_2‑eq_·km^–1^,^78^ about 47 times lower, based on actual transported masses and emission factors. Consequently, avoiding just 1 km of MPW transport permits approximately 47 km of PY-OIL shipping without increasing total emissions.

The transport sensitivity cases (Figure 11) show that the system meets the 0.10 kg CO_2‑eq_·kg HVC^–1^ EU 2040 target across the evaluated MPW and PY-OIL transport distances. The assumed MPW trucking distances (120–175 km) align with typical European mixed-waste logistics, where collection-to-treatment distances of 100–250 km are commonly reported.^79^ Even when PY-OIL shipping extends to ∼2714 km, total transport emissions remain below the EU threshold.

**Figure 11:**
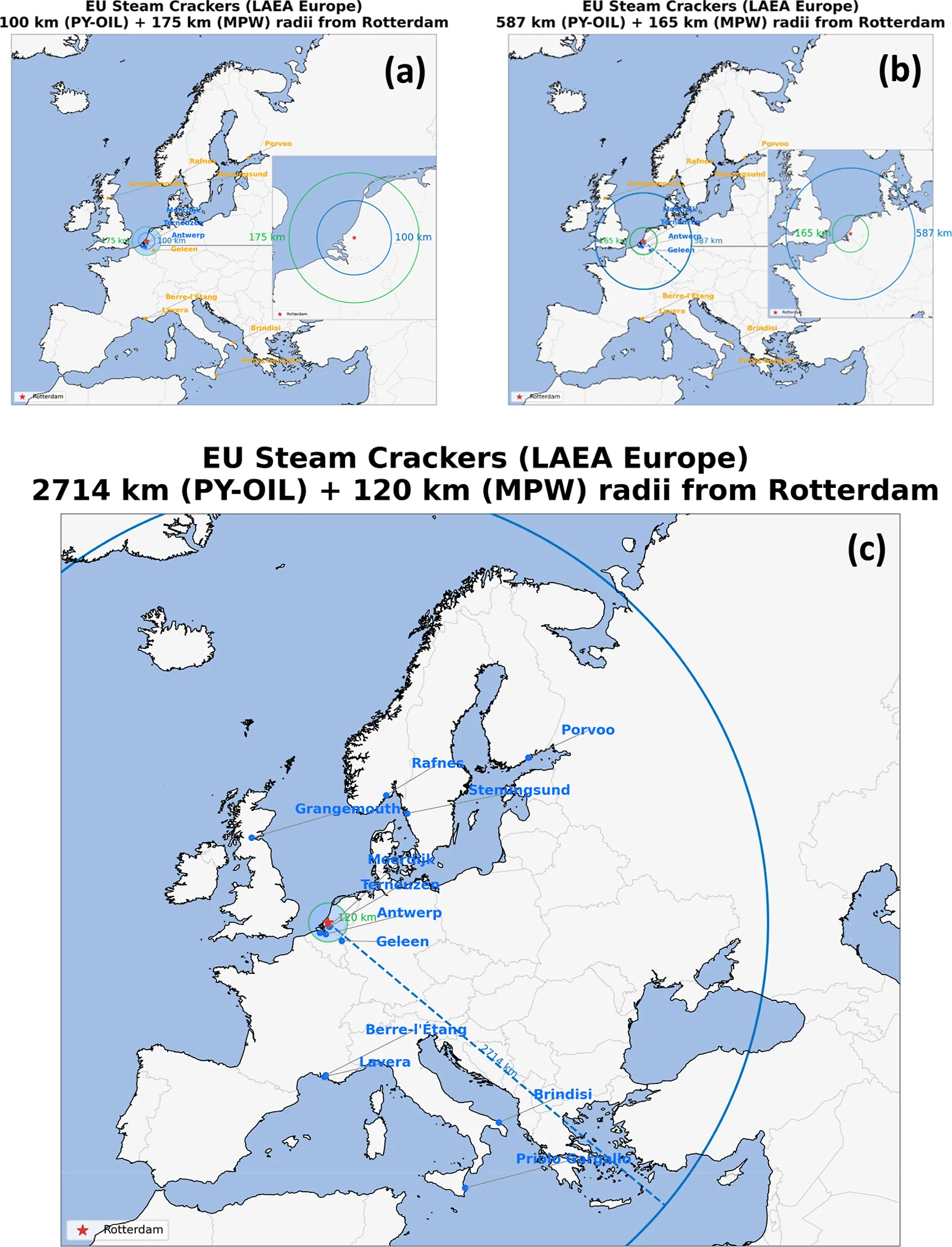
Sensitivity of CO_2‑eq_·kg HVC^–1^ to MPW and PY-OIL transport distances in the decentralised, fully electrified configuration. Three representative combinations of MPW and PY-OIL transport distances are illustrated using separate maps: (a) 100 km PY-OIL and 175 km MPW (top-left), (b) 587 km PY-OIL and 165 km MPW (top-right), and (c) 2714 km PY-OIL and 120 km MPW (bottom). Green circles indicate MPW transport radii, while blue circles indicate PY-OIL transport radii. Blue pins denote EU steam crackers located within each PY-OIL radius from Rotterdam, while orange pins lie outside.

The results highlight that MPW transport, not PY-OIL shipping, is the dominant driver of Scope 3 emissions, while PY-OIL logistics remain flexible even under stringent reduction targets.

## 4 Benchmarking

Section 4 presents the benchmarking analysis using two functional units (i.e., t MPW^–1^ and kg HVC^–1^), corresponding to the waste-management and product-oriented comparisons, respectively.

### 4.1 Per Tonne MPW: Waste Treatment Benchmarking

Figure 12 compares the carbon footprint of CHP-powered and fully electrified process configurations (grid and wind electricity) against traditional MPW treatment routes, including incineration, open burning, and sanitary landfill.

**Figure 12:**
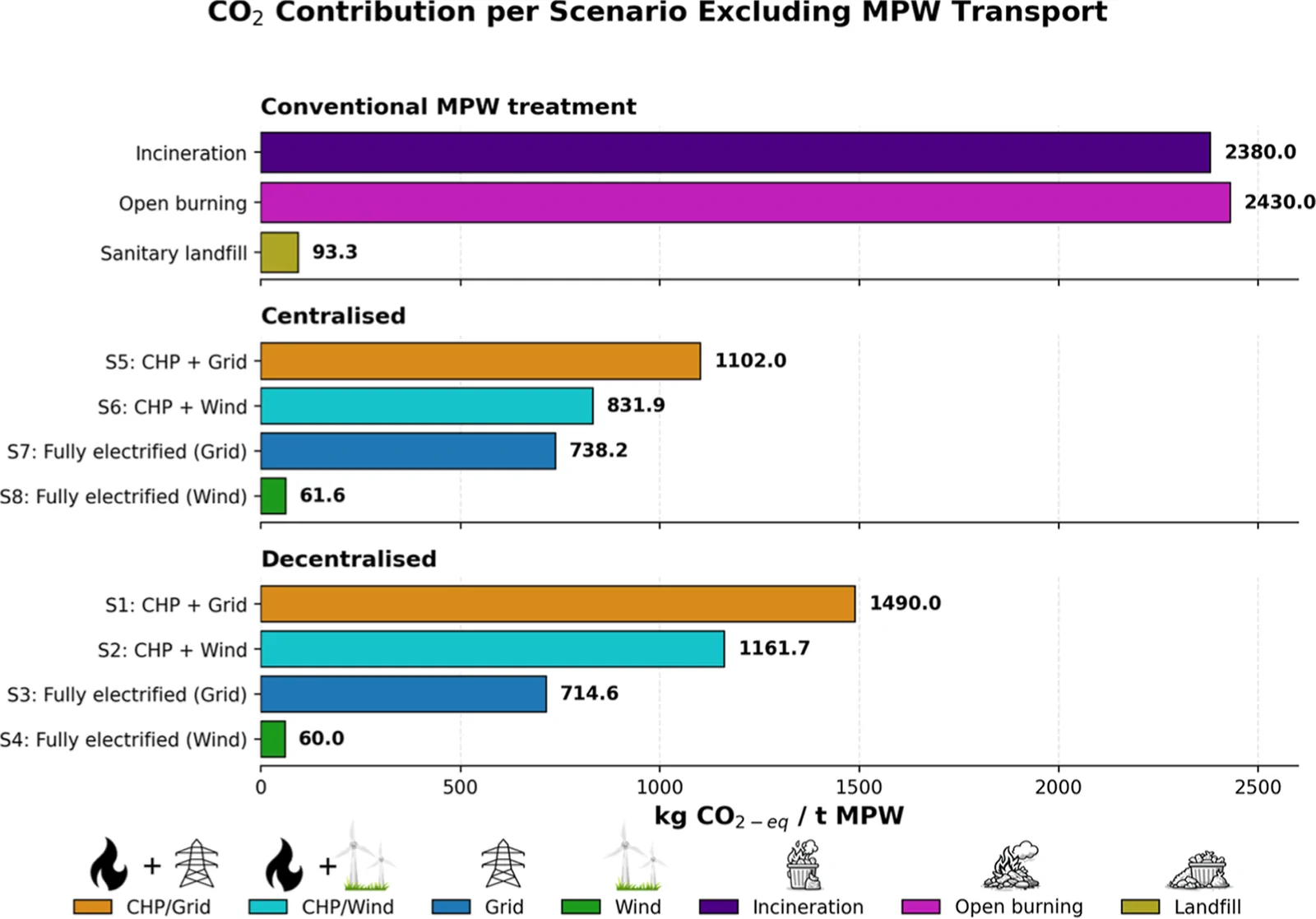
Carbon footprint benchmarking of different MPW treatment technologies. Municipal incineration of waste plastic mixture: controlled combustion of waste in the incineration plant with flue gas cleaning. Open burning of waste plastic mixture: uncontrolled burning, without flue gas treatment. Sanitary (controlled) landfill of waste plastic mixture.

Decentralised CHP-powered (grid) results in ∼1490 kg CO_2‑eq_·t MPW^–1^, substantially lower than incineration and open burning (2380–2430 kg CO_2‑eq_). Full electrification significantly improves performance: under wind electricity, emissions decrease to 60 kg CO_2‑eq_·t MPW^–1^, corresponding to a ∼ 96% reduction. The centralised fully electrified (wind) scenario yields ∼62 kg CO_2‑eq_·t MPW^–1^, while its grid-based counterpart remains comparable to the decentralised case (732.8 vs 714.6 kg CO_2‑eq_·t MPW^–1^.^9^

For consistency with conventional treatment options, upstream collection and transport of MPW is excluded from all cases, as is transport to Location 1 (Figure 12).

### 4.2 Per Tonne HVC: Benchmarking HVC Production Pathways

Figure 13 compares the carbon intensities of decentralised and centralised configurations across increasing electrification levels.

**Figure 13:**
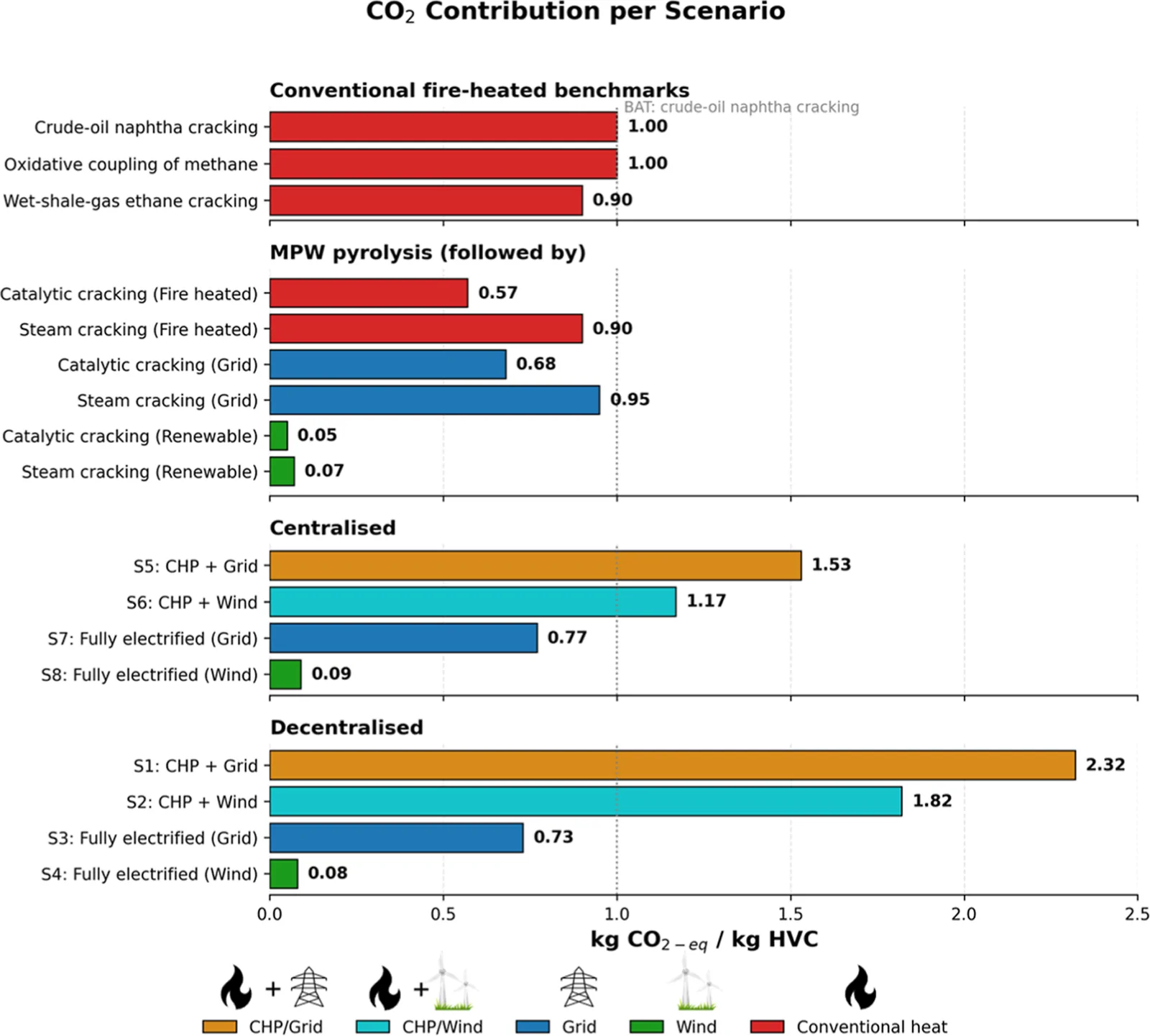
Carbon footprint benchmarking of different HVC Production Pathways.

In CHP-powered cases, emissions are dominated by combustion of DEC-OIL, reaching 2.32 kg CO_2‑eq_·kg HVC^–1^ for the decentralised and 1.53 kg CO_2‑eq_·kg HVC^–1^ for the centralised setup. Switching to wind electricity in these partially electrified systems lowers emissions to 1.82 CO_2‑eq_·kg HVC^–1^ and 1.17 CO_2‑eq_·kg HVC^–1^, respectively, due to reduced dependence on fossil-based power.

Full electrification further cuts emissions: with grid electricity, both configurations exhibit similar values of 0.73–0.77 CO_2‑eq_·kg HVC^–1^, whereas under renewable (wind) electricity, emissions decrease sharply to 0.08–0.09 CO_2‑eq_·kg HVC^–1^ for both the decentralised and centralised layouts.

For comparison, conventional fire-heated pyrolysis–steam-cracking systems emit approximately 0.90–0.95 CO_2‑eq_·kg HVC^–1^, whereas catalytic upgrading routes achieve 0.57–0.68 CO_2‑eq_·kg HVC^–1^. Benchmark technologies such as naphtha and ethane steam cracking or Oxidative Coupling of Methane (OCM) typically emit around 0.9–1.0 CO_2‑eq_ (kg HVC)^−1^ when expressed on an LCA-normalised, cradle-to-gate basis.^9^ These values are consistent with the definition of HVCs used throughout this study (ethylene, propylene, benzene, butadiene, hydrogen) and align with the product basket used in recent reviews (e.g., Theofanidis et al.).^9^ They also include indirect energy inputs such as upstream fuel production, electricity generation, and utility supply, ensuring methodological comparability with the present analysis.

Overall, full electrification and renewable power sourcing reduce the GHG intensity of mixed plastic waste upgrading by ∼ 90% relative to conventional naphtha cracking (BAT), aligning the process with the EU 2040 climate-neutrality target (∼0.1 kg CO_2‑eq_·kg HVC^–1^).

Table 9 summarizes the key differences in carbon footprint between decentralised and centralised process configurations across multiple functional units, highlighting the main contributors to each observed variation.

**Table 9:** Carbon Footprint Qualitative Differences Between Decentralised and Centralised configurations

aspect	general outcome	decentralised	centralised
electrification& energy source	electrification eliminates combustion-related emissions and enables large GHG reductions, especially with renewable electricity.	same trend.	same trend.
PYRO-GAS	internal reuse prevents flaring and supports higher HVC yields.	reused only in electrified cases (symbiosis).	always reused as CST feed.
DEC-OIL	used as CHP fuel in CHP-powered cases; credited as byproduct otherwise.	smaller recovered quantity.	larger recovery due to higher CST throughput.
utilities	compression and refrigeration dominate electricity demand in CST; heat recovery limits hot-utility use.	lower overall duty.	higher due to full PYRO-GAS processing.
transport (Scope 3)	MPW transport is a key sensitivity factor.	MPW + PY-OIL transport included.	only MPW transport considered.

Across both configurations, the carbon footprint is mainly governed by electrification, which inherently avoids combustion, and the carbon intensity of the electricity source, while differences between decentralised and centralised layouts have a secondary influence.

## 5 Conclusions

This study presents the conceptual process design and life-cycle greenhouse gas (GHG) analysis for converting mixed plastic waste (MPW) into high-purity light olefins. It aims to quantify the associated GHG emissions and identify the key environmental drivers of the process under both decentralised and centralised configurations. Across all scenarios, two overarching findings emerge clearly from the results.

First, electrification and the elimination of combustion-related heat and power generation are the dominant determinants of GHG performance. CHP-powered configurations exhibit the highest emissions due to direct CO_2_ release from decanter-oil combustion and, in decentralised layouts, flaring of PYRO-GAS. These scenarios result in cradle-to-gate emissions of approximately 1185–1514 kg CO_2‑eq_·t MPW^–1^ for decentralised systems and 855–1126 kg CO_2‑eq_·t MPW^–1^ for centralised ones. Transitioning to fully electrified operation almost entirely eliminates these direct combustion emissions, reducing impacts to ∼ 738–762 kg CO_2‑eq_·t MPW^–1^ when supplied by the EU grid and to ∼ 84–85 kg CO_2‑eq_·t MPW^–1^ when powered by renewable (onshore wind) electricity. These reductions confirm that electrification is a prerequisite for deep decarbonisation of MPW-to-olefins systems, while the carbon intensity of electricity supply ultimately determines whether climate-neutral performance can be achieved.

Second, process configuration primarily affects material utilization, energy distribution, and logistics rather than the fundamental decarbonisation potential. Centralised layouts benefit from colocation of pyrolysis and the CST, which enables direct utilization of the hot PYRO-GAS stream within the downstream processing section. This avoids flaring and allows PYRO-GAS to be valorised as part of the hydrocarbon feed to the CST, increasing overall hydrocarbon throughput and resulting in higher olefin yields (38.0 wt % versus 33.8 wt % in decentralised configurations). In addition, centralisation eliminates PY-OIL transportation required in decentralised designs.

By contrast, decentralised configurations cannot benefit from hot PYRO-GAS integration, as the pyrolysis and CST units are spatially separated. In these cases, only the cracked effluent generated in the RDR from the preheated HC/steam mixture is routed to the CST, while PYRO-GAS is either flared in CHP-powered scenarios or assumed to be valorised externally through industrial symbiosis under fully electrified operation. As a result, decentralised layouts handle a lower hydrocarbon throughput in the CST, leading to lower vapor loads and reduced refrigeration and compression duties per tonne of MPW processed, but they incur additional logistical requirements associated with PY-OIL transport.

These configuration-dependent effects are most pronounced under combustion-based operation but become secondary under full electrification. In CHP-powered cases, the higher cracked-gas throughput enabled by PYRO-GAS integration in centralised layouts translates into higher absolute CST heating and compression duties, reflected in increased hot-utility and mechanical-energy demand. Under fully electrified operation, although the total energy demand per tonne of MPW is higher for the centralised configuration, the increased olefin yields more than compensate for this effect. When normalised to total olefin production, the resulting energy intensity decreases from approximately 24.1 MJ·kg_OLEF_^–1^ in the decentralised system to 22.2 MJ·kg_OLEF_^–1^ in the centralised system. Consequently, while centralisation retains a material-efficiency advantage, the overall decarbonisation outcome is governed primarily by electrification and electricity sourcing rather than by configuration alone.

The sensitivity analysis further demonstrates that, under low-carbon operation, Scope 3 emissions, particularly MPW transport, become the dominant constraint. In fully electrified, renewable-powered scenarios, MPW transport accounts for the majority of residual emissions, and the EU 2040 climate target (∼0.10 kg CO_2‑eq_·kg HVC^–1^, corresponding to a 90% reduction relative to BAT) is met only when MPW sourcing distances remain below approximately 160–180 km. Beyond this range, transport emissions dominate the cradle-to-gate footprint irrespective of whether the system is decentralised or centralised. By contrast, PY-OIL transport in decentralised configurations has a comparatively minor impact due to its much lower emission intensity per kilometer, confirming that MPW logistics, rather than interplant oil transport, set the primary geographical limits for deep decarbonisation.

Overall, the results demonstrate that electrified MPW-to-olefins pathways can achieve very low cradle-to-gate GHG emissions when supported by low-carbon electricity, with carbon intensities of approximately 0.08–0.09 kg CO_2‑eq_·kg HVC^–1^ in fully electrified, renewable-powered configurations. These values are substantially lower than those reported for conventional naphtha steam cracking (∼1.0 kg CO_2‑eq_·kg HVC^–1^) and advanced catalytic upgrading routes (∼0.57–0.68 kg CO_2‑eq_·kg HVC^–1^). While electrification and electricity sourcing dominate environmental performance, process configuration influences material efficiency, absolute utility demand, and sensitivity to transport-related emissions. Together, these findings provide a robust, system-level foundation for the design of circular, low-carbon olefin production routes based on mixed plastic waste and highlight the critical role of spatial planning, logistics, and infrastructure integration in achieving long-term climate targets.

## Supplementary Material





## Notes

Life-cycle inventory data sources and collection; equations implemented in the developed process-simulation code, including thermodynamic property methods and unit-operation models; heat-integration data and technical results; system boundaries and process-level mass and energy balances for the eight scenarios; heat-exchanger networks for the decentralised and centralised configurations; process overview and mass balances; refrigeration, steam, and cooling-water utility systems; detailed life-cycle greenhouse gas emission results; background data sets retrieved from the ecoinvent database (PDF)
(XLSX)

## Abbreviations

Abbreviation: Description
CHP: combined heat and power
CST: compression and separation train
DEC-OIL: decanter oil
ETH: ethylene
GHG: greenhouse gas
HDPE: high-density polyethylene
HVC: high-value chemical
LCI: life-cycle inventory
LCIA: life-cycle impact assessment
MAPD: methylacetylene–propadiene
MFC: mass flow controller/splitter
MPW: mixed plastic waste
PREOS: peng–robinson equation of state
PSA: pressure swing adsorption
PY-OIL: pyrolysis oil
PYRO-GAS: pyrolysis gas
RDR: RotoDynamic Reactor
S1–8: scenarios 1–8
SI: Supporting Information
t: tonne (10³ kg)
TEA: techno-economic analysis
TLE: transfer line exchanger
