# Supplementary material for: Process Design and Greenhouse Gas Analysis of an Electrified Mixed Plastic Waste-to-Olefins Value Chain
Source: Ind Eng Chem Res. 2026 Aug 16;65(33):17875–96. doi: 10.1021/acs.iecr.6c01225 (PMC13528584; doi:10.1021/acs.iecr.6c01225)
Supplement: Supplementary file 1 [file ie6c01225_si_001.pdf]

Process Design and Greenhouse Gas Analysis of an Electrified Mixed Plastic Waste-to-Olefins Value Chain

Amvrosios G. Georgiadis [a], Vasileia-Loukia Yfanti [a], Ismaël Amghizar [b], David J. Brown [b], Azd Zayoud [c,d], Guy B. Marin [b], Kevin Van Geem [c], Evangelos Delikonstantis [a,*], Stavros-Alexandros Theofanidis [a,*]

[a] *AristEng S.à r.l., 20, Avenue Pasteur, L-2310 Luxembourg City, Luxembourg*
[b] *AVGI bv, Technologiepark-Zwijnaarde 122, 9052 Ghent, Belgium*
[c] *Laboratory for Chemical Technology, Ghent University, 9052 Ghent, Belgium*
[d] *Pryme NV, 3065 Rotterdam, The Netherlands*

*Evangelos Delikonstantis	Email: *evangelos.delikonstantis@aristeng.lu*
*Stavros-Alexandros Theofanidis	Email: *stavros.theofanidis@aristeng.lu*

Supporting Information

I. LCI (Life-Cycle Inventory): Data Sources & Collection

Detailed PYRO-GAS composition used as input to the life-cycle assessment.

Table S1: PYRO-GAS composition (total 150 kg·t MPW⁻¹).

Component	450 °C, 0 ppm (wt.%)	Component	450 °C, 0 ppm (wt.%)
CH_4	2.1	cis-C_4H_8	12.7
C_2H_6	6.0	$CH_3C{\equiv}CH$	1.1
C_2H_4	5.4	1,3-C_4H_6	0.1
C_3H_8	5.2	n-C_5H_{12}	2.7
C_3H_6	26.7	i-C_5H_{12}	4.9
i-C_4H_{10}	1.5	trans-C_5H_{10}	2.4
C_2H_2	0.2	1-C_5H_{10}	1.3
$CH_2{=}C{=}CH_2$	0.5	cis-C_5H_{10}	2.4
n-C_4H_{10}	4.5	C_6^+	1.6
trans-C_4H_8	18.0	CO_2	0.0
1-C_4H_8	0.6	H_2	0.0
i-C_4H_8	0.1	CO	0.0

II. Equations for Developed Code

This section contains the equations used in the in-house developed code, implemented in a Python environment. To improve readability, variable/parameter definitions are provided immediately below each equation, making it easier for readers to understand without needing to navigate back and forth. All units are expressed in SI. For the distillation section, only the equations are presented without including units in the variable/parameter definitions. This decision is made to streamline the presentation and focus on the mathematical framework, as the distillation equations are typically applied in a unit-independent manner within the broader context of the model.

Property Method – PREOS[1]

Equation 1: Cubic equation of state, PREOS.

$$P = \frac{R \cdot T}{v - b} - \frac{\text{alpha} \cdot \alpha}{v \cdot (v + b) + b \cdot (v - b)}$$

Where:

- P: Pressure (Pa).
- T: Temperature (K).
- v: Molar volume ($m^3 \cdot mol^{-1}$).
- R: Universal gas constant ($Pa \cdot m^3 \cdot mol^{-1} \cdot K^{-1}$)
- α: Attraction parameter ($Pa \cdot m^6 \cdot mol^{-2}$).
- b: Repulsion parameter ($m^3 \cdot mol^{-1}$).
- alpha: Temperature-dependent correction factor (unitless).

Equation 2: Temperature dependent correction factor, PREOS.

$$\mathrm{alpha} = \left(1 + \kappa \cdot \left(1 - \sqrt{T_r}\right)\right)^2$$

Where:

- alpha: Temperature-dependent correction factor (unitless).
- $\kappa = 0.37464 + 1.5422\omega - 0.26992\omega^2$: Constant based on the acentric factor ω (unitless).
- $T_r = \frac{T}{T_c}$: Reduced temperature (unitless).
- T: Temperature (K).
- T_c: Critical temperature (K).

Equation 3: Attraction parameter, PREOS.

$$\alpha = 0.457235 \cdot \frac{R^2 \cdot T_c^2}{P_c}$$

Where:

- α: Attraction parameter ($Pa \cdot m^6 \cdot mol^{-2}$).
- R: Universal gas constant ($Pa \cdot m^3 \cdot mol^{-1} \cdot K^{-1}$).
- T_c: Critical temperature (K).
- P_c: Critical pressure (Pa).

Equation 4: Repulsion parameter, PREOS.

$$b = 0.0777961 \frac{R \cdot T_c}{P_c}$$

Where:

- b: Repulsion parameter ($m^3 \cdot mol^{-1}$).
- R: Universal gas constant ($Pa \cdot m^3 \cdot mol^{-1} \cdot K^{-1}$).
- T_c: Critical temperature (K).
- P_c: Critical pressure (Pa).

Equation 5: Compressibility factor, PREOS.

$$Z^3 - (1 - B) \cdot Z^2 + (A - 2B - 3B^2) \cdot Z - (A \cdot B - B^2 - B^3) = 0$$

Where:

- Z: Compressibility factor (unitless).
- $A = \frac{\mathrm{alpha} \cdot \alpha \cdot P}{R^2 \cdot T^2}$: Reduced parameter (unitless).
- $B = \frac{b \cdot P}{R \cdot T}$: Reduced repulsion parameter (unitless).

Equation 6: Fugacity coefficient, PREOS.

$$\ln\varphi = Z - 1 - \ln(Z - B) - \frac{A}{\sqrt{8}B} \cdot \ln\left(\frac{Z + \left(1 + \sqrt{2}\right) \cdot B}{Z + \left(1 - \sqrt{2}\right) \cdot B}\right)$$

Where:

- φ: Fugacity coefficient (unitless).
- Z: Compressibility factor (unitless).

- A: Reduced parameter (unitless).
- B: Reduced repulsion parameter (unitless).

Equation 7: Mixture properties, PREOS.

$$\alpha_{mix} = \sum_{i}\sum_{j} x_i x_j \sqrt{\alpha_i \alpha_j}(1 - k_{ij})$$

Where:

- α_{mix}: Mixture attraction parameter (Pa·m⁶·mol⁻²).
- x_i, x_j: Mole fractions (unitless).
- α_i, α_j: Attraction parameters for components (Pa·m⁶·mol⁻²).
- k_{ij} : binary interaction parameter (unitless).

Equation 8: Repulsion parameter for mixtures, PREOS.

$$b_{mix} = x_1 \cdot b_1 + x_2 \cdot b_2$$

Where:

- b_{mix}: Mixture repulsion parameter (m³·mol⁻¹).
- x_1, x_2: Mole fractions of components 1 and 2 (unitless).
- b_1, b_2: Attraction parameters for components 1 and 2 (m³·mol⁻¹).

Equation 9: Density calculation, PREOS.

$$\rho = \frac{P}{Z \cdot R \cdot T}$$

Where:

- ρ: Density (mol·m⁻³).
- P: Pressure (Pa).
- Z: Compressibility factor (unitless).
- R: Universal gas constant (Pa·m³·mol⁻¹·K⁻¹).
- T: Temperature (K).

Property Method – IAPWS-95[2]

Equation 10: Helmholtz energy formulation, IAPWS-95.

$$\varphi(\tau, \delta) = \frac{f}{R \cdot T} = \varphi^0 \cdot (\tau, \delta) + \varphi^r \cdot (\tau, \delta)$$

Where:

- φ: Dimensionless Helmholtz free energy (unitless).
- f: Helmholtz free energy (J·mol⁻¹).
- R: Universal gas constant (J·mol⁻¹·K⁻¹).
- T: Temperature (K).
- $\tau = T_c/T$: Inverse reduced temperature (unitless).
- $\delta = \rho/\rho_c$: Reduced density (unitless).
- φ^0: Ideal-gas contribution (unitless).
- φ^r: Residual contribution (unitless).

Equation 11: Pressure, IAPWS-95.

$$P = \rho \cdot R \cdot T \cdot (1 + \delta \cdot \varphi_\delta^r)$$

Where:

- P: Pressure (Pa).
- ρ: Density ($mol \cdot m^{-3}$).
- R: Universal gas constant ($J \cdot mol^{-1} \cdot K^{-1}$).
- T: Temperature (K).
- δ: Reduced density (unitless).
- $\varphi_\delta^r = \partial\varphi^r / \partial\delta$: First derivative of the residual term with respect to density.

Equation 12: Density (implicit), IAPWS-95.

Density is obtained by iterative solution of Equation 13.

$$P = \rho \cdot R \cdot T \cdot [1 + \delta \cdot \varphi_\delta^r(\tau, \delta)]$$

Where:

- ρ: Density is obtained by iterative solution of the above non-linear equation.

Equation 14: Entropy, IAPWS-95.

$$s = R \cdot (\tau \cdot \varphi_\tau - \varphi)$$

Where:

- s: Molar entropy ($J \cdot mol^{-1} \cdot K^{-1}$).
- R: Universal gas constant ($J \cdot mol^{-1} \cdot K^{-1}$).
- $\tau = T_c / T$: Inverse reduced temperature (unitless).
- $\varphi_\tau = \partial\varphi / \partial\tau$
- φ: Dimensionless Helmholtz free energy (unitless).

Equation 15: Enthalpy, IAPWS-95.

$$h = R \cdot T \cdot (1 + \tau \cdot \varphi_\tau + \delta \cdot \varphi_\delta^r)$$

Where:

- h: Molar enthalpy ($J \cdot mol^{-1}$).
- R: Universal gas constant ($J \cdot mol^{-1} \cdot K^{-1}$).
- T: Temperature (K).
- $\tau = T_c / T$: Inverse reduced temperature (unitless).
- $\varphi_\tau = \partial\varphi / \partial\tau$
- δ: Reduced density (unitless).
- $\varphi_\delta^r = \partial\varphi^r / \partial\delta$: First derivative of the residual term with respect to density.

Equation 16: Internal energy, IAPWS-95.

$$u = R \cdot T \cdot (\tau \cdot \varphi_\tau)$$

Where:

- u: Internal energy ($J \cdot mol^{-1}$).
- R: Universal gas constant ($J \cdot mol^{-1} \cdot K^{-1}$).

- T: Temperature (K).
- $\tau = T_c/T$: Inverse reduced temperature (unitless).
- $\varphi_\tau = \partial\varphi/\partial\tau$

Equation 17: Gibbs free energy, IAPWS-95.

$$g = h - T \cdot s$$

Where:

- g: Molar Gibbs energy (J·mol⁻¹).
- h: Molar enthalpy (J·mol⁻¹).
- T: Temperature (K).
- s: Molar entropy (J·mol⁻¹·K⁻¹).

Equation 18: Isochoric heat capacity, IAPWS-95.

$$C_v = -R \cdot \tau^2 \cdot \varphi_{\tau\tau}$$

Where:

- C_v: Heat capacity at constant volume (J·mol⁻¹·K⁻¹).
- R: Universal gas constant (J·mol⁻¹·K⁻¹).
- $\tau = T_c/T$: Inverse reduced temperature (unitless).
- $\varphi_{\tau\tau} = \partial^2\varphi/\partial\tau^2$: Second derivative with respect to τ.

Equation 19: Isobaric heat capacity, IAPWS-95.

$$C_p = C_v + R \cdot \frac{[1 + \delta \cdot \varphi^r_\delta - \delta \cdot \tau \cdot \varphi^r_{\delta\tau}]^2}{1 - \delta^2 \cdot \varphi^r_{\delta\delta}}$$

Where:

- C_p: Heat capacity at constant pressure (J·mol⁻¹·K⁻¹).
- C_v: Heat capacity at constant volume (J·mol⁻¹·K⁻¹).
- R: Universal gas constant (J·mol⁻¹·K⁻¹).
- δ: Reduced density (unitless).
- $\varphi^r_{\delta\tau} = \partial^2\varphi^r/\partial\delta\,\partial\tau$: Mixed derivative of the residual term with respect to δ and τ.
- $\varphi^r_{\delta\delta} = \partial^2\varphi^r/\partial\delta^2$: Second derivative of the residual term with respect to δ.

Block equations[3]

Flash Separator

Equation 20: K-value for flash separator

$$K_i = \frac{\gamma_i P_i^{sat}}{\varphi_i P}$$

Where:

- K_i: Equilibrium ratio (K-value) for component i, indicating vapor-liquid partitioning.

- P_i^{sat}: Saturation pressure of component i at the system temperature T (Pa).
- P: Total system pressure (Pa).
- γ_i: activity coefficient (unitless).
- φ_i: vapor fugacity (unitless).

Equation 21: Rachford-Rice for flash separator.

$$0 = \sum_{i=1}^{N} Z_i \cdot \frac{K_i - 1}{1 + v \cdot (K_i - 1)}$$

Where:

- v: Vapor fraction in the mixture (unitless).
- Z_i: Overall molar fraction of component i in the feed stream (unitless).
- K_i: Equilibrium ratio for component i (unitless).
- N: Total number of components.

Equation 22: Vapor phase molar fraction for flash separator.

$$y_i = \frac{K_i \cdot Z_i}{1 + v \cdot (K_i - 1)}$$

Where:

- y_i: Molar fraction of component i in the vapor phase (unitless).
- K_i: Equilibrium ratio for component i.
- Z_i: Overall molar fraction of component i.
- v: Vapor fraction in the mixture (unitless).

Equation 23: Liquid phase molar fraction for flash separator.

$$x_i = \frac{Z_i}{1 + v \cdot (K_i - 1)}$$

Where:

- x_i: Molar fraction of component i in the liquid phase (unitless).
- Z_i: Overall molar fraction of component i.
- K_i: Equilibrium ratio for component i.
- v: Vapor fraction in the mixture (unitless).

Equation 24: Vapor flow rate for flash separator.

$$V = F \cdot v$$

Where:

- V: Molar flow rate of the vapor phase ($mol \cdot s^{-1}$).
- F: Total molar flow rate of the feed stream ($mol \cdot s^{-1}$).
- v: Vapor fraction in the mixture (unitless).

Equation 25: Liquid flow rate for flash separator.

$$L = F \cdot (1 - v)$$

Where:

- L: Molar flow rate of the liquid phase (mol·s⁻¹).
- F: Total molar flow rate of the feed stream (mol·s⁻¹).
- v: Vapor fraction in the mixture (unitless).

Equation 26: Mass fraction conversion for flash separator.

$$w_i = \frac{f_i \cdot M_i}{\sum_{j=1}^{N} f_j \cdot M_j}$$

Where:

- w_i: Mass fraction of component i in the vapor or liquid phase (unitless).
- f_j: Molar fraction of component i in either the vapor (y_i) or liquid (x_i) phase.
- M_j: Molar mass of component i (kg·mol⁻¹).
- N: Total number of components.

Equation 27: Vapor mass flow rate for flash separator.

$$\dot{m}_V = V \cdot \sum_{i=1}^{N} y_i \cdot M_i$$

Where:

- $\dot{m}_V$: Total mass flow rate of the vapor phase (kg·s⁻¹).
- V: Molar flow rate of the vapor phase (mol·s⁻¹).
- y_i: Molar fraction of component i in the vapor phase (unitless).
- M_i: Molar mass of component i (kg·mol⁻¹).
- N: Total number of components.

Equation 28: Liquid mass flow rate for flash separator.

$$\dot{m}_L = L \cdot \sum_{i=1}^{N} x_i \cdot M_i$$

Where:

- $\dot{m}_L$: Total mass flow rate of the liquid phase (kg·s⁻¹).
- L: Molar flow rate of the liquid phase (mol·s⁻¹).
- x_i: Molar fraction of component i in the liquid phase (unitless).
- M_i: Molar mass of component i (kg·mol⁻¹).
- N: Total number of components.

Equation 29: Total input mass flow rate for flash separator.

$$\dot{m}_{feed} = F \cdot \sum_{i=1}^{N} Z_i \cdot M_i$$

Where:

- $\dot{m}_{feed}$: Total mass flow rate of the feed stream (kg·s⁻¹).
- F: Total molar flow rate of the feed stream (mol·s⁻¹).
- Z_i: Overall molar fraction of component i.
- M_i: Molar mass of component i (kg·mol⁻¹).
- N: Total number of components.

Equation 30: Energy balance for flash separator.

$$F \cdot H_{feed} + Q = V \cdot H_{vapor} + L \cdot H_{liquid}$$

Where:

- F: Total molar flow rate of the feed stream (mol·s⁻¹).
- $H_{feed} = \sum_{i=1}^{N} Z_i H_i$: Molar enthalpy of the feed stream (J·mol⁻¹).
- Z_i: Overall molar fraction of component i.
- $H_{vapor} = \sum_{i=1}^{N} y_i \cdot H_i^V$: Molar enthalpy of the vapor stream (J·mol⁻¹)
- y_i: Molar fraction of component i in the vapor phase (unitless).
- H_i^V: Molar enthalpy of component i in vapor phase (J·mol⁻¹).
- $H_{liquid} = \sum_{i=1}^{N} x_i \cdot H_i^L$: Molar enthalpy of the liquid stream (J·mol⁻¹)
- x_i: Molar fraction of component i in the liquid phase (unitless).
- H_i^L: Molar enthalpy of component i in liquid phase.
- Q: For a heater $Q > 0$ (heat is added), for a cooler $Q < 0$ (heat is removed). For an adiabatic flash $Q = 0$.
- N: Total number of components.

Mixer

Equation 31: Total mass flow rate for mixer.

$$\dot{m}_{total} = \sum_{i=1}^{N} \dot{m}_i$$

Where:

- $\dot{m}_{total}$: Total mass flow rate (kg·s⁻¹).
- $\dot{m}_i$: Mass flow rate of stream i (kg·s⁻¹).
- N: Total number of streams.

Equation 32: Mass-flow weighted specific enthalpy of the mixed stream in the mixer.

$$h_{mixed} = \frac{\sum_{i=1}^{N} \dot{m}_i h_i}{\sum_{i=1}^{N} \dot{m}_i}$$

Where:

- h_{mixed}: Mixed specific enthalpy (J·kg⁻¹).
- $\dot{m}_i$: Mass flow rate of stream i (kg·s⁻¹).
- h_i: Specific enthalpy of inlet stream i at (T_i, P_i) (J·kg⁻¹).

- N: Total number of streams.

Equation 33: Mixed temperature for mixer.

$$T_{mixed} = f^{-1}(h_{mixed}, P_{mixed})$$

Where:

- T_{mixed}: Mixed temperature (K).
- P_{mixed}: Mixed pressure (Pa).
- f^{-1}: Inverse property relation giving T from (h, P) for the mixture used.

Equation 34: Mixture mass fraction for mixer.

$$z_i = \frac{\dot{m}_i}{\dot{m}_{total}}$$

Where:

- z_i: Mass fraction of fluid i in the mixture (unitless).
- $\dot{m}_i$: Mass flow rate of stream i ($kg \cdot s^{-1}$).
- $\dot{m}_{total}$: Total mass flow rate ($kg \cdot s^{-1}$).

Equation 35: Mixed pressure for mixer.

$$P_{mixed} = \min_{i=1}^{N} P_i$$

Where:

- P_{mixed}: Mixed pressure (Pa).
- P_i: Pressure of stream i.
- N: Total number of streams.

Isentropic Compressor

Equation 36: Isentropic head calculation for isentropic compressor.

$$H_{is} = \left(\frac{\gamma}{\gamma - 1}\right) \cdot z \cdot R_{specific} \cdot T_{in} \left(\left(\frac{P_{out}}{P_{in}}\right)^{\frac{\gamma-1}{\gamma}} - 1\right)$$

Where:

- H_{is}: Isentropic head ($J \cdot kg^{-1}$).
- γ: Heat capacity ratio (unitless).
- z: Compressibility factor (unitless).
- $R_{specific}$: Specific gas constant ($J \cdot kg^{-1} \cdot K^{-1}$).
- T_{in}: Inlet temperature (K).
- P_{out}: Outlet pressure (Pa).
- P_{in}: Inlet pressure (Pa).

Equation 37: Isentropic outlet temperature for isentropic compressor.

$$T_{out,isentropic} = T_{in} \cdot \left(\frac{P_{out}}{P_{in}}\right)^{\frac{\gamma-1}{\gamma}}$$

Where:

- $T_{out,isentropic}$: Isentropic outlet temperature (K).
- T_{in}: Inlet temperature (K).
- P_{out}: Outlet pressure (Pa).
- P_{in}: Inlet pressure (Pa).
- γ: Heat capacity ratio (unitless).

Equation 38: Actual outlet temperature considering efficiency for isentropic compressor.

$$T_{out,actual} = T_{in} + \frac{T_{out,isentropic} - T_{in}}{\eta}$$

Where:

- $T_{out,actual}$: Actual outlet temperature (K).
- T_{in}: Inlet temperature (K).
- $T_{out,isentropic}$: Isentropic outlet temperature (K).
- η: Compressor efficiency (unitless).

Equation 39: Actual power for isentropic compressor.

$$P_{actual} = \dot{m}_{total} \cdot \frac{H_{is}}{\eta}$$

Where:

- P_{actual}: Actual power ($J \cdot s^{-1}$).
- $\dot{m}_{total}$: Total mass flow rate ($kg \cdot s^{-1}$).
- H_{is}: Isentropic heat ($J \cdot kg^{-1}$).
- η: Compressor efficiency (unitless).

Isentropic turbine

Equation 40: Isentropic specific enthalpy at the outlet for isentropic turbine.

$$h_{out,isentropic} = f(P_{out}, s_{in})$$

Where:

- $h_{out,isentropic}$: Isentropic specific enthalpy at the outlet ($J \cdot kg^{-1}$).
- P_{out}: Outlet pressure (Pa).
- s_{in}: Inlet specific entropy($J \cdot kg^{-1} \cdot K^{-1}$).

Equation 41: Actual specific enthalpy for isentropic turbine.

$$h_{out,actual} = h_{in} - \eta \cdot \left(h_{in} - h_{out,isentropic}\right)$$

Where:

- $h_{out,actual}$: Actual specific enthalpy at the outlet (J·kg⁻¹).
- h_{in}: Inlet specific enthalpy (J·kg⁻¹).
- η: Turbine efficiency (unitless).
- $h_{out,isentropic}$: Outlet isentropic specific enthalpy (J·kg⁻¹).

Equation 42: Actual outlet temperature for isentropic turbine.

$$T_{out,actual} = f(P_{out}, h_{out,actual})$$

Where:

- $T_{out,actual}$: Actual outlet temperature (K).
- P_{out}: Outlet pressure (Pa).
- $h_{out,actual}$: Outlet actual specific enthalpy (J·kg⁻¹).

Equation 43: Enthalpy-based power output for isentropic turbine.

$$W = \dot{m}_{total} \cdot (h_{in} - h_{out,actual})$$

Where:

- W: Turbine power (J·s⁻¹).
- $\dot{m}_{total}$: Total mass flow rate (kg·s⁻¹).
- h_{in}: Inlet specific enthalpy (J·kg⁻¹).
- $h_{out,actual}$: Outlet actual specific enthalpy (J·kg⁻¹).

Pump

Equation 44: Mixture density for pump.

$$\rho_{mixture} = \left(\sum_{i}^{N} w_i/\rho_i\right)^{-1}$$

Where:

- $\rho_{mixture}$: Mixture density (kg·m⁻³)
- w_i: Mass fraction of component i (unitless).
- ρ_i: Density of component i (kg·m⁻³).
- N: Total number of components.

Equation 45: Mixture specific heat capacity for pump.

$$C_{p,mixture} = \sum_{i=1}^{N} x_i \cdot C_{p,i}$$

Where:

- $C_{p,mixture}$: Specific heat capacity of mixture (J·kg⁻¹·K⁻¹).
- x_i: Mass fraction of component i (unitless).
- $C_{p,i}$: Specific heat capacity of component i (J·kg⁻¹·K⁻¹).

- N: Total number of components.

Equation 46: Pressure difference for pump.

$$\Delta P = P_{out} - P_{in}$$

Where:

- ΔP: Pressure difference (Pa).
- P_{out}: Outlet pressure (Pa).
- P_{in}: Inlet pressure (Pa).

Equation 47: Head developed for pump.

$$H = \frac{\Delta P}{\rho_{mixture} \cdot g}$$

Where:

- H: Head developed (m).
- ΔP: Pressure difference (Pa).
- $\rho_{mixture}$: Mixture density ($kg \cdot m^{-3}$).
- g: Gravitational acceleration ($9.81\ m \cdot s^{-2}$).

Equation 48: Isentropic power for pump.

$$W_{isentropic} = \frac{\dot{m}_{total} \cdot \Delta P}{\rho_{mixture}}$$

Where:

- $W_{isentropic}$: Isentropic power ($J \cdot s^{-1}$).
- $\dot{m}_{total}$: Total mass flow rate ($kg \cdot s^{-1}$).
- ΔP: Pressure difference (Pa).
- $\rho_{mixture}$: Mixture density ($kg \cdot m^{-3}$).

Equation 49: Actual power (electricity required) for pump.

$$W_{actual} = \frac{W_{isentropic}}{\eta}$$

Where:

- W_{actual}: Actual power (electricity required) ($J \cdot s^{-1}$).
- $W_{isentropic}$: Isentropic power ($J \cdot s^{-1}$).
- η: Pump efficiency (unitless).

Equation 50: Temperature rise for pump.

$$\Delta T = \frac{W_{actual}}{\dot{m}_{total} \cdot C_{p,mixture}}$$

Where:

- ΔT: Temperature rise (K).
- W_{actual}: Actual power (electricity required) (J·s⁻¹).
- $\dot{m}_{total}$: Total mass flow rate (kg·s⁻¹).
- $C_{p,mixture}$: Specific heat capacity of mixture (J·kg⁻¹·K⁻¹).

Equation 51: Outlet temperature for pump.

$$T_{out} = T_{in} + \Delta T$$

Where:

- T_{out}: Outlet temperature (K).
- T_{in}: Inlet temperature (K).
- ΔT: Temperature rise (K).

Heat Exchanger

Equation 52: Heat duty for heater/cooler.

$$Q = \dot{m}_{total} \cdot (h_{out} - h_{in})$$

Where:

- Q: Heat duty (J·s⁻¹).
- $\dot{m}_{total}$: Total mass flow rate (kg·s⁻¹).
- h_{in}: Inlet specific enthalpy(J·kg⁻¹).
- h_{out}: Outlet Specific enthalpy(J·kg⁻¹).

Combustion

Equation 53: Specific calorific value of a single component for combustion.

$$CV = \frac{|\Delta H_c|}{MW}$$

Where:

- CV: Specific calorific value (J·kg⁻¹).
- ΔH_c: Enthalpy of combustion (J·mol⁻¹).
- MW: Molecular weight (kg·mol⁻¹).

Equation 54: Mixture calorific value for combustion.

$$CV_{mixture} = \sum_{i=1}^{N} x_i \cdot CV_i$$

Where:

- $CV_{mixture}$: Mixture specific calorific value (J·kg⁻¹).
- x_i: Mass fraction of component i (unitless).
- CV_i: Specific calorific value of component i (J·kg⁻¹).

Equation 55: Amount of mixture ($kg \cdot h^{-1}$) to combust and generate Q ($J \cdot s^{-1}$) of energy for the block.

$$\dot{m}_{total} = \frac{Q}{CV_{mixture} \cdot \eta}$$

Where:

- $\dot{m}_{total}$: Total mass flow rate ($kg \cdot s^{-1}$).
- Q: Energy demand of the block of interest ($J \cdot s^{-1}$).
- $CV_{mixture}$: Mixture specific calorific value ($J \cdot kg^{-1}$).
- η: Efficiency of block i (unitless).

Equation 56: Mixture specific heat capacity for combustion products.

$$C_{p,mixture} = \sum_{i=1}^{N} x_i \cdot C_{p,i}$$

Where:

- $C_{p,mixture}$: Specific heat capacity of mixture ($J \cdot kg^{-1} \cdot K^{-1}$).
- x_i: Mass fraction of component i (unitless).
- $C_{p,i}$: Specific heat capacity of component i ($J \cdot kg^{-1} \cdot K^{-1}$).
- N: Total number of components.

Equation 57: Heat released during combustion.

$$\dot{Q}_{released} = \dot{m}_{fuel} \cdot CV_{mixture}$$

Where:

- $\dot{Q}_{released}$: Heat released during combustion ($J \cdot s^{-1}$).
- $\dot{m}_{fuel}$: Mass fraction of fuel ($kg \cdot s^{-1}$).
- $CV_{mixture}$: Mixture specific calorific value ($J \cdot kg^{-1}$).

Equation 58: Iterative calculation for outlet temperature (T_{out}) using an initial guess (numerical solution).

$$\dot{Q}_{released} - \dot{m}_{total} \cdot C_{p,mixture} \cdot (T_{out} - T_{in}) = 0$$

Where:

- $Q_{released}$: Heat released during combustion (J).
- $\dot{m}_{total} = \dot{m}_{fuel} + \dot{m}_{air}$: Total mass flow rate ($kg \cdot h^{-1}$).
- T_{out}: Outlet temperature (K).
- T_{in}: Inlet temperature (K).

CO_2 emissions

Equation 59: Total carbon fraction (TCF).

$$\mathrm{TCF} = \sum_{i=1}^{N} x_i \cdot C_i$$

Where:

- TCF: Total carbon fraction (unitless).
- x_i: Mass fraction of component i (unitless).
- C_i: Carbon fraction of component i (unitless).

Equation 60: CO_2 emissions per kg of fuel.

$$CO_2 \text{ emissions } \left(\frac{\text{kg } CO_2}{\text{kg fuel}}\right) = \mathrm{TCF} \cdot \frac{M_{CO_2}}{M_C}$$

Where:

- CO_2 emissions: kg CO_2 / kg fuel.
- TCF: Total carbon fraction (unitless).
- M_{CO_2}: Molecular weight of carbon dioxide (kg·mol^{-1}).
- M_C: Molecular weight of carbon (kg·mol^{-1}).

Distillation

Modeling

Due to the complexity of modeling distillation columns, the equations are presented in a separate section, along with graphical representations to facilitate a clear and detailed understanding of the adopted mathematical approach.[4] SI units are used in all mass and energy balances.

Equation 61: Mass balance in stage j.

$$F_j + V_{j+1} + L_{j-1} - V_j - W_j - L_j - U_j = 0$$

Where:

- F_j: Flow rate of the feed stream to stage j.
- L_j: Liquid flow rate outputting stage j and inputting stage $j + 1$.
- V_j: Vapor flow rate outputting stage j and inputting stage $j - 1$.
- U_j: Liquid side flow rate outputting state j.
- W_j: Vapor side flow rate outputting stage j.

Figure S1 shows a schematic representation of a distillation column. The side streams U and W are used to represent the output streams like distillate and bottoms (i.e., D and B). These streams can occur as liquid (L_B and L_D) or vapor (V_B and V_D), in which $D = L_D + V_D$ and $B = L_B + V_B$. The column consists of n stages, with the stage numbering starting on condenser ($j = 1$) and continuing until reboiler is reached ($j = n$).

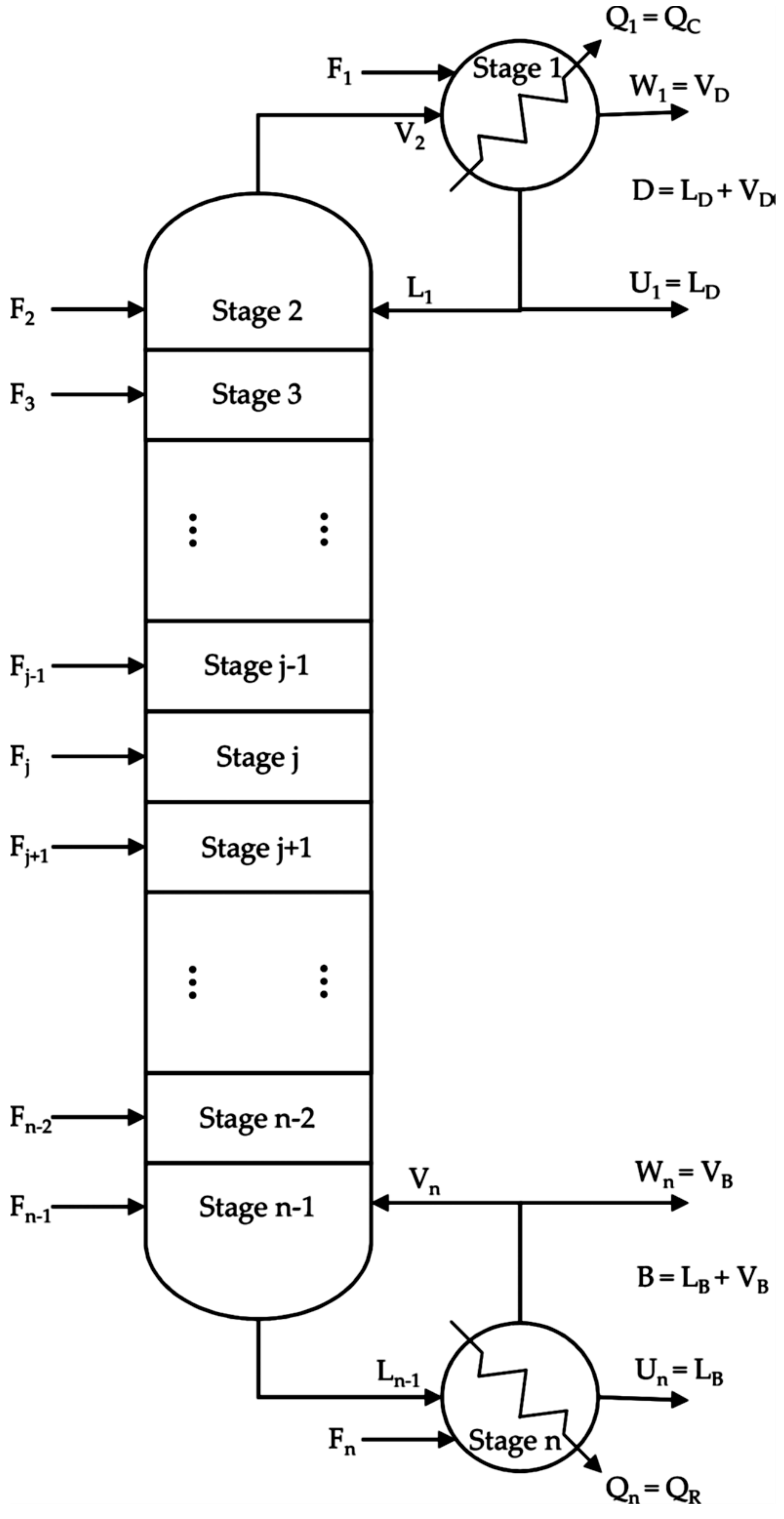

Figure S1: Schematic representation of a distillation column. Reproduced from[4] under CC BY 3.0.

Figure S2 shows a schematic representation of the input and output streams in all stages.

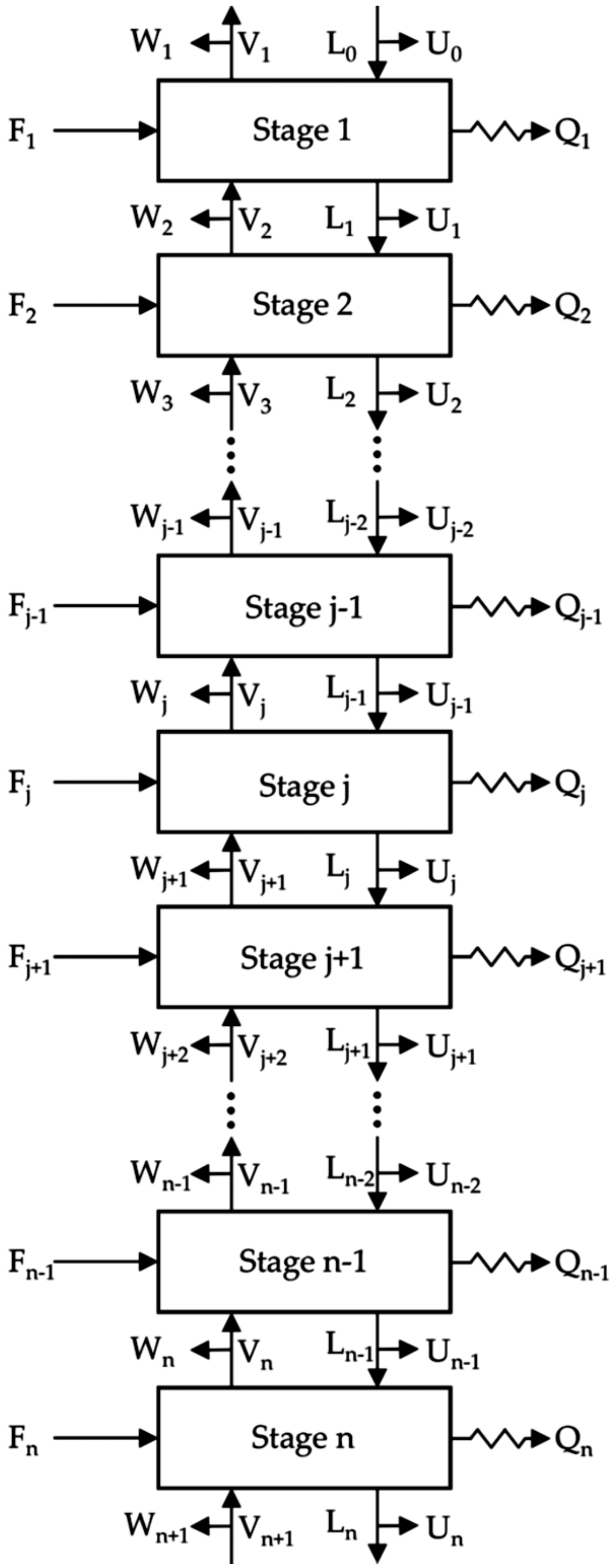

Figure S2: Schematic representation of input and output streams in all stages of a distillation column. Reproduced from[4] under CC BY 3.0.

Equation 62: Global mass balance.

$$\sum_{j=1}^{n} F_j - \sum_{j=1}^{n} (W_j + U_j) = 0$$

Where:

- F_j: Flow rate of the feed stream to stage j.
- U_j: Liquid side flow rate outputting stage j.
- W_j: Vapor side flow rate outputting stage j.
- n: Total number of stages.

Equation 63: Global mass balance (side streams in condenser and reboiler).

$$\sum_{j=1}^{n} F_j - B - D - \sum_{j=2}^{n-1} (W_j + U_j) = 0$$

Where:

- F_j: Flow rate of the feed stream to stage j.
- B: Bottoms stream.
- D: Distillate stream.
- U_j: Liquid side flow rate outputting stage j.
- W_j: Vapor side flow rate outputting stage j.
- n: Total number of stages.

Equation 64: Mass balance of component i in stage j.

$$z_{i,j} \cdot F_j + y_{i,j+1} \cdot V_{j+1} + x_{i,j+1} \cdot L_{j-1} - y_{i,j} \cdot (V_j + W_j) - x_{i,j} \cdot (L_j - U_j) = 0$$

Where:

- F_j: Flow rate of the feed stream to stage j.
- L_j: Liquid flow rate outputting stage j and inputting stage $j + 1$.
- V_j: Vapor flow rate outputting stage j and inputting stage $j - 1$.
- U_j: Liquid side flow rate outputting stage j.
- W_j: Vapor side flow rate outputting stage j.
- $x_{i,j}$: Fraction of component i in the liquid phase of stage j.
- $y_{i,j}$: Fraction of component i in the vapor phase of stage j.
- $z_{i,j}$: Fraction of component i in the feed stream of stage j.

Equation 65: Mass balance of component i in stage j (side streams in condenser and reboiler).

$$\sum_{j=1}^{n} z_{i,j} \cdot F_j - x_{i,n} \cdot L_B - y_{i,n} \cdot V_B - x_{i,1} \cdot L_D + y_{i,1} \cdot V_D - \sum_{j=2}^{n-1} (x_{i,j} \cdot W_j + x_{i,j} \cdot U_j) = 0$$

- n: Total number of stages.

Other notations like Equation 64. The subscripts B and D denote the bottoms and distillate streams, respectively.

Equation 66: Energy balance in stage j.

$$F_j \cdot H_j^F + V_{j+1} \cdot H_{j+1}^V + L_{j-1} \cdot H_{j-1}^L - (V_j - W_j) \cdot H_j^V - (L_j - U_j) \cdot H_j^L - Q_j = 0$$

Where:

- F_j: Flow rate of the feed stream to stage j.
- L_j: Liquid flow rate outputting stage j and inputting stage $j+1$.
- V_j: Vapor flow rate outputting stage j and inputting stage $j-1$.
- U_j: Liquid side flow rate outputting stage j.
- W_j: Vapor side flow rate outputting stage j.
- H_j^F: Enthalpy of the feed stream to stage j.
- H_j^V: Enthalpy of the vapor stream to stage j.
- H_j^L: Enthalpy of the liquid stream to stage j.

Equation 67: Global energy balance.

$$\sum_{j=1}^{n} F_j \cdot H_j^F - \sum_{j=1}^{n} \left(W_j \cdot H_j^V + U_j \cdot H_j^L + Q_j\right) = 0$$

Equation 68: Global energy balance (expanded form).

$$\sum_{j=1}^{n} F_j \cdot H_j^F - L_B \cdot H_n^L - V_B \cdot H_n^V - L_D \cdot H_1^L - V_D \cdot H_1^V - Q_C - Q_R$$
$$- \sum_{j=2}^{n-1} \left(W_j \cdot H_j^V + U_j \cdot H_j^L + Q_j\right) = 0$$

Where:

- F_j: Flow rate of the feed stream to stage j.
- L_j: Liquid flow rate outputting stage j and inputting stage $j+1$.
- V_j: Vapor flow rate outputting stage j and inputting stage $j-1$.
- U_j: Liquid side flow rate outputting stage j.
- W_j: Vapor side flow rate outputting stage j.
- H_j^F: Enthalpy of the feed stream to stage j.
- H_j^V: Enthalpy of the vapor stream to stage j.
- H_j^L: Enthalpy of the liquid stream to stage j.
- Q_C: Heat duty of condenser.
- Q_R: Heat duty of reboiler.
- n: Total number of stages.

The subscripts B and D denote the bottoms and distillate streams, respectively.

Equation 69: Reflux ratio.

$$r_D = \frac{L_1}{D}$$

Where:

- r_D: Reflux ratio.
- L_1: Liquid flow rate at stage 1.
- D: Distillate stream.

Equation 70: Reboiler ratio.

$$r_B = \frac{V_n}{B}$$

- r_D: Reboil ratio.
- V_n: Vapor flow rate at stage n.
- B: Bottoms stream.

Equation 71: Ratio between the side stream and the stream outputting a stage and inputting the near stages.

$$d_j = \frac{R_j}{L_j + V_j} = \frac{U_j + W_j}{L_j + V_j} = \frac{\omega_j \cdot R_j + (1 - \omega_j) \cdot R_j}{L_j + V_j}$$

Where:

- d_j: Ratio between the side stream and the stream outputting a stage and inputting the near stages.
- L_j: Liquid flow rate outputting stage j and inputting stage $j + 1$.
- V_j: Vapor flow rate outputting stage j and inputting stage $j - 1$.
- $U_j = \omega_j \cdot R_j$: Liquid side flow rate outputting stage j.
- $W_j = (1 - \omega_j) \cdot R_j$: Vapor side flow rate outputting stage j.
- R_j: Sum of liquid side stream U_j and vapor stream W_j.
- ω_j: Side stream.

When considering side streams, there are four possibilities: no side stream, only a liquid side stream, only a vapor side stream, or both liquid and vapor side streams. Further details are provided in Table S2 and Figure S3.

Table S2: Four types of side streams.[4]

Stage Type	No Side Stream	Only Liquid Side Stream	Only Vapor Side Stream	Vapor and Liquid Side Streams
Generic (stage j)	$d_j = 0$	$d_j \neq 0$	$d_j \neq 0$	$d_j \neq 0$
	$W_j = 0$	$\omega_j = 0$	$\omega_j = 1$	$0 < \omega_j < 1$
	$U_j = 0$	$R_j = d_j \cdot (L_j + V_j)$	$R_j = d_j \cdot (L_j + V_j)$	$R_j = d_j \cdot (L_j + V_j)$
		$W_j = 0$	$W_j = R_j$	$W_j = \omega_j \cdot R_j$

		$U_j = R_j$	$U_j = 0$	$U_j = (1 - \omega_j) \cdot R_j$
Condenser (j = 1)	Total reflux	Total condenser	Partial condenser	Partial condenser
	$d_1 = 0$	$d_1 = 1/r_D$	$d_1 = 1/r_D$	$d_1 = 1/r_D$
	$W_1 = V_D = 0$	$\omega_1 = \omega_D = 0$	$\omega_1 = \omega_D = 1$	$0 < \omega_1 = \omega_D < 1$
	$U_1 = L_D = 0$	$R_1 = d_1 \cdot L_1 = D$	$R_1 = d_1 \cdot L_1 = D$	$R_1 = d_1 \cdot L_1 = D$
		$W_1 = V_D = 0$	$W_1 = V_D = R_1$	$W_1 = V_D = \omega_D \cdot R_1$
		$U_1 = L_D = R_1$	$U_1 = L_D = 0$	$U_1 = L_D = (1 - \omega_D) \cdot R_1$
Reboiler (j = n)	Total reflux	Partial reboiler	Total reboiler	Partial reboiler
	$d_n = 0$	$d_n = 1/r_B$	$d_n = 1/r_B$	$d_n = 1/r_B$
	$W_n = V_B = 0$	$\omega_n = \omega_B = 0$	$\omega_n = \omega_B = 1$	$0 < \omega_n = \omega_B < 1$
	$U_n = L_B = 0$	$R_n = d_n \cdot V_n = B$	$R_n = d_n \cdot V_n = B$	$R_n = d_n \cdot V_n = B$
		$W_n = V_B = 0$	$W_n = V_B = R_n$	$W_n = V_B = \omega_B \cdot R_n$
		$U_n = L_B = R_n$	$U_n = L_B = 0$	$U_n = L_B = (1 - \omega_B) \cdot R_n$

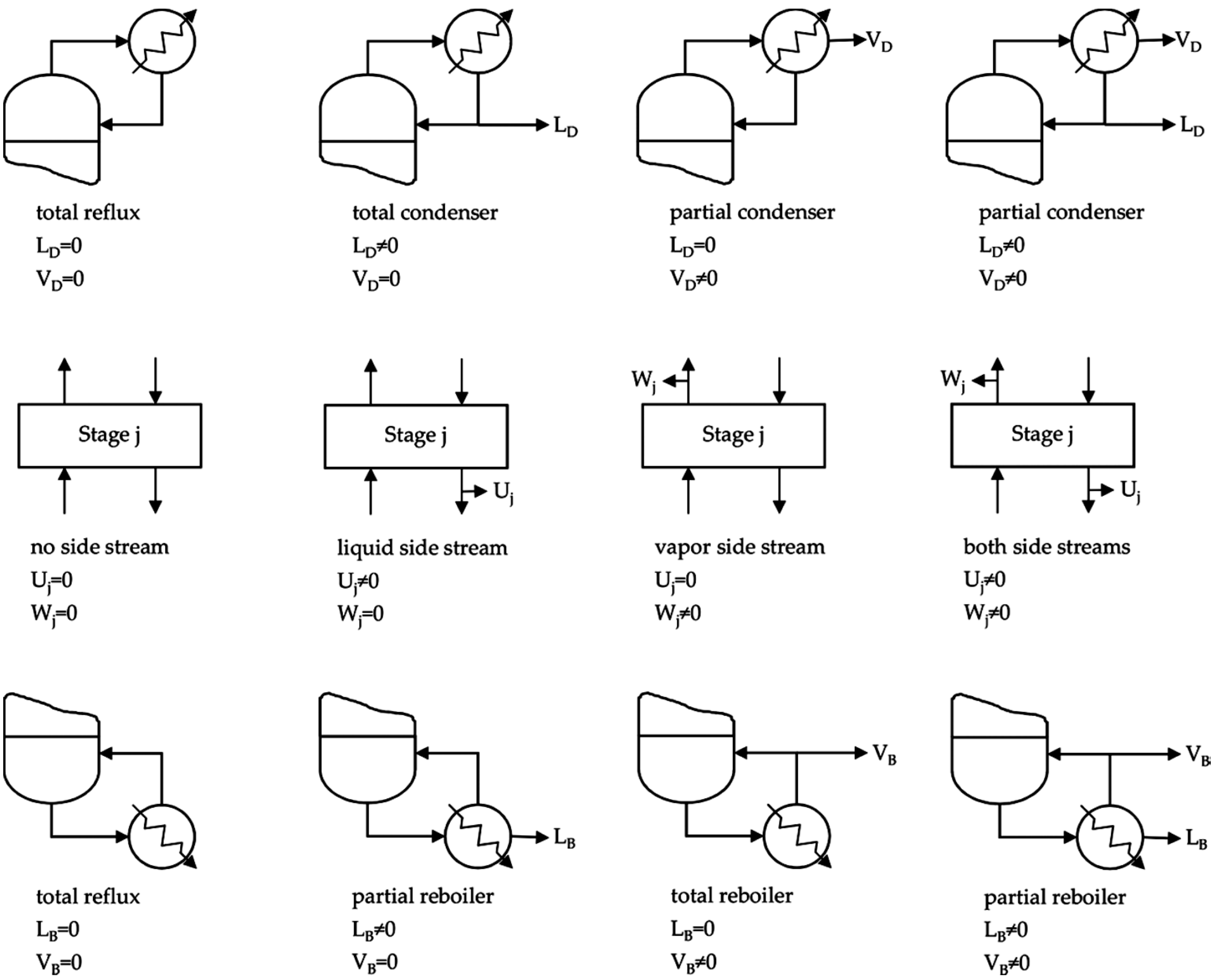

Figure S3: Four types of side streams. Reproduced from[4] under CC BY 3.0.

Thermodynamics

Thermodynamics is essential for modeling phase equilibrium.

Equation 72: Fugacity relation.

$$f_i^L = f_i^V$$

Where:

- f_i^L: Fugacity of component i in liquid phase.
- f_i^V: Fugacity of component i in vapor phase.

Equation 73: Fraction of component i in each phase.

$$y_i = K_i \cdot x_i$$

Where:

- y_i: The fraction of component i in the vapor phase.
- K_i: Kappa of component i, which depends on the chosen vapor liquid equilibrium formulation.
- x_i: Fraction of component i in the liquid phase.

Equation 74: Phi/phi formulation for Kappa.

$$K_i = \frac{\hat{\varphi}_i^L}{\hat{\varphi}_i^V}$$

Where:

- K_i: Kappa of component i.
- $\hat{\varphi}_i^L$: Fugacity coefficient of component i in liquid phase.
- $\hat{\varphi}_i^V$: Fugacity coefficient of component i in vapor phase.

Equation 75: Gamma/phi formulation for Kappa.

$$K_i = \frac{\gamma_i \cdot \varphi_i^{sat} \cdot P_i^{sat}}{\hat{\varphi}_i^V} \exp\left(\int_{P_i^{sat}}^{P} \frac{V_i^L}{R \cdot T} dP \right)$$

Where:

- K_i: Kappa of component i.
- γ_i: Activity coefficient of component i in liquid phase.
- φ_i^{sat}: Fugacity coefficient of pure component i in saturation state.
- P_i^{sat}: Saturated vapor pressure of component i.
- $\hat{\varphi}_i^V$: Fugacity coefficient of component i in vapor phase.
- V_i^L: Volume of component i in liquid phase.
- R: Ideal gas constant.
- T: Temperature.

Equation 76: Liquid enthalpy.

$$H^L = \sum_{i=1}^{m} x_i \cdot H_i^L + H^E$$

Where:

- H^L: Liquid enthalpy.
- x_i: Fraction of component i in the liquid phase.
- H_i^L: Fugacity coefficient of component i in liquid phase.
- H^E: Excess enthalpy calculated by a model that represents the excess Gibbs free energy.
- m: Total number of components.

Equation 77: Vapor enthalpy.

$$H^V = \sum_{i=1}^{m} y_i \cdot H_i^V + H^R$$

- H^V: Vapor enthalpy.
- y_i: Fraction of component i in the vapor phase.
- H_i^v: Fugacity coefficient of component i in vapor phase.
- H^R: Residual enthalpy calculated using equation of state.
- m: Total number of components.

To calculate the enthalpy of a pure component i, a reference state must be specified. Since no reaction occurs in the column, it is possible to set the enthalpies of all components to zero at a reference state under the same temperature, pressure, and phase conditions. However, to make the model more general and applicable to reactive systems, an elemental reference state is chosen. This reference state, defined at 298 K and 1 atm, assigns a standard enthalpy of formation (ΔH_f) of zero, by convention, to elements in their most stable standard state. The enthalpy of a component is calculated relative to this elemental reference state, as shown in Figure S4.

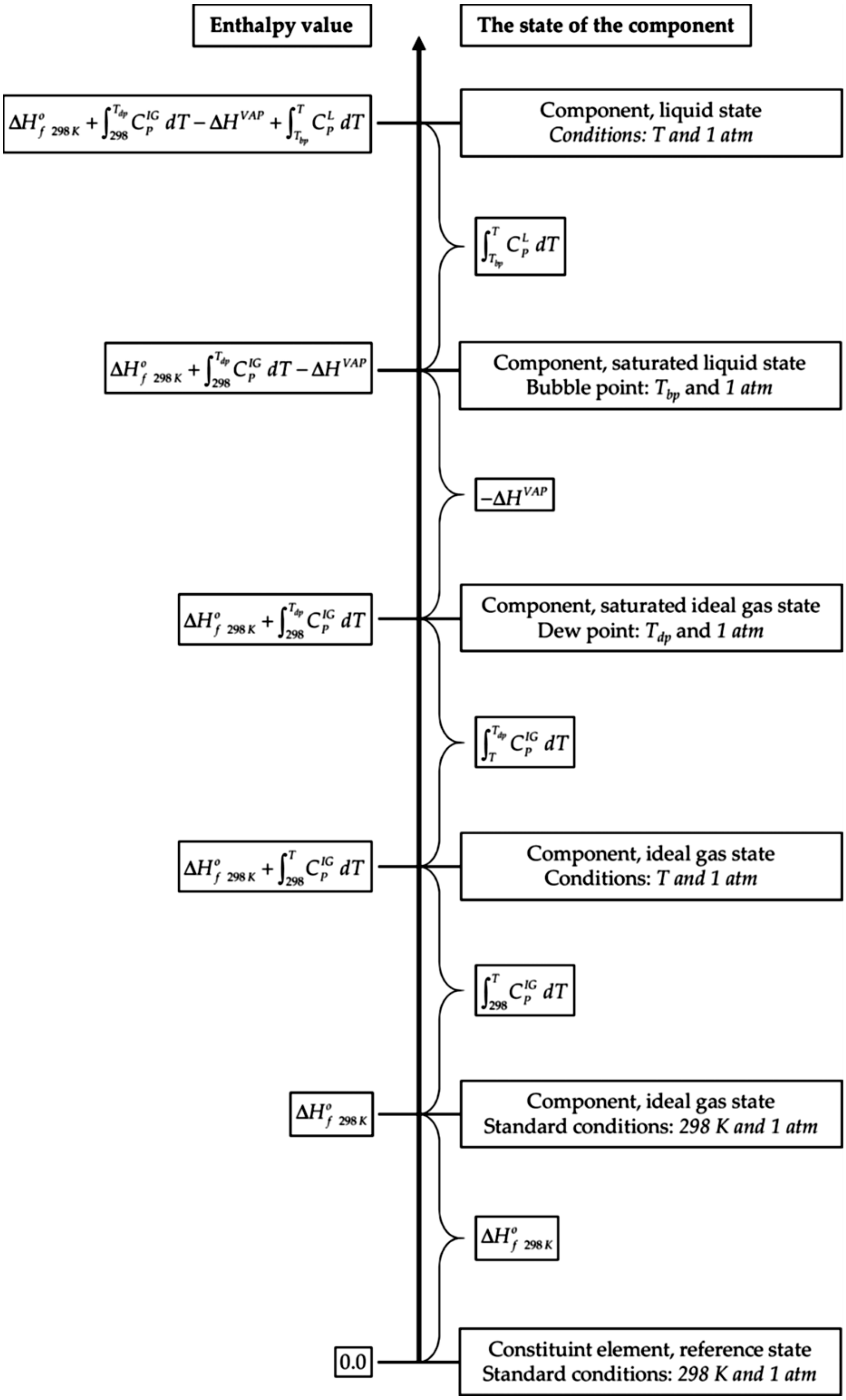

Figure S4: Calculation of the enthalpy of a component based on the elemental reference state. Reproduced from[4] under CC BY 3.0.

In this calculation, C_p represents the heat capacity at constant pressure for liquid (L) and ideal gas (IG), T_{bp} is the bubble point temperature, T_{dp} is the dew point temperature, and ΔH^{VAP} is the enthalpy of vaporisation.

Methodology

This report adopts the methodology presented by Steffen et al.[4] for modeling steady-state multicomponent distillation columns. This approach emphasizes the use of simplified models to generate accurate initial estimates for variables such as stage temperatures and flow rates, utilizing saturation temperature and non-heat effects models. Good initial estimates are crucial in solving the highly nonlinear equations that govern distillation processes, as they significantly enhance the convergence and stability of numerical methods like Newton-Raphson. By simplifying the tearing equations, this methodology ensures computational efficiency while maintaining accuracy, making it suitable for complex distillation systems.

Equation 78: Average temperature pondered on component fractions in feed streams (linear profile).

$$T_{ave} = \frac{\sum_{i=1}^{m}\left(T_i^{SAT} \cdot \sum_{j=1}^{n} z_{i,j} \cdot F_j\right)}{\sum_{j=1}^{n} F_j}$$

Where:

- T_{ave}: Average temperature pondered on component fractions in feed streams.
- T_i^{SAT}: Saturation temperature of pure components at column operation pressure.
- $z_{i,j}$: Fraction of component i in the feed stream of stage j.
- F_j: Flow rate of the feed stream to stage j.
- m: Total number of components.
- n: Total number of stages.

Equation 79: Minimal temperature where ADD is the average absolute deviation.

$$T_{min} = T_{ave} - ADD = T_{ave} - \frac{\sum_{i=1}^{m}\left(\left|T_i^{SAT} - T_{ave}\right| \sum_{j=1}^{n} z_{i,j} \cdot F_j\right)}{\sum_{j=1}^{n} F_j}$$

Notations like Equation 78.

Equation 80: Initial estimates of stage temperatures.

$$T_j^{(0)} = T_{min} + \frac{2 \cdot (j-1)}{n} \cdot (T_{ave} - T_{min})$$

Notations like Equation 78.

In the non-heat effects model, it is assumed that the vaporisation enthalpy remains constant. This ensures that changes in the liquid and vapor flow rates within each

section of the column are solely influenced by the feed quality, feed flow rate, and side flow rates, as illustrated in Figure S5.

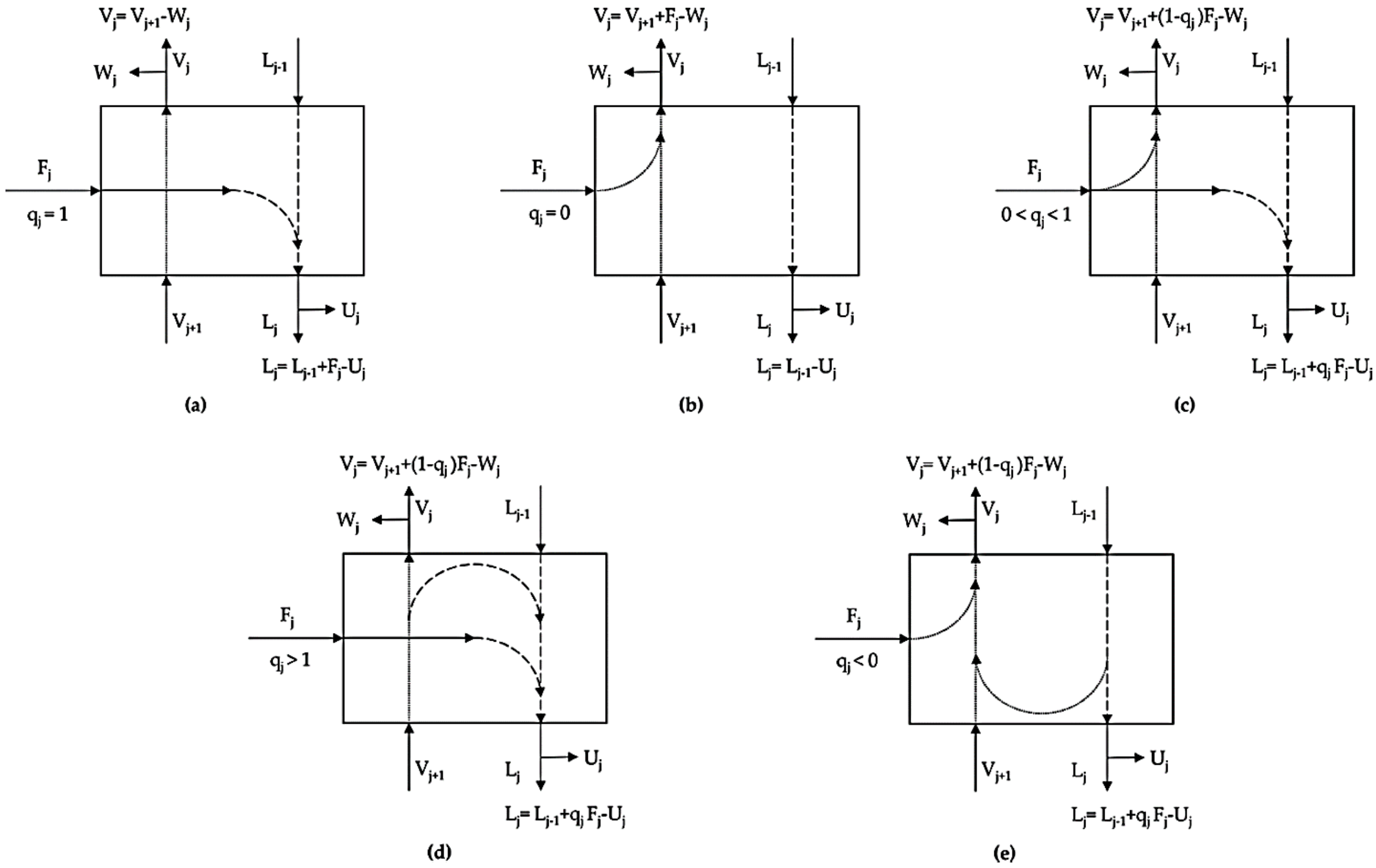

Figure S5: Non-heat effect behavior in a stage for: (a) saturated liquid; (b) saturated vapor; (c) saturated liquid and vapor; (d) subcooled liquid and (e) superheated vapor. Reproduced from[4] under CC BY 3.0.

The feed quality is defined as:

Equation 81: Saturated liquid.

$$q = 1$$

Where:

- q: Feed quality of the stream entering the distillation column.

Equation 82: Saturated vapor.

$$q = 0$$

Notation like Equation 81.

Equation 83: Saturated liquid and vapor, q is equal to liquid fraction.

$$0 < q < 1$$

Notation like Equation 81.

Equation 84: Subcooled liquid, $q > 1$.

$$q = \frac{\left(\Delta H^{VAP} + \int_{T_{bp}}^{T} C_p^L dT\right)}{\Delta H^{VAP}}$$

Where:

- q: Feed quality of the stream entering the distillation column.
- ΔH^{VAP}: Enthalpy of vaporisation.
- T: Feed temperature.
- T_{bp}: Bubble point temperature.
- C_p^L: Liquid heat capacity at constant pressure.

Equation 85: Superheated vapor, $q < 0$.

$$q = \frac{-\left(\int_{T_{dp}}^{T} C_p^V dT\right)}{\Delta H^{VAP}}$$

Where:

- q: Feed quality of the stream entering the distillation column.
- ΔH^{VAP}: Enthalpy of vaporisation.
- T: Feed temperature.
- T_{bp}: Bubble point temperature.
- C_p^V: Vapor heat capacity at constant pressure.

Equation 86: Total side stream flow rate leaving stage j.

$$R_j = \frac{\frac{d_j}{d_j + 1}\left(F_j + V_{j+1} + L_{j-1}\right)}{\sum_{j=1}^{n} \frac{d_j}{d_j + 1}\left(F_j + V_{j+1} + L_{j-1}\right)} \cdot \sum_{j=1}^{n} F_j$$

Where:

- R_j: Total side stream flow rate at stage j.
- d_j: Ratio between the side stream and the total outgoing stream from stage j.
- V_{j+1}: Vapor flow rate entering stage j.
- L_{j-1}: Liquid flow rate entering stage j.
- n: Total number of stages in the column.

Equation 87: Initial estimates for side flow rates.

$$R_j^{(0)} = \frac{d_j}{\sum_{j=1}^{n} d_j} \cdot \sum_{j=1}^{n} F_j$$

Notation like Equation 86.

If side streams are present only in the reboiler and condenser, or if the side streams in the intermediate stages are already known, the non-heat effects model can be used to directly generate initial estimates for the bottoms and distillate flow rates.

Equation 88: Initial estimates for the bottoms flow rates.

$$B^{(0)} = \frac{r_D \cdot F_1 + (r_D + 1) \cdot F_n + \sum_{j=2}^{n-1} \left[\left(q_j + r_D \right) \cdot F_j - (r_D + 1) \cdot U_j - W_j \right]}{r_D + r_B + 1}$$

Where:

- $B^{(0)}$: Initial estimate of the bottoms flow rate.
- r_D: Reflux ratio.
- F_j: Feed flow rate to stage j.
- U_j: Liquid side stream flow rate leaving stage j.
- W_j: Vapor side stream flow rate leaving stage j.
- r_B: Reboil ratio.
- q_j: Feed quality at stage j (fraction of feed in the liquid phase).

Equation 89: Initial estimates for the distillate flow rates.

$$D^{(0)} = \frac{(r_B + 1) \cdot F_1 + r_D \cdot F_n + \sum_{j=2}^{n-1} \left[\left(r_B + 1 - q_j \right) \cdot F_j + (r_D + 1) \cdot U_j + W_j \right]}{r_D + r_B + 1}$$

Where:

- $D^{(0)}$: Initial estimate of the distillate flow rate.
- r_D: Reflux ratio.
- F_j: Feed flow rate to stage j.
- U_j: Liquid side stream flow rate leaving stage j.
- W_j: Vapor side stream flow rate leaving stage j.
- r_B: Reboil ratio.
- q_j: Feed quality at stage j (fraction of feed in the liquid phase).

Equation 90: Initial estimate of the vapor flow rate leaving the reboiler.

$$V_n^{(0)} = r_B \cdot B^{(0)}$$

Where:

- $V_n^{(0)}$: Initial estimate of the vapor flow rate leaving the reboiler (stage n).
- r_B: Reboil ratio.
- $B^{(0)}$: Initial estimate of the bottoms flow rate.

Equation 91: Initial estimates for the distillate flow rates.

$$L_1^{(0)} = r_D \cdot D^{(0)}$$

Where:

- $L_1^{(0)}$: Initial estimate of the liquid flow rate leaving the condenser (stage 1).
- r_D: Reflux ratio.
- $D^{(0)}$: Initial estimate of the distillate flow rate.

Equation 92: Initial estimate of the vapor flow rate at stage j.

$$V_j^{(0)} = \frac{(1 - q_j) \cdot F_j + V_{j+1}^{(0)}}{d_j + 1}$$

Where:

- $V_j^{(0)}$: Initial estimate of the vapor flow rate at stage j.
- q_j: Feed quality at stage j (fraction of feed in the liquid phase).
- F_j: Feed flow rate to stage j.
- $V_{j+1}^{(0)}$: Initial estimate of the vapor flow rate entering stage j from stage $j + 1$.
- d_j: Ratio of the side stream flow to the output streams from stage j.

Equation 93: Initial estimate of the liquid flow rate at stage j.

$$L_j^{(0)} = \frac{F_j + V_{j+1}^{(0)} + L_{j-1}^{(0)}}{d_j + 1} - V_j^{(0)}$$

Where:

- $L_j^{(0)}$: Initial estimate of the liquid flow rate at stage j.
- F_j: Feed flow rate to stage j.
- $V_{j+1}^{(0)}$: Initial estimate of the vapor flow rate entering stage j from stage $j + 1$.
- $L_{j-1}^{(0)}$: Initial estimate of the liquid flow rate entering stage j from stage $j - 1$.
- $V_j^{(0)}$: Initial estimate of the vapor flow rate at stage j.
- d_j: Ratio of the side stream flow to the output streams from stage j.

Algorithm

Solving algebraic equations requires the number of unknowns to equal the number of equations. Table S3 shows this balance of equations and unknowns.

Table S3: Balance of equations and unknowns.[4]

**Unknown Type**	**Number of Unknowns**	**Stage**	**Equation(s) Used**
Temperatures	n	1 to n	The restriction that the sum of vapor mole fraction is equal to unity, for stages 1 to n.
Vapor flow rates	$n - 1$	2 to n	Energy balance on stages to $n - 1$ and mass balance on stage n.
Liquid flow rates	$n - 1$	1 to $n - 1$	Mass balance for stage 1 to $n - 1$.
Slide flow rates (liquid and vapor)	$2 \cdot n$	1 to n	Ratio between side steams and streams outputting stages for n stages. And vapor fraction of side stream for n stages.
Liquid mole fractions	$n \cdot m$	1 to n	Mass balance by component in n stages for m components.

Vapor mole fractions	$n \cdot m$	1 to n	Iso-fugacity relation for m components in n stages.
Heat transfer	2	1 and n	Energy balance in 1 stages and n.
Total	$(2 \cdot m + 5) \cdot n$	n	$(2 \cdot m + 5) \cdot n$

The sequence of calculations of the adopted algorithm is presented in Figure S6.

Equation 94: Mole fraction of component i in the vapor phase at stage j.

$$y_{i,j} = K_{i,j} \cdot x_{i,j}$$

Where:

- $y_{i,j}$: Mole fraction of component i in the vapor phase at stage j.
- $x_{i,j}$: Mole fraction of component i in the liquid phase at stage j.
- $K_{i,j}$: Equilibrium ratio (distribution coefficient) of component i at stage j.

Equation 95: Liquid/vapor coefficients relationship.

$$l_j \cdot x_{i,j-1} + m_j \cdot x_{i,j} + u_j \cdot x_{i,j} = b_j$$

Where:

- l_j: Coefficient related to the liquid flow rate from the previous stage.
- $x_{i,j}$: Mole fraction of component i in the liquid phase at stage j.
- m_j: Coefficient accounting for the sum of liquid and vapor side stream effects.
- u_j: Coefficient related to the vapor flow rate from the next stage.
- b_j: Right-hand side term representing the contribution of the feed mole fractions.

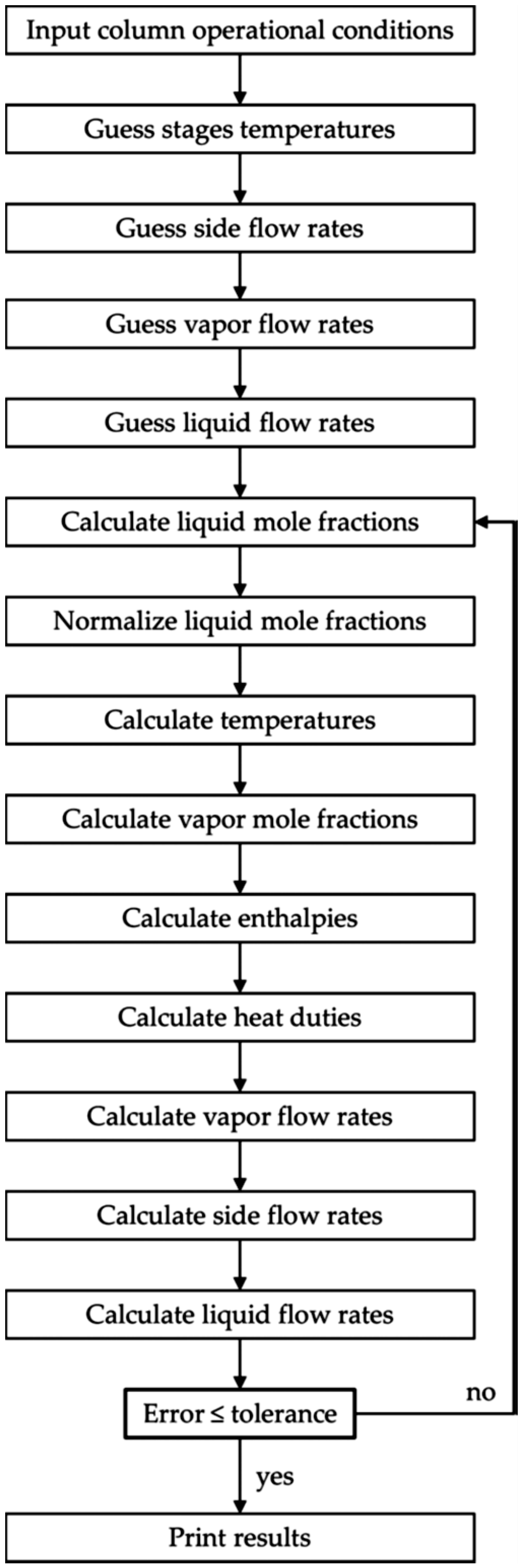

Figure S6: Algorithm for the simulation of steady-state distillation columns. Reproduced from[4] under CC BY 3.0.

Equation 96: Liquid diagonal coefficient in the tridiagonal system.

$$l_j = L_{j-1}$$

Where:

- l_j: Lower diagonal coefficient in the tridiagonal system.

- L_{j-1}: Liquid flow rate entering stage j from stage $j-1$.

Equation 97: Coefficient in the tridiagonal linear system of equations used to solve for component mole fractions in each stage.

$$\alpha_j = -\left[\left(L_j + U_j\right) + \left(V_j + W_j\right)\right] \cdot K_{i,j}$$

Where:

- α_j: Coefficient in the tridiagonal linear system of equations used to solve component mole fractions in each stage.
- L_j: Liquid flow rate leaving stage j.
- U_j: Liquid side stream flow rate from stage j.
- V_j: Vapor flow rate leaving stage j.
- W_j: Vapor side stream flow rate from stage j.
- $K_{i,j}$: Equilibrium ratio (distribution coefficient) of component i at stage j.

Equation 98: Upper diagonal coefficient in the tridiagonal matrix.

$$u_j = V_{j+1} \cdot K_{i,j+1}$$

Where:

- u_j: Upper diagonal coefficient in the tridiagonal matrix.
- V_{j+1}: Vapor flow rate entering stage j from stage $j+1$.
- $K_{i,j+1}$: Equilibrium ratio of component i at stage $j+1$.

Equation 99: Right-hand side term in the tridiagonal system.

$$b_j = -F_j \cdot z_{i,j}$$

Where:

- b_j: Right-hand side term in the tridiagonal system.
- F_j: Feed flow rate to stage j.
- $z_{i,j}$: Mole fraction of component i in the feed stream to stage j.

Equation 100: Triagonal linear system that solves for the mole fractions of component i in the liquid phase across all stages in the column. It is solved iteratively to determine the distribution of components in the distillation process.

$$\begin{bmatrix} \alpha_1 & u_1 & 0 & 0 & 0 \\ l_2 & \alpha_2 & u_2 & 0 & 0 \\ 0 & \ddots & \ddots & \ddots & 0 \\ 0 & 0 & l_{n-1} & \alpha_{n-1} & u_{n-1} \\ 0 & 0 & 0 & l_n & \alpha_n \end{bmatrix} \cdot \begin{bmatrix} x_{i,1} \\ x_{i,2} \\ \vdots \\ x_{i,n-1} \\ x_{i,n} \end{bmatrix} = \begin{bmatrix} b_1 \\ b_2 \\ \vdots \\ b_{n-1} \\ b_n \end{bmatrix}$$

Equation 101: Normalised mole fraction of component i in the liquid phase at stage j.

$$x_{i,j} = -F_j \cdot z_{i,j}$$

Where:

- $x_{i,j}$: Normalised mole fraction of component i in the liquid phase at stage j.
- m: Total number of components.

Equation 102: Function that ensures the sum of vapor-phase mole fractions equals unity.

$$F(T_j) = 1 - \sum_{i=1}^{m} K_{i,j} \cdot x_{i,j} = 0$$

Where:

- $F(T_j)$: Function that ensures the sum of vapor-phase mole fractions equals unity.
- $K_{i,j}$: Equilibrium ratio of component i at stage j.
- $x_{i,j}$: Mole fraction of component i in the liquid phase at stage j.

Equation 103: Secant method to iteratively update stage temperatures based on phase equilibrium constraints.

$$T_j^{(k+1)} = T_j^{(k)} - \frac{F(T_j^{(k)}) \cdot (T_j^{(k)} - T_j^{(k-1)})}{F\left(T_j^{(k)}\right) - F(T_j^{(k-1)})}$$

Where:

- $T_j^{(k+1)}$: Updated temperature for stage j at iteration $k + 1$.
- $T_j^{(k)}$: Current temperature estimates for stage j.
- $T_j^{(k-1)}$: Previous estimate for stage j.
- $F(T_j)$: Function that ensures the sum of vapor-phase mole fractions equals unity.

Equation 104: Heat duty at the condenser.

$$Q_c = Q_1 = F_1 \cdot H_1^F + V_2 \cdot H_2^F - W_1 \cdot H_1^V - (L_1 + U_1) \cdot H_1^L$$

Where:

- Q_c: Heat duty at the condenser.
- Q_1: Heat duty at stage 1 (condenser).
- F_1: Feed flow at stage 1.
- H_1^F: Enthalpy of the feed stream at stage 1.
- V_2: Vapor flow rate entering stage 1 from stage 2.
- H_2^V: Enthalpy of the vapor stream entering stage 1 from stage 2.
- W_1: Vapor side stream leaving stage 1.
- H_1^V: Enthalpy of the vapor stream leaving stage 1.
- L_1: Liquid flow rate leaving stage 1.
- U_1: Liquid side stream flow rate from stage 1.

- H_1^L: Enthalpy of the liquid stream leaving stage 1.

Equation 105: Heat duty at the condenser.

$$Q_R = Q_n = F_n \cdot H_n^F + L_{n-1} \cdot H_{n-1}^L - (V_n + W_n) \cdot H_n^V - U_n \cdot H_n^L$$

Where:

- Q_R: Heat duty at the reboiler.
- Q_n: Heat duty at stage n (reboiler).
- F_n: Feed flow at stage n.
- F_n: Feed flow rate to stage n.
- H_n^F: Enthalpy of the feed stream at stage n.
- L_{n-1}: Liquid flow rate entering stage n from stage $n-1$.
- H_{n-1}^L: Enthalpy of the liquid stream entering stage n.
- V_n: Vapor flow rate leaving stage n.
- W_n: Vapor side stream leaving stage n.
- H_n^V: Enthalpy of the vapor stream leaving stage n.
- U_n: Liquid side stream flow rate from stage n.
- H_n^L: Enthalpy of the liquid stream at stage n.

Equation 106: Vapor flow rate leaving stage n (reboiler).

$$V_n = F_n + L_{n-1} - U_n - W_n$$

Where:

- V_n: Vapor flow rate leaving stage n (reboiler).
- F_n: Feed flow rate to stage n.
- L_{n-1}: Liquid flow rate entering stage n from stage $n-1$.
- U_n: Liquid side stream flow rate from stage n.
- W_n: Vapor side stream flow rate from stage n.

Equation 107: Term used in the calculation of vapor flow rate stage j.

$$\gamma_j = \left(H_j^F - H_j^L\right) \cdot F_j + \left(H_{j-1}^L - H_j^L\right) \cdot \sum_{k=1}^{j-1}(F_k - U_k - W_k) + \left(H_j^L - H_j^V\right) \cdot W_j - Q_j$$

Where:

- γ_j: Term used in the calculation of vapor flow rate stage j.
- H_j^F: Enthalpy of the feed stream at stage j.
- H_j^L: Enthalpy of the liquid stream at stage j.
- F_j: Feed flow rate at stage j.
- H_{j-1}^L: Enthalpy of the liquid phase at stage $j-1$.
- $\sum_{k=1}^{j-1}(F_k - U_k - W_k)$: Summation term representing cumulative effect of feed and side streams up to stage $j-1$.
- H_j^V: Enthalpy of the vapor phase at stage j.

- W_j: Vapor side stream flow rate from stage j.
- Q_j: Heat duty at stage j.

Equation 108: Coefficient in the energy balance equation for calculating vapor flow rates.

$$\beta_j = H_{j+1}^V - H_j^L$$

Where:

- β_j: Coefficient in the energy balance equation for calculating vapor flow rates.
- H_{j+1}^V: Enthalpy of the vapor phase at stage $j + 1$.
- H_j^L: Enthalpy of the liquid phase at stage j.

Equation 109: Coefficient in the energy balance equation for calculating vapor flow rates.

$$\alpha_j = H_j^V - H_{j-1}^L$$

Where:

- α_j: Coefficient in the energy balance equation for calculating vapor flow rates.
- H_j^V: Enthalpy of the vapor phase at stage j.
- H_{j-1}^L: Enthalpy of the liquid phase at stage $j - 1$.

Equation 110: Side stream flow rate at stage j.

$$R_j = \frac{d_j}{d_j + 1} \cdot \left(F_j + V_{j+1} + V_{j-1}\right)$$

Where:

- R_j: Side stream flow rate at stage j.
- d_j: Ratio between the side stream and the stream leaving the stage.
- F_j: Feed flow rate to stage j.
- V_{j+1}: Vapor flow rate entering stage j from stage $j + 1$.
- L_{j-1}: Liquid flow rate entering stage j from stage $j - 1$.

Equation 111: Vapor side stream flow rate at stage j.

$$W_j = \omega_j \cdot R_j$$

Where:

- W_j: Vapor side stream flow rate at stage j.
- ω_j: Vapor fraction of the side stream at stage j.
- R_j: Total side stream flow rate at stage j.

Equation 112: Vapor side stream flow rate at stage j.

$$U_j = (1 - \omega_j) \cdot R_j$$

Where:

- U_j: Liquid side stream flow rate at stage j.
- ω_j: Vapor fraction of the side stream at stage j.
- R_j: Total side stream flow rate at stage j.

Equation 113: Liquid flow rate at stage j.

$$L_j = F_j + V_{j+1} - V_j + L_{j-1} - R_j$$

Where:

- L_j: Liquid flow rate at stage j.
- F_j: Feed flow rate entering stage j.
- V_{j+1}: Vapor flow rate entering stage j from stage $j + 1$.
- V_j: Vapor flow rate leaving stage j.
- L_{j-1}: Liquid flow rate entering stage j from stage $j - 1$.
- R_j: Total side stream flow rate at stage j.

Equation 114: Convergence criterion for the iterative solution of the steady-state distillation model.

$$\sum_{j=1}^{n}\left(\frac{T_j^{(k)} - T_j^{(k-1)}}{T_j^{(k)}}\right)^2 + \sum_{j=1}^{n-1}\left(\frac{L_j^{(k)} - L_j^{(k-1)}}{L_j^{(k)}}\right)^2 + \sum_{j=2}^{n}\left(\frac{V_j^{(k)} - V_j^{(k-1)}}{V_j^{(k)}}\right)^2 \leq \mathrm{tolerance}$$

Where:

- T_j: Temperature at stage j.
- L_j: Liquid flow at stage j.
- V_j: Vapor flow at stage j.
- k: Iteration step in the numerical solution.
- $\mathrm{tolerance}$: convergence criterion for stopping the iterative process.

PSA[5–7]

Equation 115: Mass balance for PSA.

$$\frac{\partial C_i}{\partial t} = -\frac{\partial (u \cdot C_i)}{\partial t} + D_{is} \cdot \frac{\partial^2 C_i}{\partial z^2} - \rho_s \cdot \frac{(1-\varepsilon)}{\varepsilon} \cdot \frac{\partial q_i}{\partial z_i}$$

Where:

- C_i: Concentration of component i (mol·m⁻³).
- t: Time (s).
- D_{is}: Dispersion coefficient ($m^2 \cdot s^{-1}$).
- z: Axial coordinate (m).
- ε: Bed void fraction ($m^3 \cdot m^{-3}$).
- ρ_s: Density of solid ($kg \cdot m^{-3}$).
- q_i: Uptake of component i ($mol \cdot kg^{-1}$).

- u: Superficial velocity of the gas in the axial direction (m·s⁻¹).

Equation 116: Momentum balance for PSA.

$$-\frac{\partial P}{\partial z} = \frac{180 \cdot \mu}{d_p^2} \cdot \frac{(1-\varepsilon)^2}{\varepsilon^3} \cdot u + \frac{7}{4} \cdot \frac{\rho_f}{d_p} \cdot \frac{(1-\varepsilon)^2}{\varepsilon^3} \cdot u \cdot |u|$$

Where:

- $\frac{\partial P}{\partial z}$: Pressure drop gradient (Pa·m⁻¹).
- μ: Fluid viscosity (kg·m⁻¹·s⁻¹).
- d_p: Particle diameter (m).
- ε: Bed void fraction (m³·m⁻³).
- u: Superficial velocity of the gas in the axial direction (m·s⁻¹).
- ρ_f: Density of solid (kg·m⁻³).

Equation 117: Gas phase energy balance under boundary conditions for PSA.

$$\left(\sum_{i=1}^{n} C_i \cdot C_{p,i}\right) \cdot \frac{\partial T_g}{\partial t} = h_{heat} \cdot a_{surf} \cdot (T_s - T_g) - \left(\sum_{i=1}^{n} u \cdot C_i \cdot C_{p,i}\right) \cdot \frac{\partial T_g}{\partial z}$$

Equation 118: Solid phase energy balance under boundary conditions for PSA.

$$(1-\varepsilon) \cdot \rho_s \left(\sum_{i=1}^{n} q_i \cdot C_{p,i} + C_{p,s}\right) \cdot \frac{\partial T_s}{\partial t} = h_{heat} \cdot a_{surf} \cdot (T_g - T_s) + \lambda_{cond} \cdot \frac{\partial^2 T_s}{\partial z^2} + \sum_{i=1}^{n} (-\Delta H_{ad,i}) \cdot \rho_s \cdot (1-\varepsilon) \cdot \frac{\partial q_i}{\partial t}$$

Where (for Equation 117 and Equation 118):

- T_g: Gas temperature (K).
- T_s: Solid temperature (K).
- t: Time (s).
- q_i: Adsorbed amount of component i (mol·kg⁻¹).
- C_i: Gas-phase concentration of component i (mol·m⁻³).
- $C_{p,i}$: Molar heat capacity of adsorbed component i (J·mol⁻¹·K⁻¹).
- $C_{p,s}$: Specific heat capacity of the solid adsorbent (J·kg⁻¹·K⁻¹).
- z: Axial coordinate (m).
- ε: Bed void fraction (m³·m⁻³).
- h_{heat}: Gas-solid heat transfer coefficient (W·m⁻²·K⁻¹).
- a_{surf}: Gas-solid interfacial area per unit bed volume (m²·m⁻³).
- d_p: Particle diameter (m).
- ρ_s: Particle density of the solid adsorbent (kg·m⁻³).
- λ_{cond}: Effective axial thermal conductivity (W·m⁻¹·K⁻¹).
- $\Delta H_{ad,i}$: Heat of adsorption of component i (J·mol⁻¹).
- n: Total number of adsorbed components (unitless).

Equation 119: Extended Langmuir isotherm for PSA.

$$q_i = \frac{q_{m,i} \cdot b_i \cdot P_i}{1 + \sum_{j=1}^{n} b_j \cdot P_j}$$

Where (for Equation 117 and Equation 118):

- q_i: Uptake of component i (mol·kg^{-1}).
- $q_{m,i}$: Maximum adsorption capacity of component i (mol·kg^{-1}).
- b_i: Langmuir affinity constant of component i (Pa^{-1}).
- P_i: Partial pressure of component i (Pa).
- b_j: Langmuir affinity constant of component j (Pa^{-1}).
- P_j: Partial pressure of component j (Pa).
- n: Total number of components in the gas mixture (unitless).

III. Technical Results/Heat Integration

Overview of the hot and cold process streams used for pinch analysis, together with a summary of the heat exchanger network (HEN) results, including heat recovery above and below the pinch point and the corresponding heating and cooling utility requirements for the decentralized and centralized process configurations.

Table S4: Overview of hot and cold streams for pinch analysis (decentralised vs. centralised configuration).

aa	Code	Block	Q (kW)	mCp (kW/°C)	Tin (°C)	Tout (°C)	Type
Decentralised							
1	HEX-Q101	CST	-822.07	7.45	232.31	122.00	HOT
2	HEX-Q102	CST	-896.87	16.78	78.44	25.00	HOT
3	HEX-C1	CST	-142.41	2.95	93.20	45.00	HOT
4	HEX-C2	CST	-131.11	3.10	87.26	45.00	HOT
5	HEX-C3	CST	-79.69	2.38	78.48	45.00	HOT
6	HEX-C4	CST	-92.59	2.49	82.21	45.00	HOT
7	HEX-C5	CST	-101.53	2.47	86.03	45.00	HOT
8	HEX-201	CST	-237.14	3.39	45.00	-25.00	HOT
9	HEX-202	CST	-43.65	1.50	-25.00	-54.00	HOT
10	HEX-203	CST	-46.23	1.19	-54.00	-93.00	HOT
11	T-201-C	CST	-53.57	107.15	-98.13	-98.63	HOT
12	HEX-BC2	CST	-26.09	0.52	80.00	30.00	HOT
13	T-202-C	CST	-80.79	161.58	-17.54	-18.04	HOT
14	T-203-C	CST	-352.75	705.49	-20.09	-20.59	HOT
15	T-204-C	CST	-50.87	101.75	55.68	55.18	HOT
16	HEX-BR2	CST	-68.44	1.48	55.18	9.00	HOT
17	HEX-BC3	CST	-10.33	0.52	50.00	30.00	HOT
18	T-205-C	CST	-35.79	71.57	51.98	51.48	HOT
19	HEX-DEBT	CST	-40.75	1.54	51.48	25.00	HOT
20	HEX-DEBB	CST	-3.05	0.05	92.77	25.00	HOT

21	T-206-C	CST	-748.14	1496.28	51.48	50.98	HOT
22	HEX-C3T	CST	-56.47	2.17	50.98	25.00	HOT
23	HEX-C3B	CST	-2.13	0.04	80.87	25.00	HOT
24	HEX-DECA	CST	151.29	1.55	67.40	164.90	COLD
25	HEX-DECB	CST	754.85	1509.69	164.90	165.40	COLD
26	HEX-DECC	CST	30.14	0.87	165.40	200.00	COLD
27	PRE-HEAT	PRE-HEATING	1134.465	3.88	207.80	500.00	COLD
28	PY-OIL-VAP	PYROLYSER	489.75	2.39	30.00	235.00	COLD
29	HEX-DELW	RDR	4.68	0.035221	67.00	200.00	COLD
30	HEX-DEMT	CST	45.58	0.37	-98.63	25.00	COLD
31	T-201-R	CST	146.60	293.19	22.44	22.94	COLD
32	HEX-BR1	CST	43.57	0.54	-18.04	63.00	COLD
33	T-202-R	CST	169.55	339.11	77.72	78.22	COLD
34	HEX-C2T	CST	21.85	0.48	-20.59	25.00	COLD
35	HEX-C2B	CST	11.42	0.46	0.31	25.00	COLD
36	T-203-R	CST	316.79	633.58	-0.19	0.31	COLD
37	T-204-R	CST	100.03	200.05	120.15	120.65	COLD
38	T-205-R	CST	53.76	107.52	92.27	92.77	COLD
39	T-206-R	CST	804.39	1608.78	80.37	80.87	COLD
**Centralised**							
1	HEX-Q101	CST	-822.07	7.45	232.31	122.00	HOT
2	HEX-Q102	CST	-896.87	16.78	78.44	25.00	HOT
3	HEX-C1	CST	-76.37	2.94	71.02	45.00	HOT
4	HEX-C2	CST	-70.83	3.73	64.01	45.00	HOT
5	HEX-C3	CST	-177.59	6.99	70.41	45.00	HOT
6	HEX-C4	CST	-206.21	6.00	79.38	45.00	HOT
7	HEX-C5	CST	-157.23	4.02	84.07	45.00	HOT
8	HEX-201	CST	-298.49	4.26	45.00	-25.00	HOT
9	HEX-202	CST	-38.75	1.34	-25.00	-54.00	HOT
10	HEX-203	CST	-43.36	1.11	-54.00	-93.00	HOT
11	T-201-C	CST	-54.90	109.79	-98.58	-99.08	HOT
12	HEX-BC2	CST	-28.95	0.58	80.00	30.00	HOT
13	T-202-C	CST	-93.24	186.49	-14.28	-14.78	HOT
14	T-203-C	CST	-369.76	739.53	-20.09	-20.59	HOT
15	T-204-C	CST	-65.73	131.46	56.85	56.35	HOT
16	HEX-BR2	CST	-93.48	1.86	56.35	6.00	HOT
17	HEX-BC3	CST	-13.25	0.66	50.00	30.00	HOT
18	T-205-C	CST	-55.79	111.58	50.32	49.82	HOT
19	HEX-DEBT	CST	-63.32	2.55	49.82	25.00	HOT
20	HEX-DEBB	CST	-1.21	0.02	90.63	25.00	HOT
21	T-206-C	CST	-1036.88	2073.76	51.47	50.97	HOT
22	HEX-C3T	CST	-70.01	2.70	50.97	25.00	HOT
23	HEX-C3B	CST	-3.86	0.07	79.24	25.00	HOT
24	PYROGAS	PYROLYSER	-168.28	0.42	430.00	25.00	HOT

25	HEX-DECA	CST	161.44	1.53	59.64	164.90	COLD
26	HEX-DECB	CST	746.89	1493.79	164.90	165.40	COLD
27	HEX-DECC	CST	29.83	0.86	165.40	200.00	COLD
28	PRE-HEAT	PRE-HEATING	1134.465	3.88	207.80	500.00	COLD
29	PY-OIL-VAP	PYROLYSER	489.75	2.39	30.00	235.00	COLD
30	HEX-DELW	RDR	5.34	0.038130	60.00	200.00	COLD
31	HEX-DEMT	CST	46.87	0.38	-99.08	25.00	COLD
32	T-201-R	CST	171.44	342.87	28.18	28.68	COLD
33	HEX-BR1	CST	43.40	0.60	-14.78	58.00	COLD
34	T-202-R	CST	193.42	386.84	78.57	79.07	COLD
35	HEX-C2T	CST	22.91	0.50	-20.59	25.00	COLD
36	HEX-C2B	CST	16.47	0.87	6.02	25.00	COLD
37	T-203-R	CST	327.31	654.61	5.52	6.02	COLD
38	T-204-R	CST	127.00	254.01	112.64	113.14	COLD
39	T-205-R	CST	86.29	172.59	90.13	90.63	COLD
40	T-206-R	CST	1107.72	2215.45	78.74	79.24	COLD

Table S5: Quantification of the results presented in HEN (decentralised vs. centralised configuration).

aa	Code	mCp (kW/°C)	Type	Tin (°C)	Tout (°C)	ΔT	Q (kW)	Heaters (kW)	Type	Tin (°C)	Tout (°C)	ΔT	Q (kW)	Coolers (kW)
**Decentralised**														
			**Above pinch**						**Below pinch**					
1	HEX-Q101	7.5	HOT	232.3	122.0	110.3	822.1							
2	HEX-Q102	16.8							HOT	78.4	25.0	53.4	896.9	763.9
3	HEX-C1	3.0	HOT	93.2	87.4	5.8	17.1		HOT	87.4	45.0	48.2	125.3	125.3
4	HEX-C2	3.1							HOT	87.3	45.0	42.3	131.1	131.1
5	HEX-C3	2.4							HOT	78.5	45.0	33.5	79.7	79.7
6	HEX-C4	2.5							HOT	82.2	45.0	37.2	92.6	92.6
7	HEX-C5	2.5							HOT	86.0	45.0	41.0	101.5	101.5
8	HEX-201	3.4							HOT	45.0	-25.0	70.0	237.1	237.1
9	HEX-202	1.5							HOT	-25.0	-54.0	29.0	43.6	43.6
10	HEX-203	1.2							HOT	-54.0	-93.0	39.0	46.2	46.2
11	T-201-C	107.1							HOT	-98.1	-98.6	0.5	53.6	53.6
12	HEX-BC2	0.5							HOT	80.0	30.0	50.0	26.1	26.1
13	T-202-C	161.6							HOT	-17.5	-18.0	0.5	80.8	80.8
14	T-203-C	705.5							HOT	-20.1	-20.6	0.5	352.7	352.7
15	T-204-C	101.7							HOT	55.7	55.2	0.5	50.9	50.9
16	HEX-BR2	1.5							HOT	55.2	9.0	46.2	68.4	68.4
17	HEX-BC3	0.5							HOT	50.0	30.0	20.0	10.3	10.3
18	T-205-C	71.6							HOT	52.0	51.5	0.5	35.8	35.8
19	HEX-DEBT	1.5							HOT	51.5	25.0	26.5	40.7	40.7
20	HEX-DEBB	0.0	HOT	92.8	87.4	5.4	0.2		HOT	87.4	25.0	67.8	2.8	2.8
21	T-206-C	1496.3							HOT	51.5	51.0	0.5	748.1	205.9
22	HEX-C3T	2.2							HOT	51.0	25.0	26.0	56.5	56.5
23	HEX-C3B	0.04							HOT	80.9	25.0	55.9	2.1	2.1

24	HEX-DECA	1.6	COLD	67.4	164.9	97.5	151.3	151.3						
25	HEX-DECB	1509.7	COLD	164.9	165.4	0.5	754.8	737.2						
26	HEX-DECC	0.9	COLD	165.4	200.0	34.6	30.1	30.1						
27	PRE-HEAT	3.9	COLD	207.8	500.0	292.2	1134.5	1134.5						
28	PY-OIL-VAP	2.4	COLD	67.4	235.0	167.6	400.4	400.4	COLD	30.0	67.4	37.4	89.3	
29	HEX-DELW	0.04	COLD	67.4	200.0	132.6	4.7	4.7	COLD	67.0	67.4	0.4	0.0	
30	HEX-DEMT	0.4							COLD	-98.6	25.0	123.6	45.6	
31	T-201-R	293.2							COLD	22.4	22.9	0.5	146.6	
32	HEX-BR1	0.5							COLD	-18.0	63.0	81.0	43.6	
33	T-202-R	339.1	COLD	77.7	78.2	0.5	169.6	152.2						
34	HEX-C2T	0.5							COLD	-20.6	25.0	45.6	21.9	
35	HEX-C2B	0.5							COLD	0.3	25.0	24.7	11.4	
36	T-203-R	633.6							COLD	-0.2	0.3	0.5	316.8	
37	T-204-R	200.1	COLD	120.1	120.6	0.5	100.0	100.0						
38	T-205-R	107.5	COLD	92.3	92.8	0.5	53.8	53.8						
39	T-206-R	1608.8	COLD	80.4	80.9	0.5	804.4							
**Total**								**2664.4**	**Total**					**2607.8**
**Centralised**														
			**Above pinch**						**Below pinch**					
1	HEX-Q101	7.5	HOT	232.3	122.0	110.3	822.1							
2	HEX-Q102	16.8							HOT	78.43862	25.0	53.4	896.9	765.1
3	HEX-C1	2.9							HOT	71.01523	45.0	26.0	76.4	76.4
4	HEX-C2	3.7							HOT	64.01401	45.0	19.0	70.8	70.8
5	HEX-C3	7.0							HOT	70.41371	45.0	25.4	177.6	177.6
6	HEX-C4	6.0							HOT	79.38226	45.0	34.4	206.2	206.2
7	HEX-C5	4.0							HOT	84.07336	45.0	39.1	157.2	157.2
8	HEX-201	4.3							HOT	45.00007	-25.0	70.0	298.5	298.5
9	HEX-202	1.3							HOT	-25.0004	-54.0	29.0	38.7	38.7
10	HEX-203	1.1							HOT	-54.0001	-93.0	39.0	43.4	43.4
11	T-201-C	109.8							HOT	-98.5779	-99.1	0.5	54.9	54.9
12	HEX-BC2	0.6							HOT	80	30.0	50.0	28.9	28.9
13	T-202-C	186.5							HOT	-14.2824	-14.8	0.5	93.2	93.2
14	T-203-C	739.5							HOT	-20.091	-20.6	0.5	369.8	369.8
15	T-204-C	131.5							HOT	56.85126	56.4	0.5	65.7	65.7
16	HEX-BR2	1.9							HOT	56.35126	6.0	50.4	93.5	93.5
17	HEX-BC3	0.7							HOT	50	30.0	20.0	13.2	13.2
18	T-205-C	111.6							HOT	50.32184	49.8	0.5	55.8	55.8
19	HEX-DEBT	2.6							HOT	49.82184	25.0	24.8	63.3	63.3
20	HEX-DEBB	0.0	HOT	90.6	84.1	6.6	0.1		HOT	84.07336	25.0	59.1	1.1	1.1
21	T-206-C	2073.8							HOT	51.46934	51.0	0.5	1036.9	451.9
22	HEX-C3T	2.7							HOT	50.96934	25.0	26.0	70.0	70.0
23	HEX-C3B	0.1							HOT	79.23681	25.0	54.2	3.9	3.9
24	PYROGAS	0.4	HOT	430.0	84.1	345.9	143.7		HOT	84.07336	25.0	59.1	24.5	24.5

25	HEX-DECA	1.5	COLD	64.1	164.9	100.8	154.6	154.6	COLD	59.6435	64.1	4.4	6.8	
26	HEX-DECB	1493.8	COLD	164.9	165.4	0.5	746.9	746.9						
27	HEX-DECC	0.9	COLD	165.4	200.0	34.6	29.8	29.8						
28	PRE-HEAT	3.9	COLD	207.8	500.0	292.2	1134.5	1134.5						
29	PY-OIL-VAP	2.4	COLD	64.1	235.0	170.9	408.3	408.3	COLD	30	64.1	34.1	81.4	
30	HEX-DELW	0.0	COLD	64.1	200.0	135.9	5.2	5.2	COLD	60	64.1	4.1	0.2	
31	HEX-DEMT	0.4							COLD	-99.0779	25.0	124.1	46.9	
32	T-201-R	342.9							COLD	28.18474	28.7	0.5	171.4	
33	HEX-BR1	0.6							COLD	-14.7824	58.0	72.8	43.4	
34	T-202-R	386.8	COLD	78.6	79.1	0.5	193.4	193.4						
35	HEX-C2T	0.5							COLD	-20.591	25.0	45.6	22.9	
36	HEX-C2B	0.9							COLD	6.024532	25.0	19.0	16.5	
37	T-203-R	654.6							COLD	5.524532	6.0	0.5	327.3	
38	T-204-R	254.0	COLD	112.6	113.1	0.5	127.0	127.0						
39	T-205-R	172.6	COLD	90.1	90.6	0.5	86.3	86.3						
40	T-206-R	2215.4	COLD	78.7	79.2	0.5	1107.7	141.8						
**Total**								**3027.9**						**3223.8**

IV. Technical Results/System Boundaries, Mass & Energy Balances

Overview of the system boundaries and process-level mass, energy, and greenhouse gas (GHG) emission contributions for the decentralized and centralized process configurations.

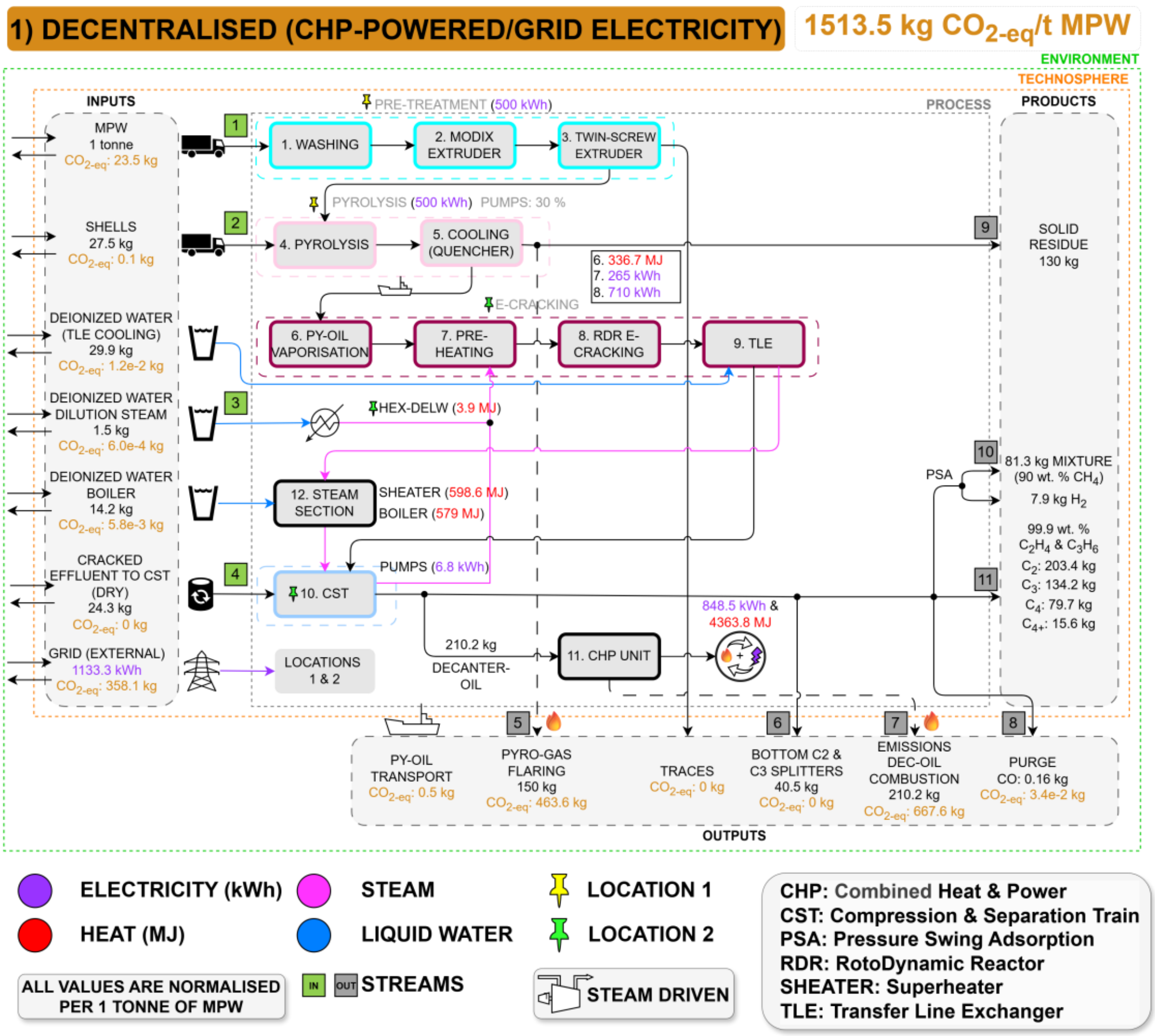

Figure S7: *System boundaries and process-level mass, energy, and greenhouse gas (GHG) emission contributions for the decentralised CHP-powered/grid-electricity configuration (Scenario 1). The flow diagram illustrates the main process units, material streams, utility integration, and major emission sources across the MPW-to-olefins value chain. The dark-grey dashed line defines the foreground process boundary, the orange boundary represents background technosphere inputs, and the green boundary denotes the surrounding environment associated with direct (Scope 1) and indirect (Scope 2–3) emissions. Key process sections include pre-treatment, pyrolysis, e-cracking, the steam section, and the compression and separation train (CST). In the decentralised configuration, yellow and green pins indicate Location 1 (pre-treatment and pyrolysis) and Location 2 (e-cracking and CST), respectively. In the centralised configuration, all process units are co-located at a single site and are therefore represented by the green pin only. All values are normalised per tonne of mixed plastic waste (MPW) processed. The colour coding, symbols, and process section classification are consistent with those presented in Figure 1.*

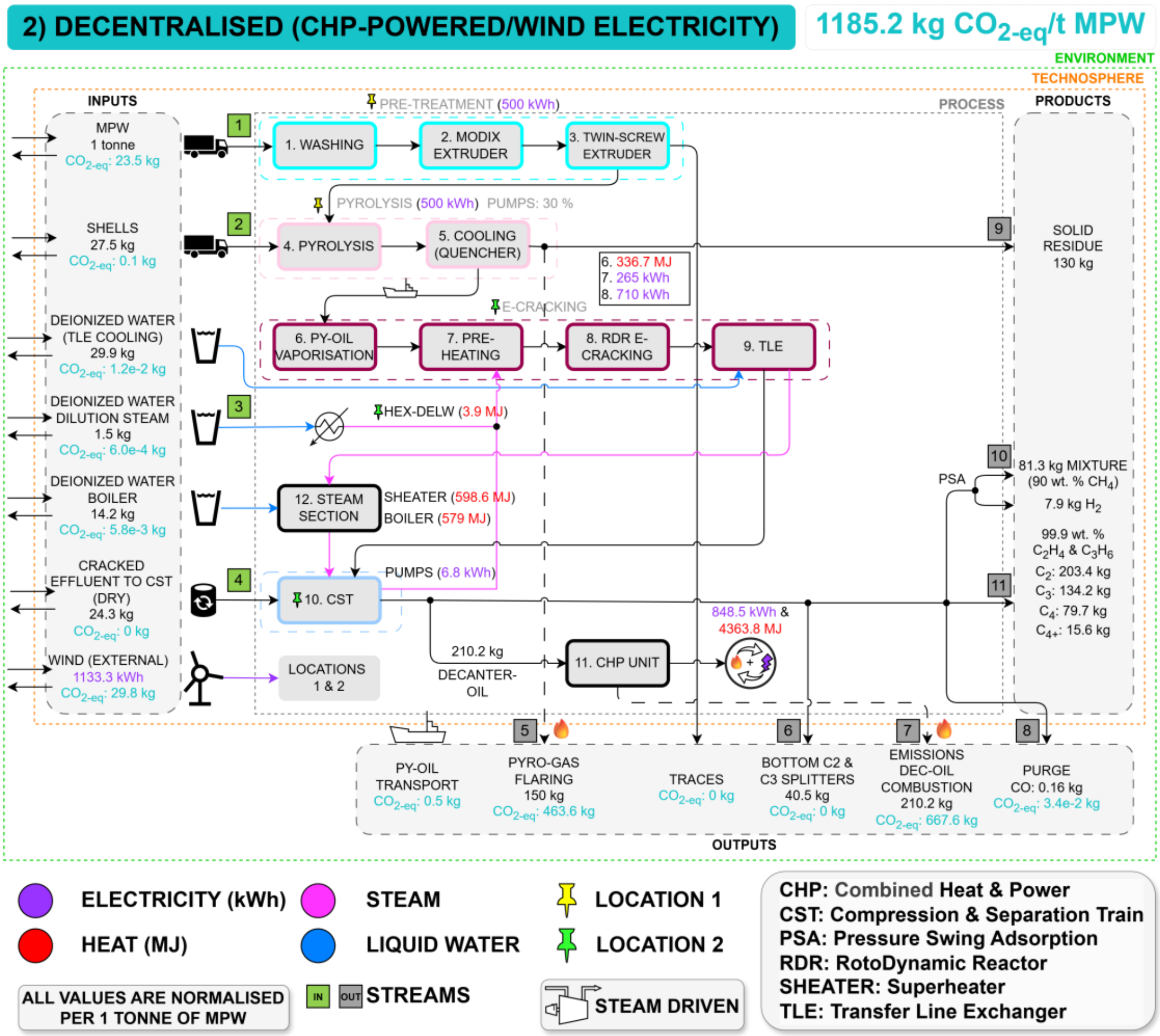

Figure S8: System boundaries and process-level energy, mass, and emission breakdown for the decentralised CHP-powered configuration supplied with wind electricity (Scenario 2). The figure follows the same structure, boundaries, and normalisation basis as Figure S1.

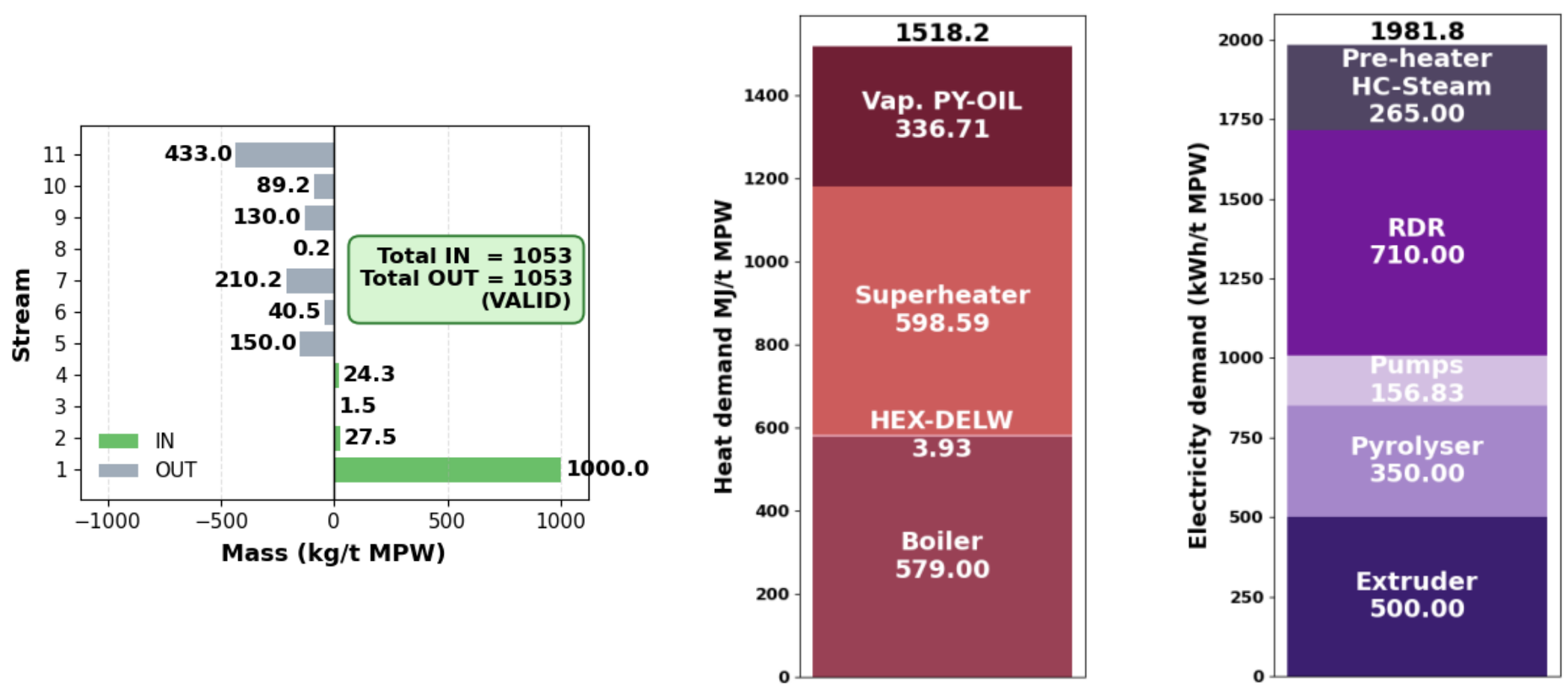

Figure S9: Mass and energy balances for the decentralised CHP-powered configurations (Scenarios 1–2). The left panel presents the overall mass balance distribution across process inputs and outputs, while the middle and right panels show the corresponding heat and electricity demands of the main process units. All values are normalised per tonne of mixed plastic waste (MPW) processed.

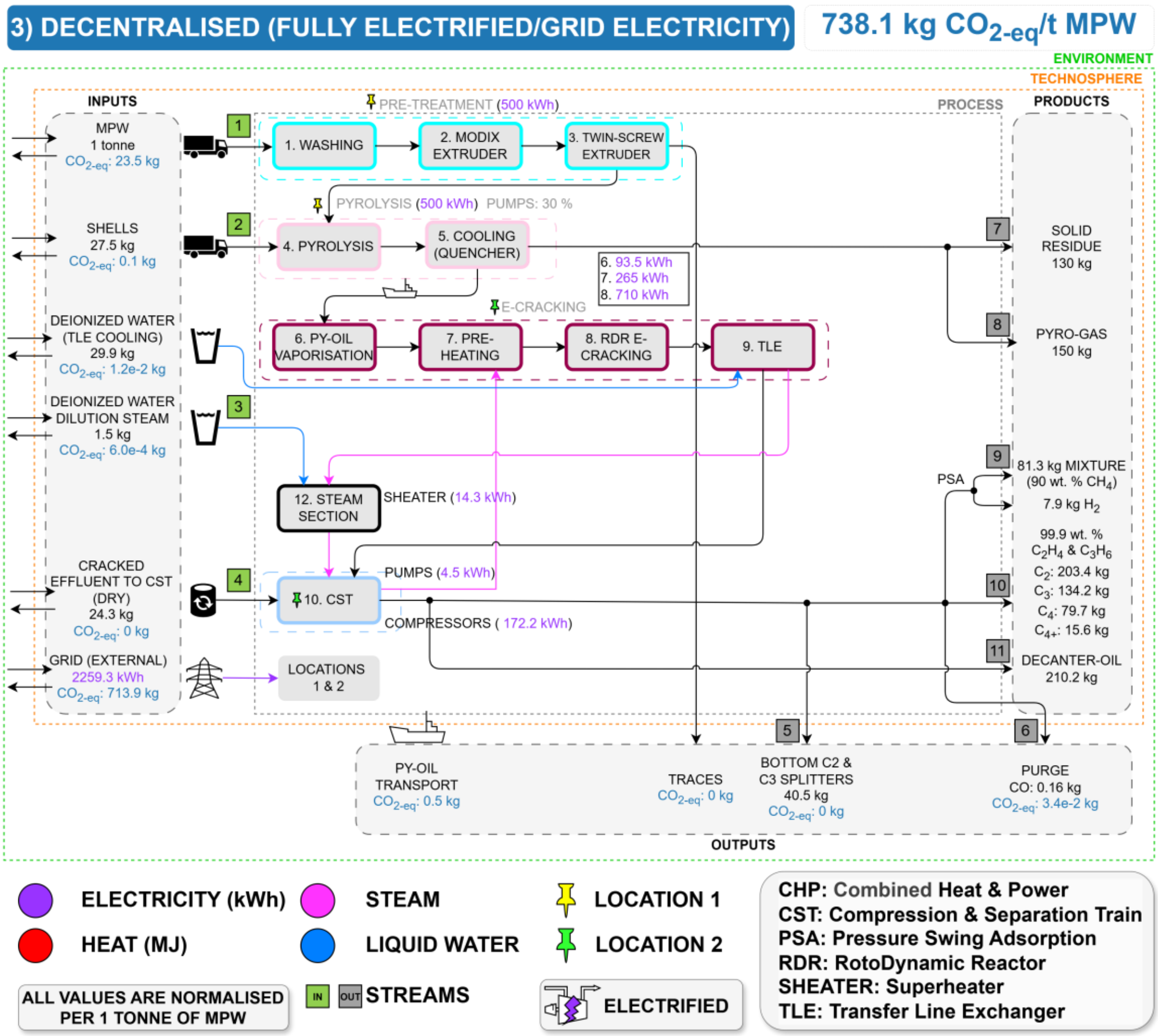

Figure S10: System boundaries and process-level energy, mass, and emission breakdown for the decentralised fully electrified configuration supplied with grid electricity (Scenario 3). The figure follows the same structure, boundaries, and normalisation basis as Figure S1.

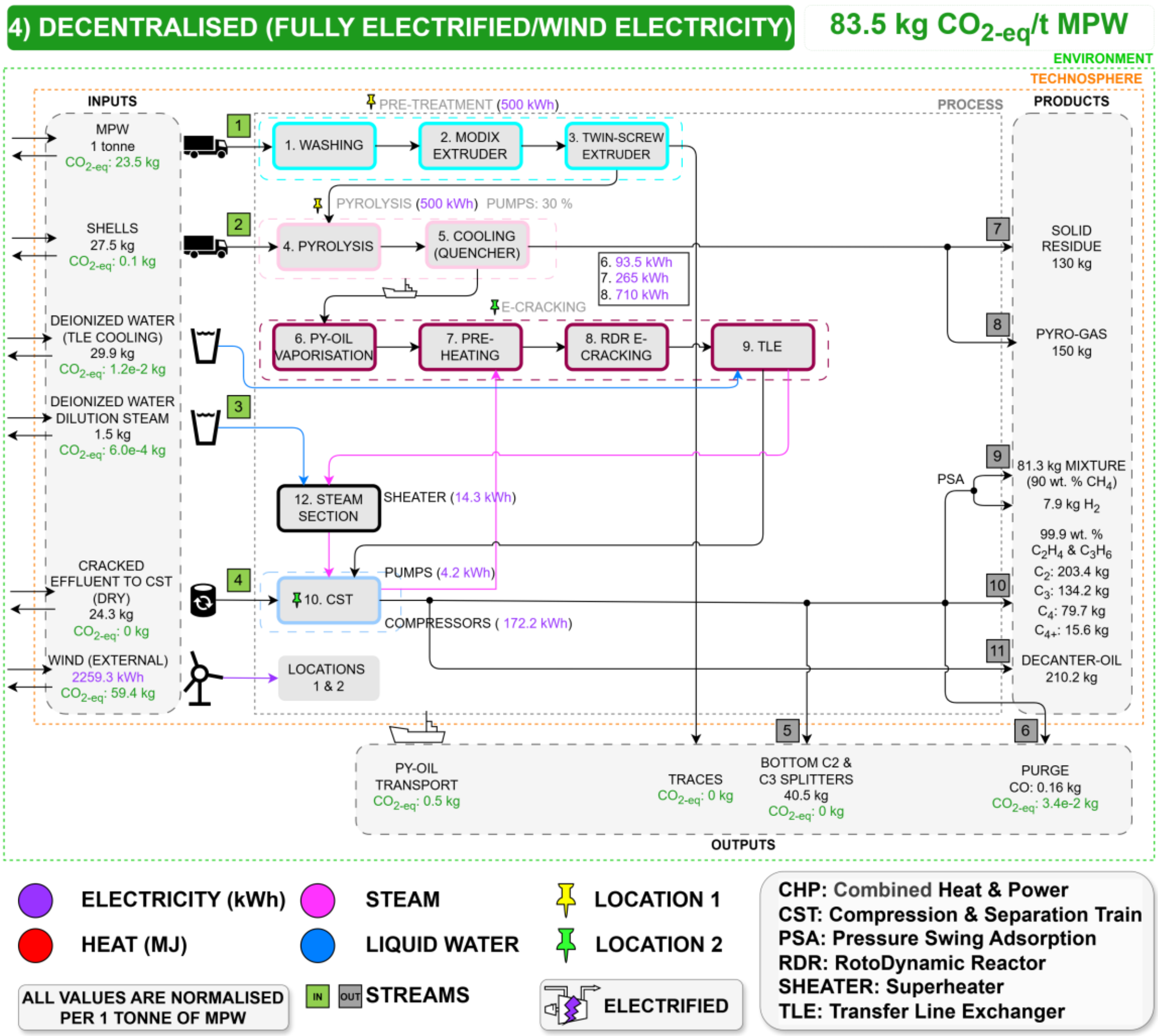

Figure S11: System boundaries and process-level energy, mass, and emission breakdown for the decentralised fully electrified configuration supplied with wind electricity (Scenario 4). The figure follows the same structure, boundaries, and normalisation basis as Figure S1.

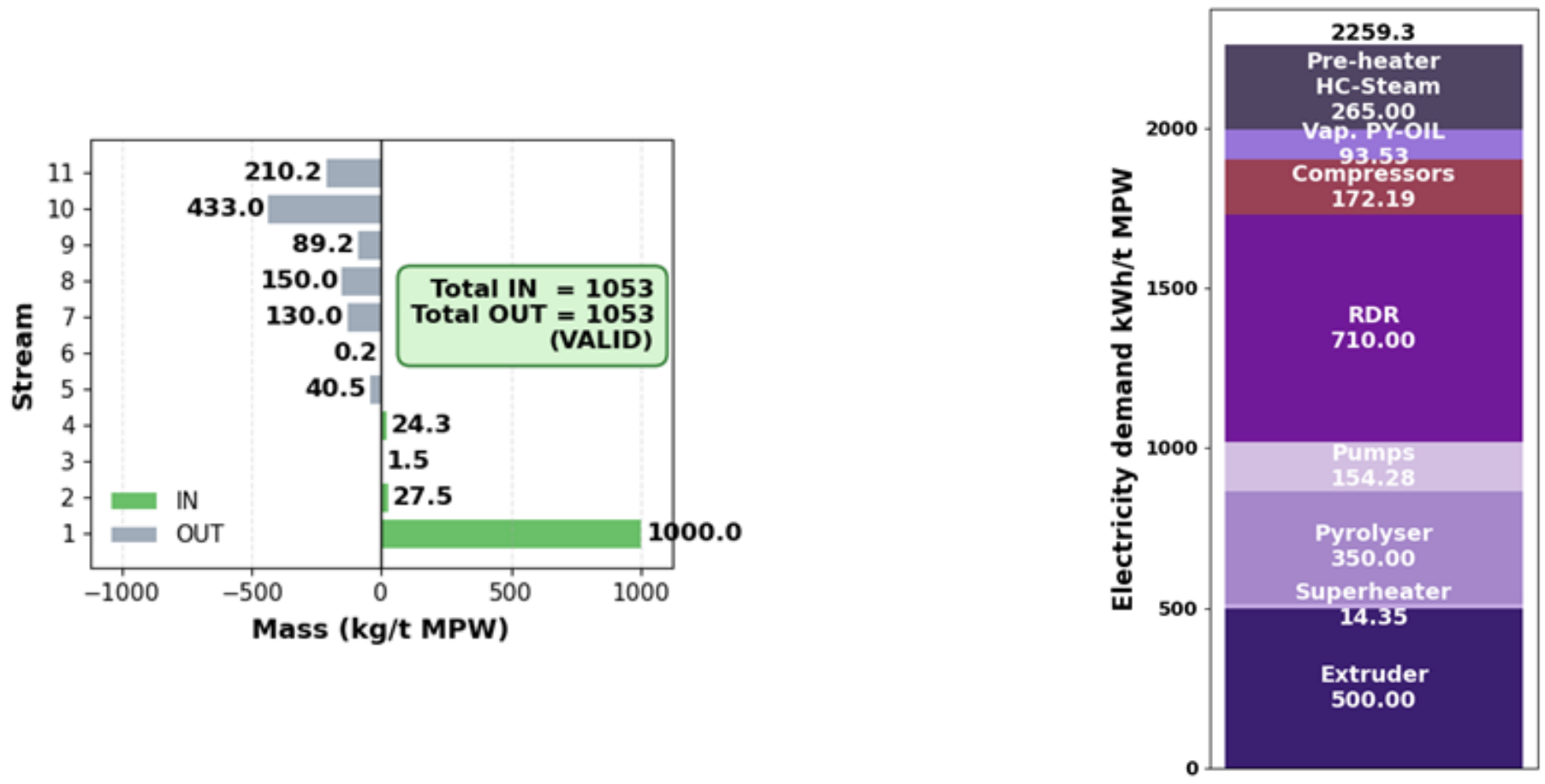

Figure S12: Mass and energy balances for the decentralised fully electrified configurations (Scenarios 3–4). The left panel presents the overall mass balance distribution across process inputs and outputs, while the right panel shows the corresponding electricity demands of the main process units. In the fully electrified configuration, combustion-based heat generation is eliminated and all major process utilities are electrically driven.

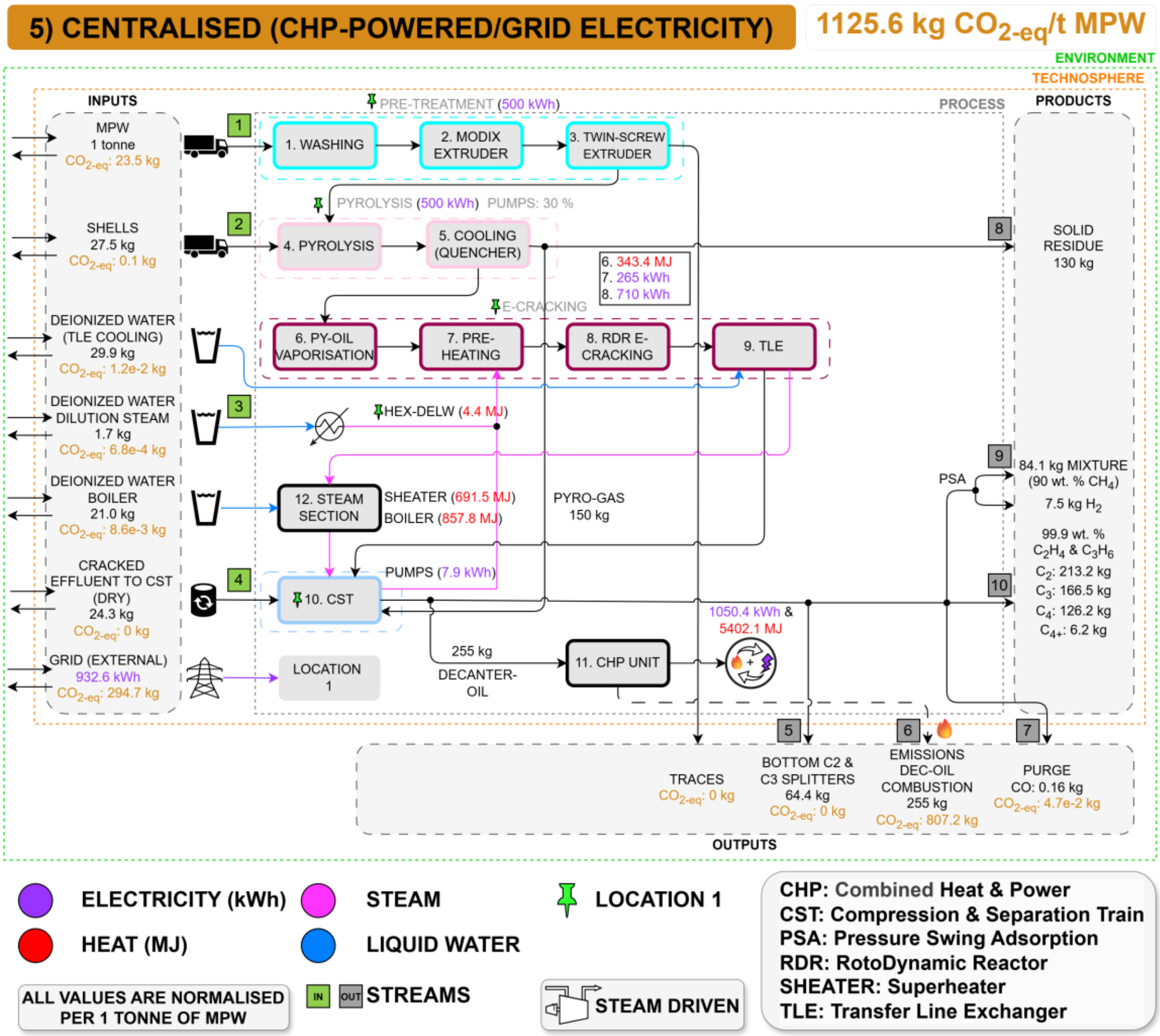

Figure S13: System boundaries and process-level energy, mass, and emission breakdown for the centralised CHP-powered configuration supplied with grid electricity (Scenario 5). The figure follows the same structure, boundaries, and normalisation basis as Figure S1.

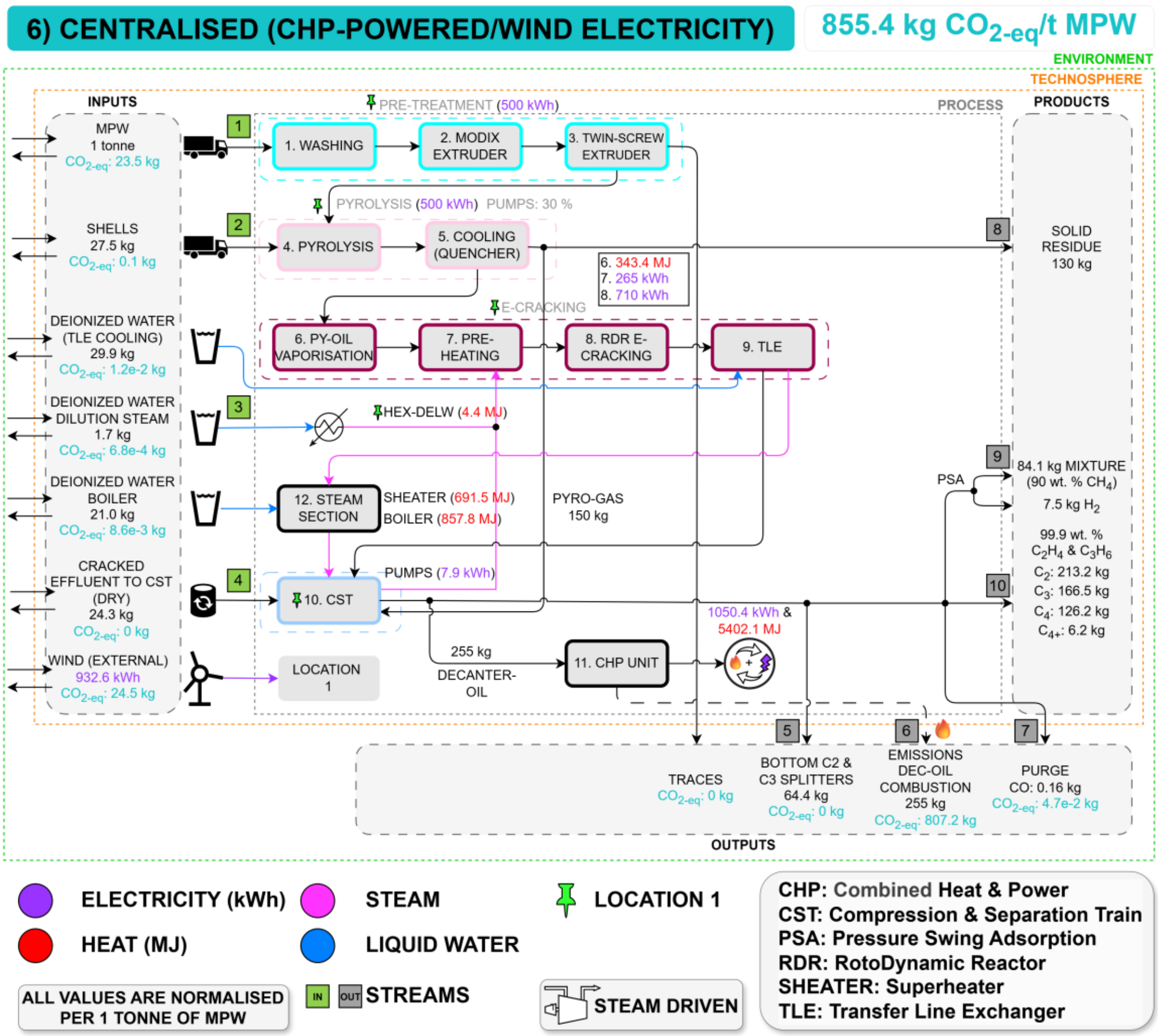

Figure S14: System boundaries and process-level energy, mass, and emission breakdown for the centralised CHP-powered configuration supplied with wind electricity (Scenario 6). The figure follows the same structure, boundaries, and normalisation basis as Figure S1.

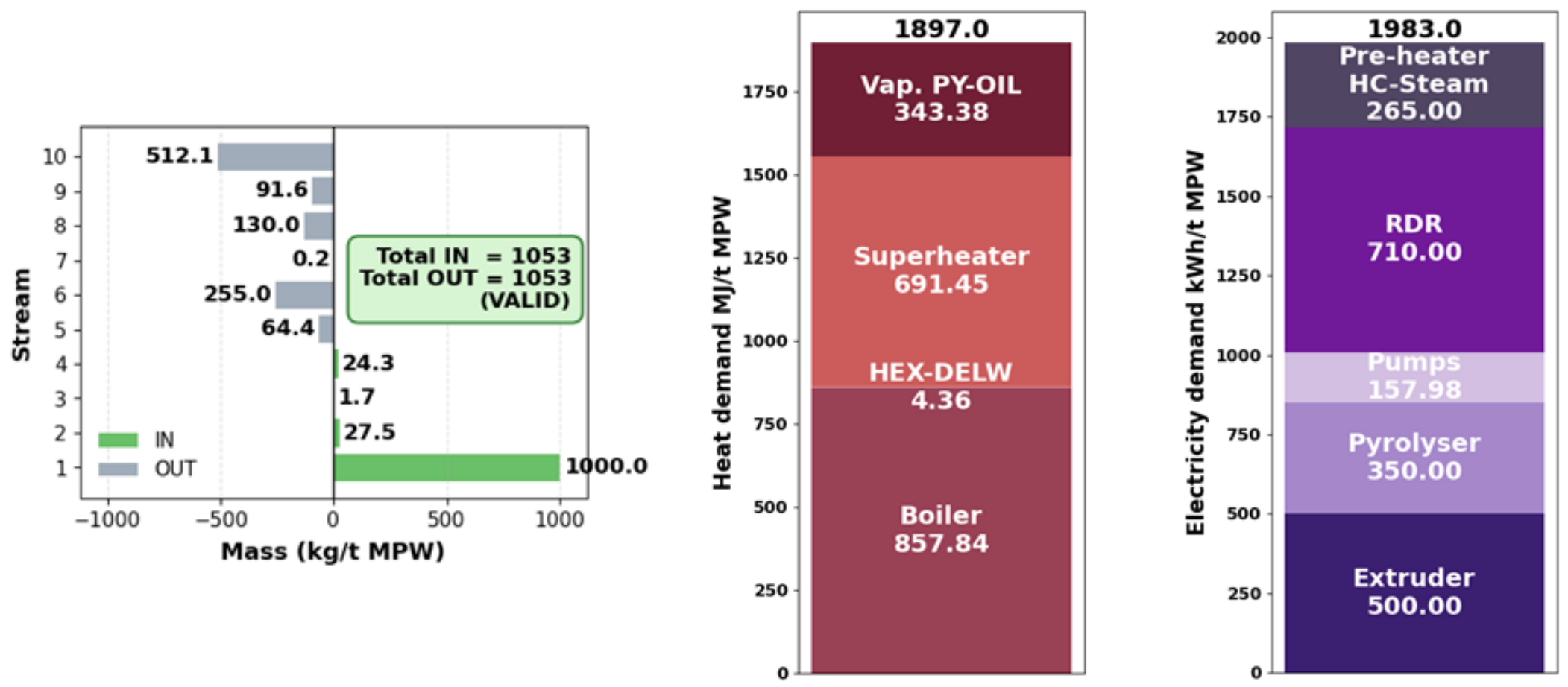

Figure S15: Mass and energy balances for the centralised CHP-powered configurations (Scenarios 5–6). The left panel presents the overall mass balance distribution across process inputs and outputs, while the middle and right panels show the corresponding heat and electricity demands of the main process units. All values are normalised per tonne of mixed plastic waste (MPW) processed.

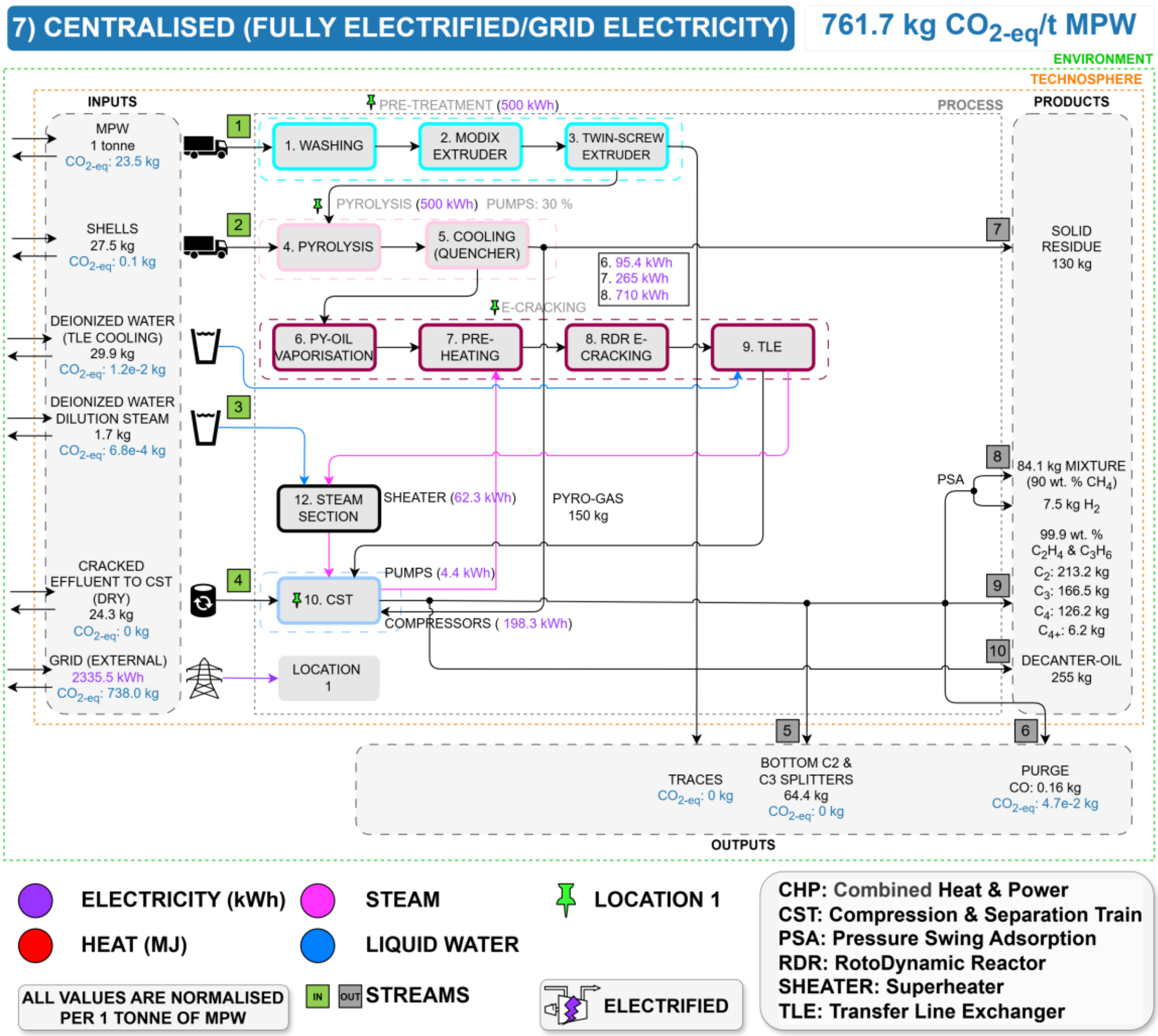

Figure S16: System boundaries and process-level energy, mass, and emission breakdown for the centralised fully electrified configuration supplied with grid electricity (Scenario 7). The figure follows the same structure, boundaries, and normalisation basis as Figure S1.

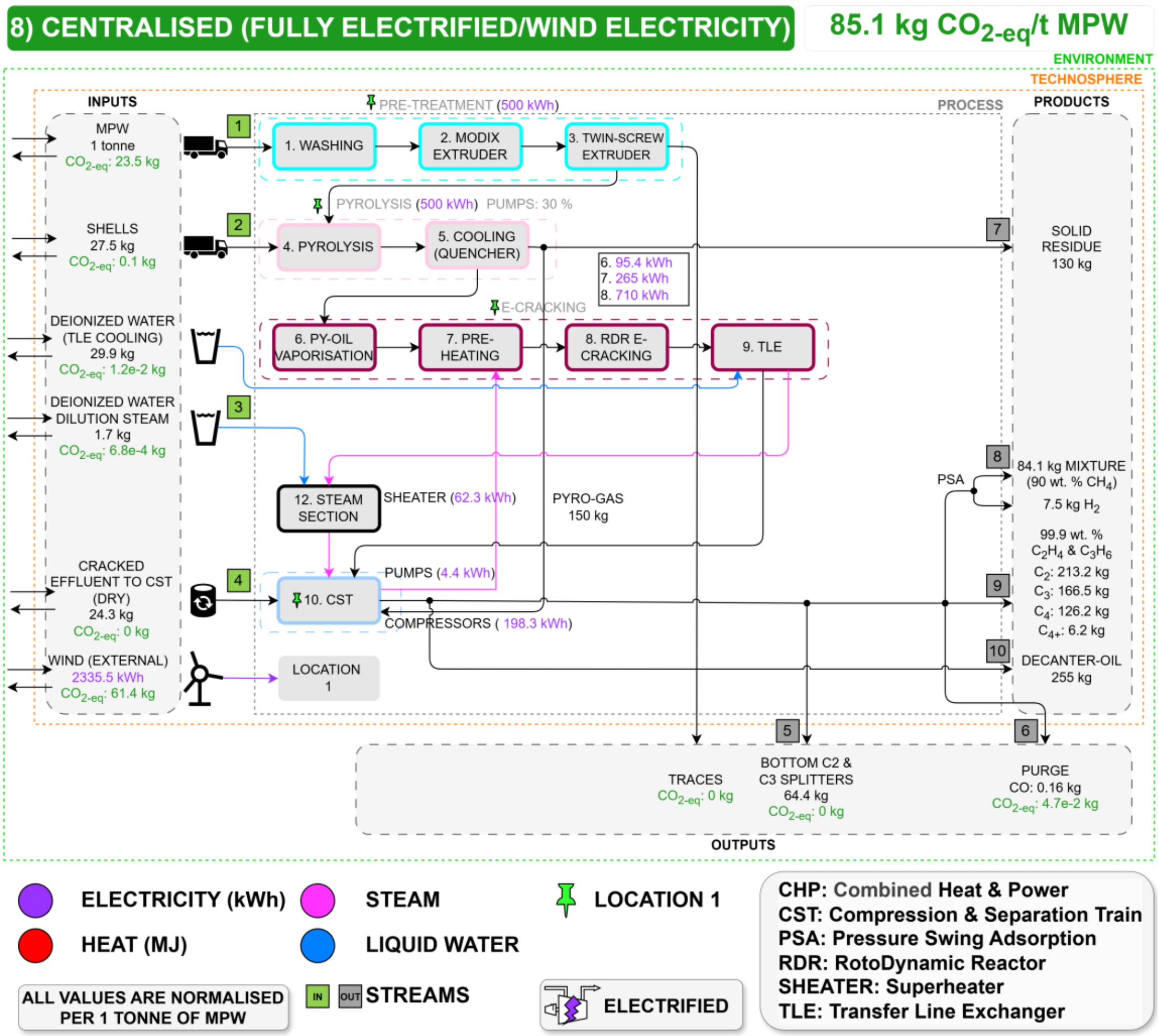

Figure S17: System boundaries and process-level energy, mass, and emission breakdown for the centralised fully electrified configuration supplied with wind electricity (Scenario 8). The figure follows the same structure, boundaries, and normalisation basis as Figure S1.

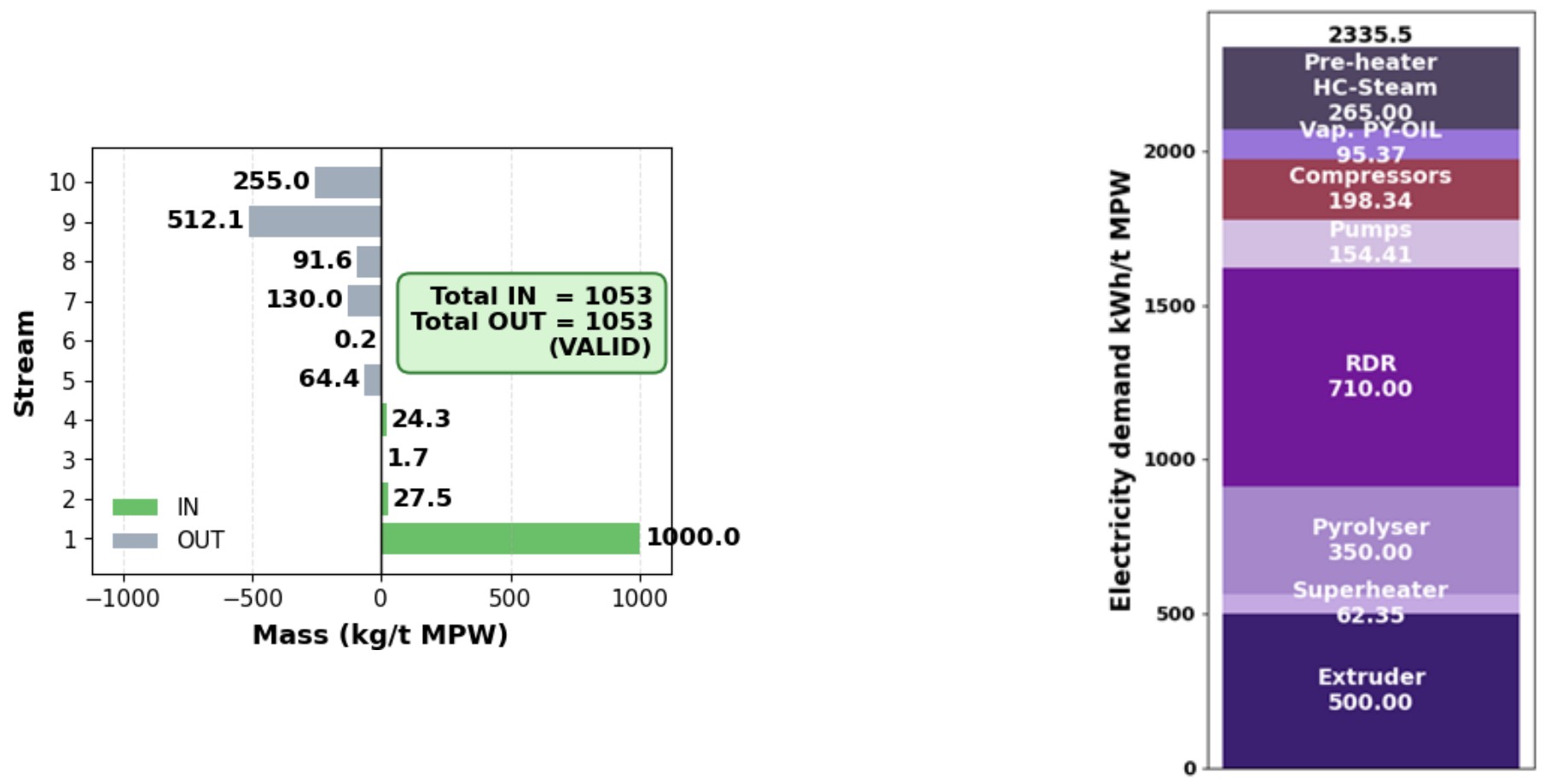

Figure S18: Mass and energy balances for the centralised fully electrified configurations (Scenarios 7–8). The left panel presents the overall mass balance distribution across process inputs and outputs, while the right panel shows the corresponding electricity demands of the main process units. In the fully electrified configuration, combustion-based heat generation is eliminated and all major process utilities are electrically driven

V. Technical Results/HEN for decentralised & centralised configurations

Overview of the proposed heat exchanger network (HEN) layouts for the decentralized and centralized process configurations.

Decentralised

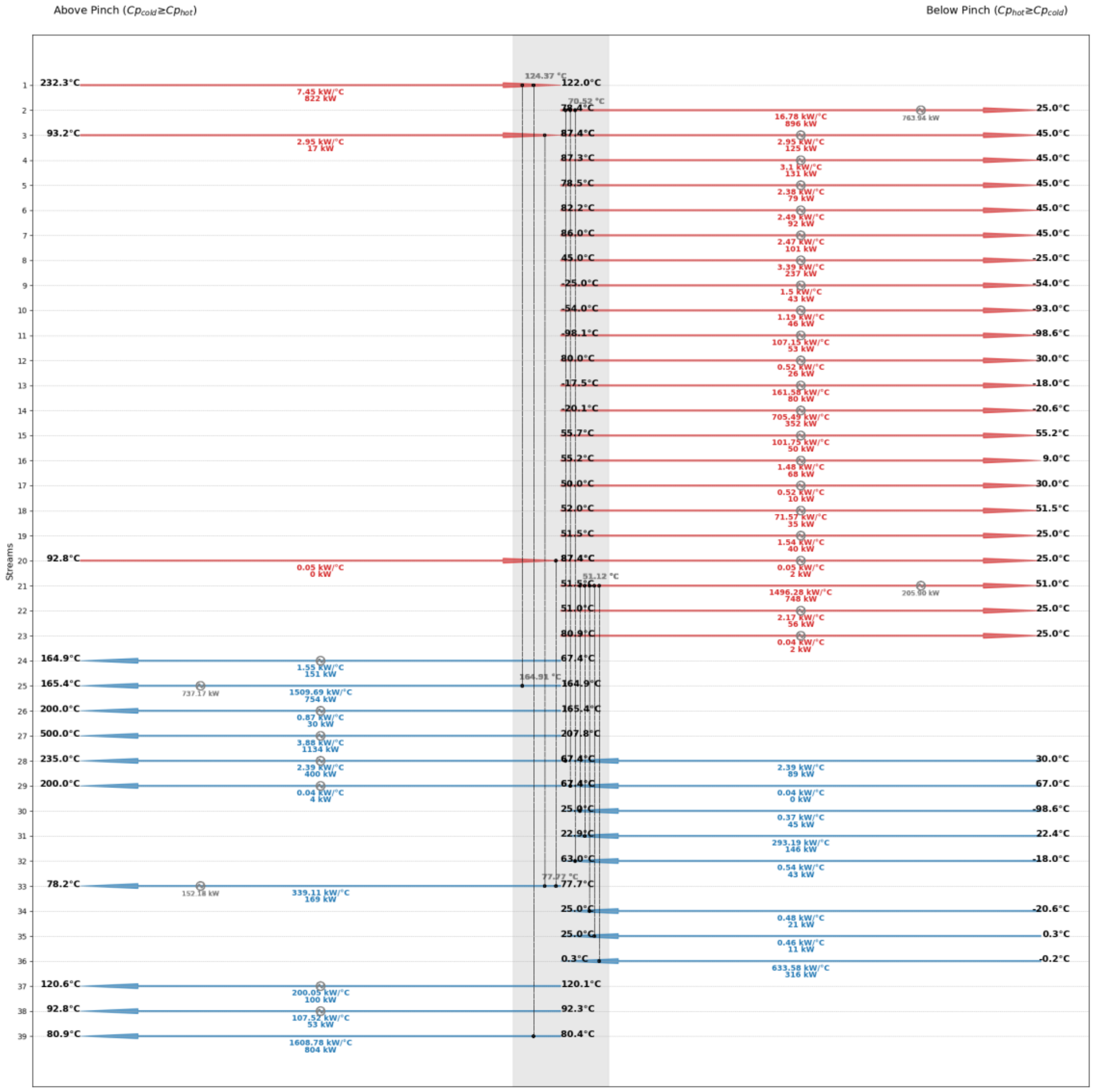

Centralised

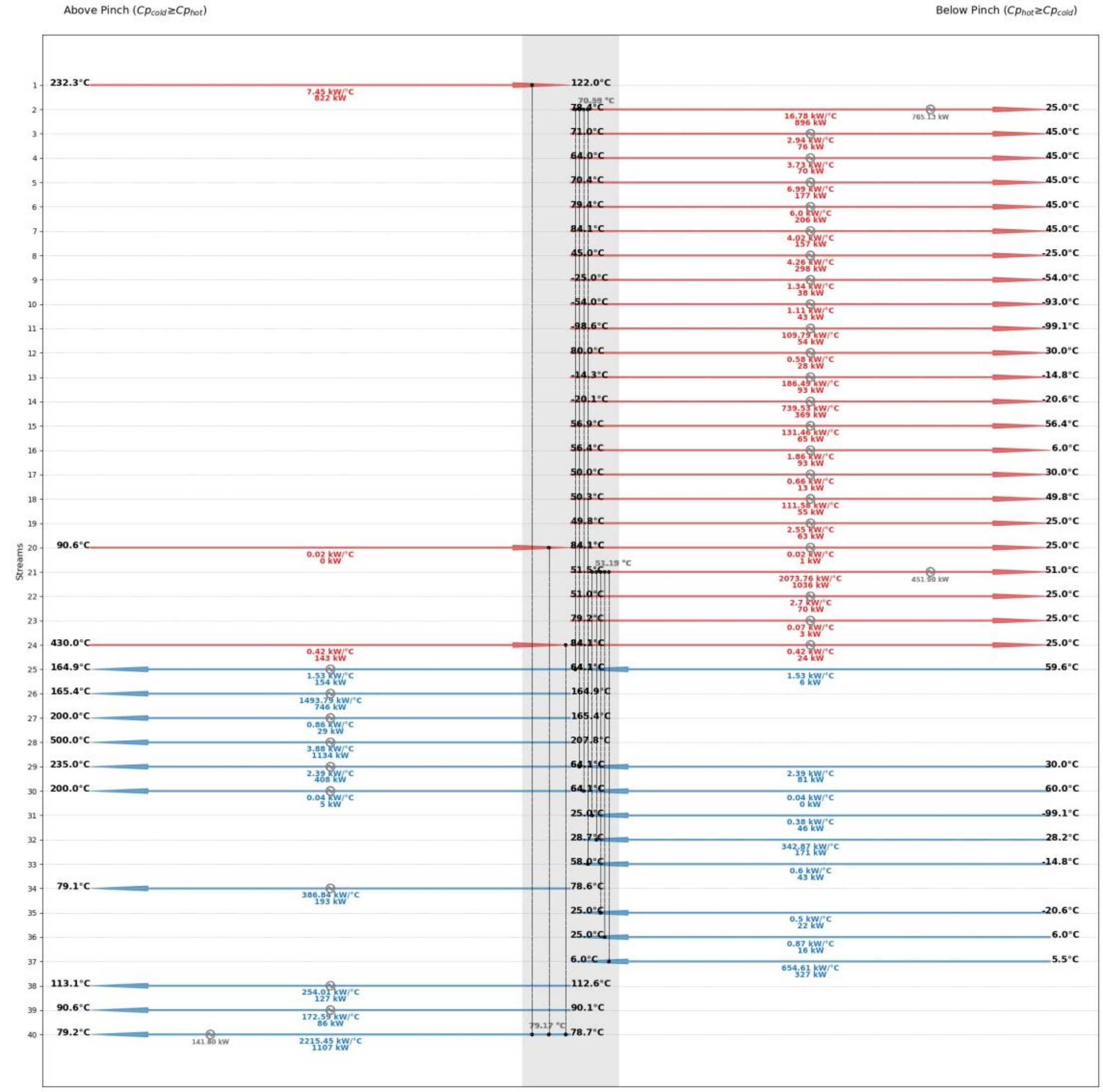

Figure S19: Proposed HEN for decentralised (top) vs. centralised (bottom) configuration. For unmatched streams, the heat exchanger is placed at the centre of the arrow. For matched streams, the heat exchanger is located at the left-hand end of the arrow above the pinch and at the right-hand end of the arrow below the pinch.

VI. Technical Results/Process Overview

Overview of the compression and separation train (CST) simulation framework, including the functions of the major process blocks, the hydrogenation reactions considered, and the normalized mass balances for the decentralized and centralized process configurations.

Table S6: Key functions of the major process blocks included in the CST simulation framework.

Block name	Code	Function

Oil Quench	Q-101	Cools the cracked gas using oil, reducing temperature after the RDR steam cracker.
Water Quench	Q-102	Further cools the gas, removing water-soluble compounds and water.
Compression Train	C-101 to C-105	Compresses the cracked gas, increasing pressure for downstream gas treatment and separation.
Decanter	DEC	Separates oil, water, and light-ends from the quenching and compression steps, improving the quality of streams.
Demethaniser	T-201	Separates methane and lighter components from ethylene, propylene, and other heavier hydrocarbons.
Deethaniser	T-202	Separates ethylene and ethane from heavier hydrocarbons (C3 and higher).
C2 Splitter	T-203	Separates ethylene (C_2H_4) from ethane (C_2H_6).
Depropaniser	T-204	Separates propane and propylene from butanes and heavier components.
Debutaniser	T-205	Separates butanes from heavier hydrocarbons like gasoline or heavier fractions.
C3 Splitter	T-206	Separates propylene (C_3H_6) from propane (C_3H_8).
Acetylene hydrogenation	R-201	Removes acetylene impurities from ethylene stream to meet purity specifications for polymer-grade ethylene (i.e., 99.9 wt.%).
Propyne hydrogenation	R-202	Removes propyne impurities from propylene stream to meet purity specifications for polymer-grade propylene (i.e., 99.9 wt.%).

Table S7: Acetylene and methylacetylene/propyne hydrogenation reactions.

aa	Reaction
1	$C_2H_2 + H_2 \longrightarrow C_2H_4$
2	$C_3H_4 + H_2 \longrightarrow C_3H_6$

Table S8: Mass balances (decentralised vs. centralised configuration). All values are normalised to t MPW^{-1}.

Description	Inputs	Outputs	Inputs	Outputs
	Decentralised		Centralised	
C2 Splitter (bottom stream)	0.0	27.4	0.0	41.5
C2 Splitter (top stream)	0.0	203.4	0.0	213.2
C3 Splitter (bottom stream)	0.0	13.0	0.0	22.9
C3 Splitter (top stream)	0.0	134.3	0.0	166.5
SOLID-RESIDUE	0.0	130.0	0.0	130.0
CO purge	0.0	0.2	0.0	0.2
CO_2 purge	0.0	0.0	0.0	0.0
Debutaniser (bottom)	0.0	15.6	0.0	6.2
Debutaniser (top)	0.0	79.7	0.0	126.2
Decanter oil (DEC-OIL)	0.0	210.2	0.0	255.0
Dilution steam	1.5	0.0	1.7	0.0
Hydrogen	0.0	7.9	0.0	7.5
Methane mixture	0.0	81.3	0.0	84.1
MPW	1000.0	0.0	1000.0	0.0
PYRO-GAS	0.0	150.0	0.0	0.0
Cracked effluent to CST (dry)	24.3	0.0	24.3	0.0

Shells	27.5	0.0	27.5	0.0
**Grand Total**	**1053**	**1053**	**1053**	**1053**

VII. Technical Results/Utilities Section

Both decentralised and centralised configurations employ the same utility framework, consisting of refrigeration cycles (propylene and ethylene), high pressure steam generation, and cooling-water networks. The distinction lies in the magnitude of the duties and the way energy supply is allocated. Here, non-normalised utility values are reported, as they more transparently reflect the absolute differences between the two configurations before any scaling to feed rates or functional units is performed (i.e., 4480 $kg \cdot h^{-1}$ of cracked effluent).

Refrigeration section

In both configurations, closed-loop propylene (purple) and ethylene (black) cycles provide sub-ambient cooling (< 20 °C), while cooling water covers above-ambient duties (>25 °C). The propylene loop operates between ~25 °C and −48.2 °C and is also used to condense ethylene, which then spans the lower range from −104.1 °C to −48.2 °C. All cooling duties are met with a minimum approach temperature difference of 4 °C ($\Delta T = 4$) between process streams and refrigerants. The decentralised CST requires ~425 kW of refrigeration compressor power, whereas the centralised configuration demands ~458 kW due to the additional vapor load from on-site PYRO-GAS processing. Ethylene refrigeration demand remains similar between the two cases, as the low-temperature targets do not change significantly (Figure S14).

Decentralised

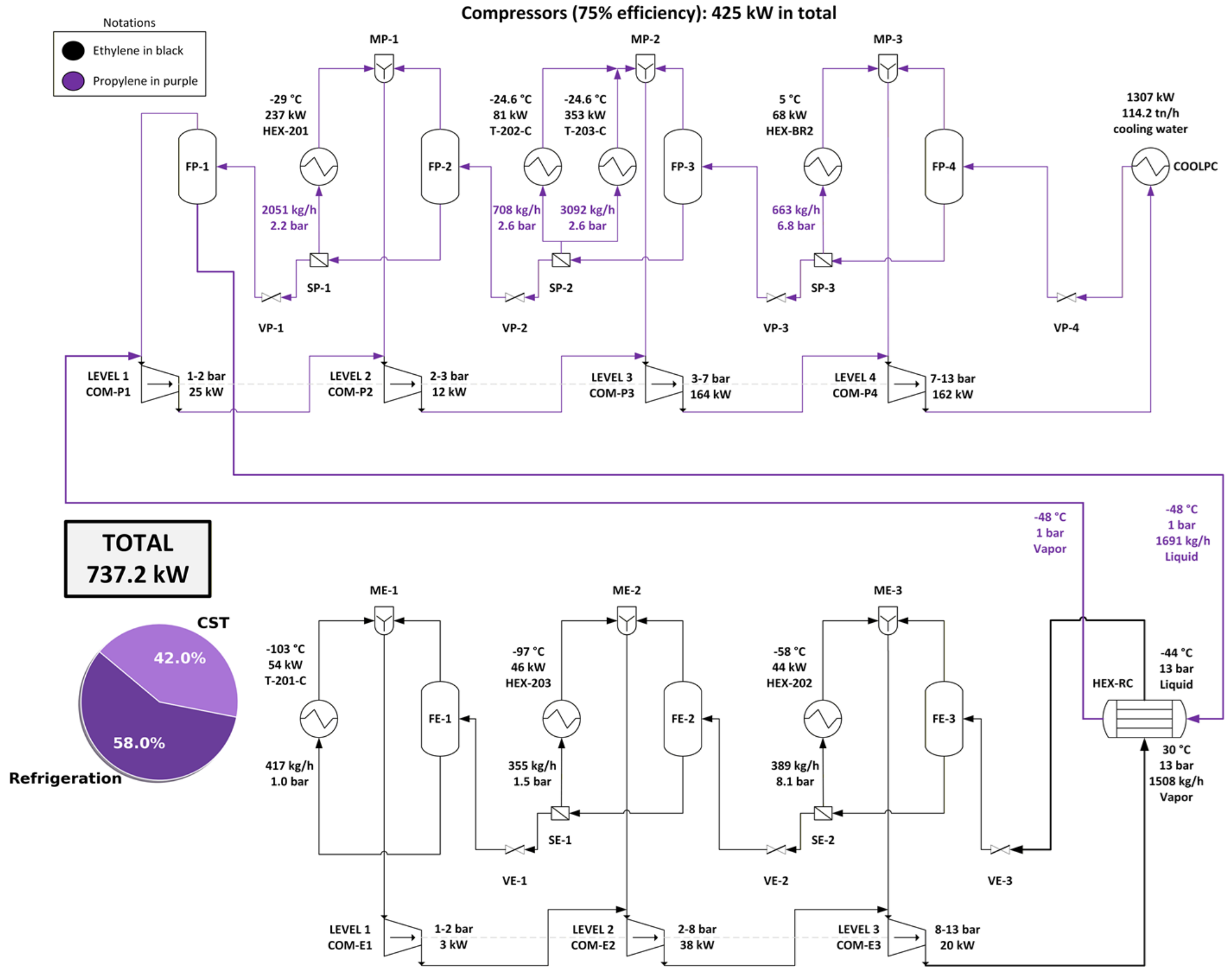

Centralised

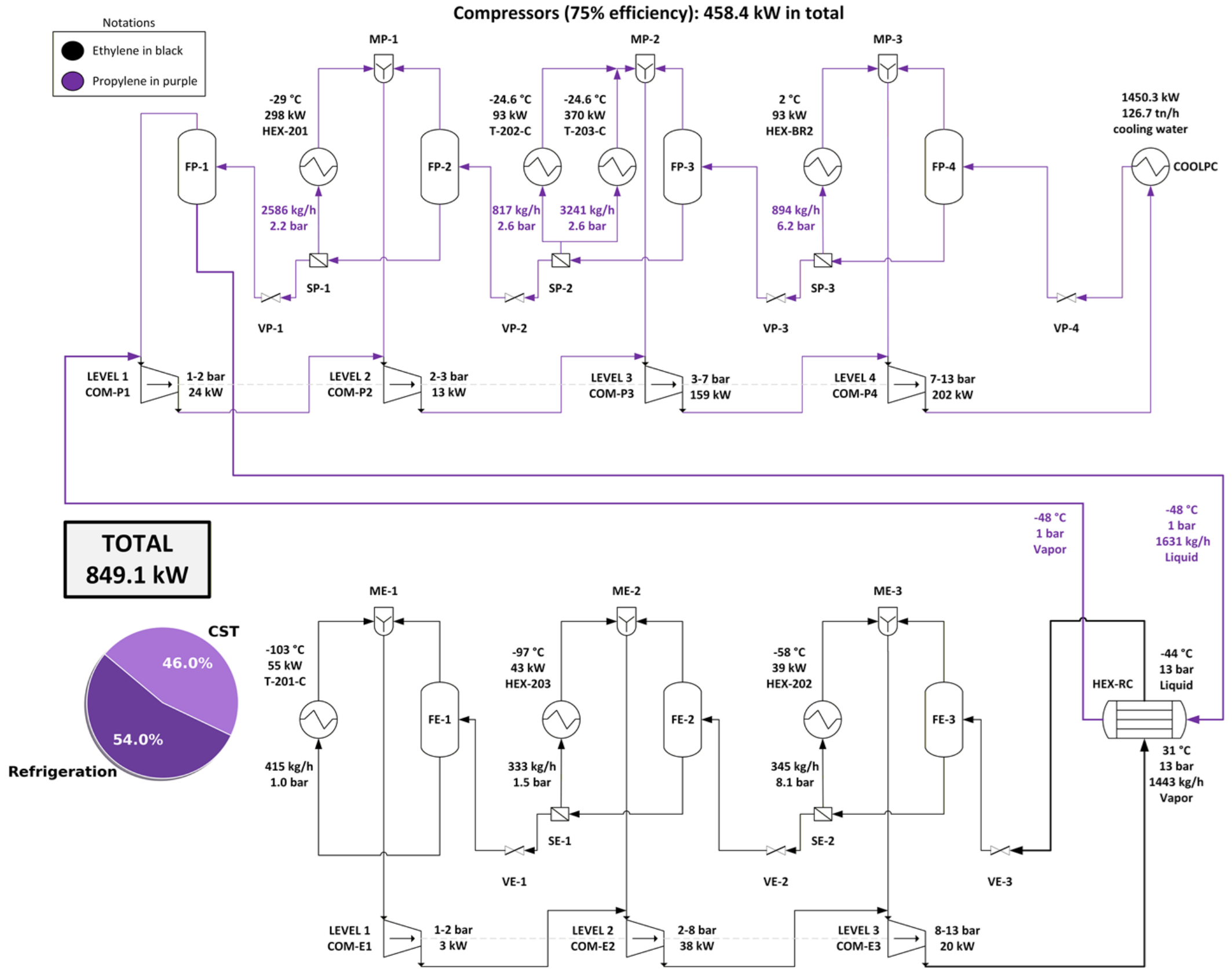

Figure S20: Graphical representation of the refrigeration section using ethylene and propylene (decentralised/top vs. centralised/bottom configuration).

Table S9: Cooling loads, target and refrigerant temperatures, pressure levels, and compressor power requirements of refrigeration cycles (decentralised vs. centralised configuration).

**Code**	**Power (kW)**	**Target T (°C)**	**Refrigerant**	**Mass Flow ($kg \cdot h^{-1}$)**	**T (°C), ΔT = 4**	**Level**	**Code**	**Pin (bar)**	**Pout (bar)**	**Duty (kW)**
**Decentralised**										
**Blocks**			**Refrigerants**			**Compressors**				
HEX-BR2	68.4	9.0	propylene	663	5	4	COM-P4	6.8	13.2	162.4
T-203-C	352.7	-20.59	propylene	3092	-24.6	3	COM-P3	2.6	6.8	164.5
T-202-C	80.8	-18.14	propylene	708	-24.6	3	COM-P3	2.6	6.8	
HEX-201	237.1	-25.0	propylene	2051	-29	2	COM-P2	2.2	2.6	11.6
**HEX-RC***	**204.7**	-48.2	propylene	1691		1	COM-P1	1	2.2	25.0
HEX-202	43.6	-54.0	ethylene	389	-58	3	COM-E3	8.1	13	20.3
HEX-203	46.2	-93.0	ethylene	355	-97	2	COM-E2	1.5	8.1	38.2
T-201-C	53.6	-98.63	ethylene	417	-102.63	1	COM-E1	1	1.5	2.8
**Total**	**882.6**	**HEX-RC*: excluded from total**				**Total**				**424.7**
**Centralised**										
**Blocks**			**Refrigerants**			**Compressors**				
HEX-BR2	93.5	9.0	propylene	894	2	4	COM-P4	6.2	13.2	202.3
T-203-C	369.8	-20.59	propylene	3241	-24.6	3	COM-P3	2.6	6.2	159.1
T-202-C	93.2	-18.14	propylene	817	-24.6	3	COM-P3	2.6	6.2	
HEX-201	298.5	-25.0	propylene	2586	-29	2	COM-P2	2.2	2.6	12.9
**HEX-RC***	**197.03**	-48.2	propylene	1631		1	COM-P1	1	2.2	24.0
HEX-202	38.7	-54.0	ethylene	345	-58	3	COM-E3	8.1	13	19.5
HEX-203	43.4	-93.0	ethylene	333	-97	2	COM-E2	1.5	8.1	37.6
T-201-C	54.9	-98.63	ethylene	415	-103.1	1	COM-E1	1	1.5	2.9
**Total**	**991.9**	**HEX-RC*: excluded from total**				**Total**				**458.4**

Table S10: Compressor power requirements of CST (decentralised vs. centralised configuration).

**Code**	**Duty (kW)**
**Decentralised**	
C-101	75.2
C-102	68.6
C-103	57.6
C-104	58.3
C-105	52.9
**Total**	**312.5**
**Decentralised**	
C-101	85.0
C-102	85.3
C-103	81.4

C-104	77.1
C-105	62.0
**Total**	**390.7**

Steam Section

The steam system operates differently depending on the degree of electrification. In CHP-powered configurations, steam provides both process heating and mechanical power for CST compressors via turbine expansion. In fully electrified configurations, compressors are electrically driven and turbines are removed; steam is then used exclusively for process heating, with pressure reduction achieved through expansion valves rather than mechanical expansion.

In the decentralised CHP-powered configuration (Figure S15-top), saturated steam produced in the TLE at 315 °C and 105 bar is superheated to 510 °C (712 kW) and expanded through a multistage turbine train (HP → MP → LP → condenser), supplying both process-heating and 737 kW of shaft power to the compressors. The corresponding steam demand is 3775 kg·h^{-1}.

In the decentralised fully electrified configuration (Figure S15-bottom), the turbines are removed and the steam produced in the TLE (~2485 kg·h^{-1}) is sufficient to cover all CST heating duties. This steam is only slightly superheated from 315 °C to 328 °C (62 kW duty) before undergoing a two-stage pressure reduction: first from 105 bar to 40 bar via SBV1 to generate MP steam for higher-temperature duties, and then from 40 bar to 11 bar (LP steam) via SBV2 to supply lower-temperature reboilers. As a result, the decentralised fully electrified case exhibits lower steam demand and negligible superheater duty compared with the CHP-powered configuration.

In the centralised CHP-powered configuration (Figure S16-top), higher vapor throughput increases steam demand to 4361 kg·h^{-1}. Of this, 2560 kg·h^{-1} is supplied by the TLE and 1801 kg·h^{-1} by an auxiliary boiler. As in the decentralised case, steam is superheated to 510 °C and expanded through a turbine train, providing 849 kW of compressor power while meeting all process heating requirements.

In the centralised fully electrified configuration (Figure S16-bottom), turbines are eliminated and compressors are electrically driven. Steam demand is 2560 kg·h^{-1}, all produced by the TLE. This steam is superheated (315 °C → 396 °C, 267 kW) before being expanded through the same two-stage pressure let-down system to produce medium-pressure (40 bar) and low-pressure (11 bar) steam for the corresponding CST heating duties.

Overall, fully electrified systems eliminate the turbine network and instead use staged pressure reduction to supply steam at the required conditions for each CST heating duty. With no mechanical power generation requirement, both total steam demand and superheater duty are reduced, especially in the decentralised configuration,

where TLE steam with minimal superheating is sufficient. Detailed operating conditions for all steam-using units are provided in Table S11.

Decentralised

CHP-powered

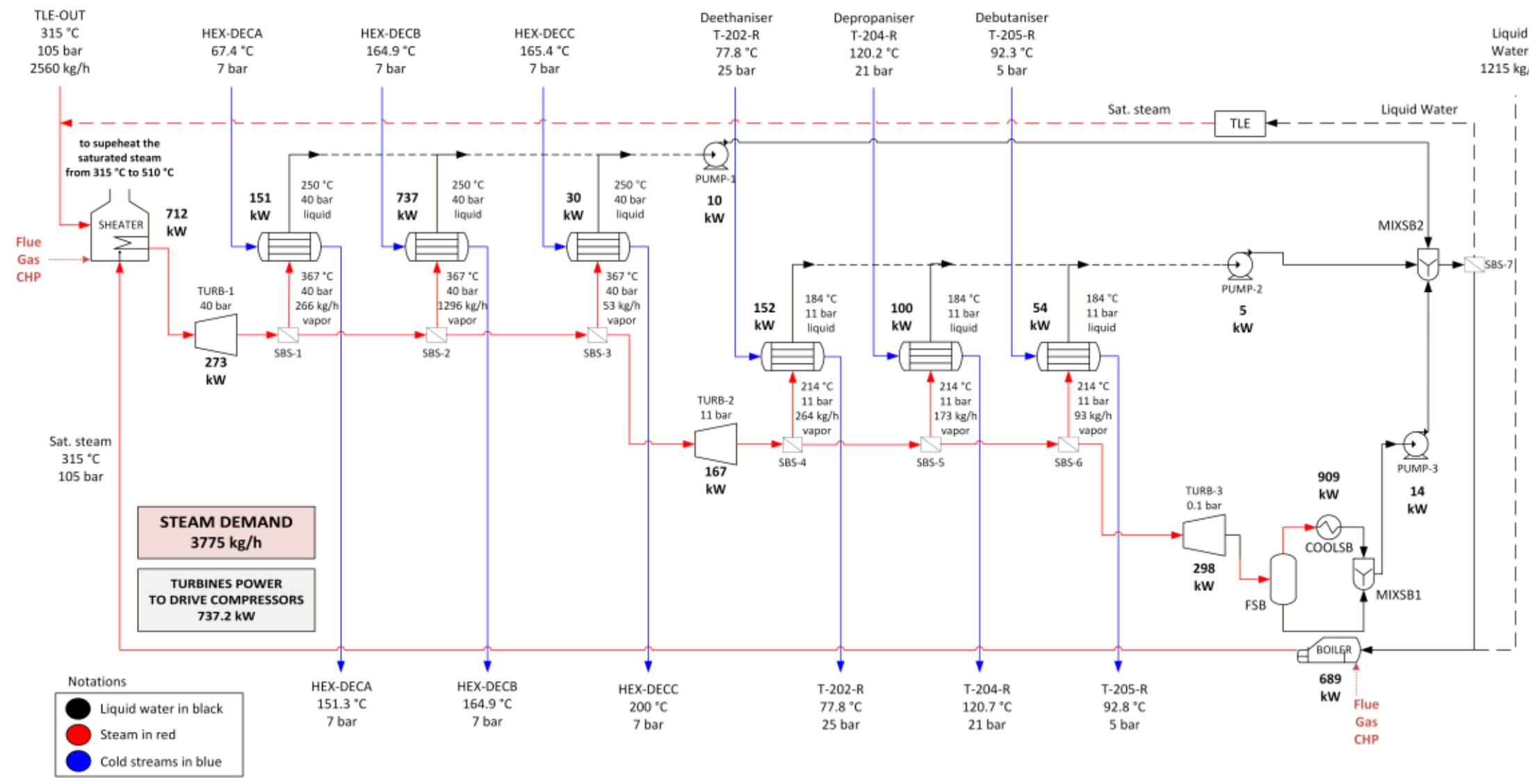

Fully electrified

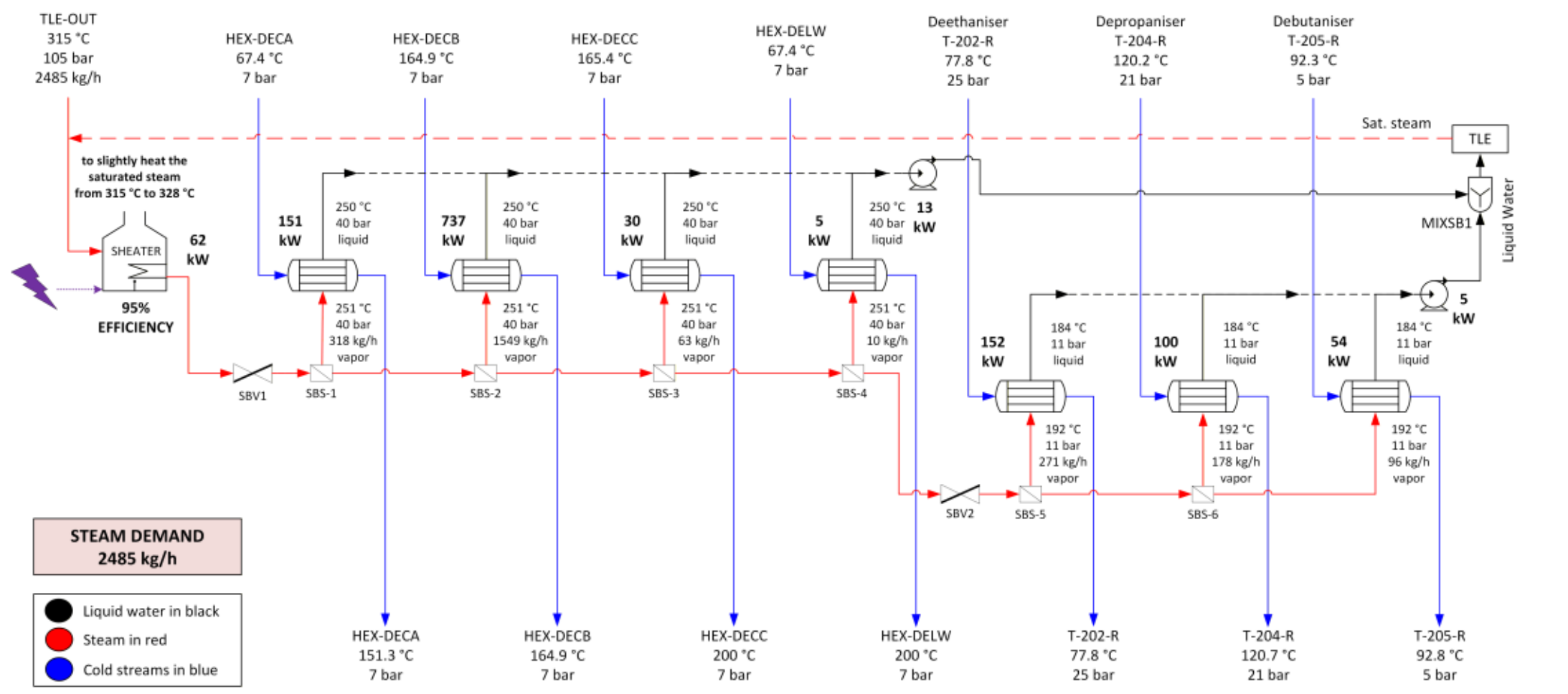

Figure S21: Graphical representation of the Steam Section: CHP-powered (top), fully electrified (bottom) (decentralised configuration).

Centralised

CHP-powered

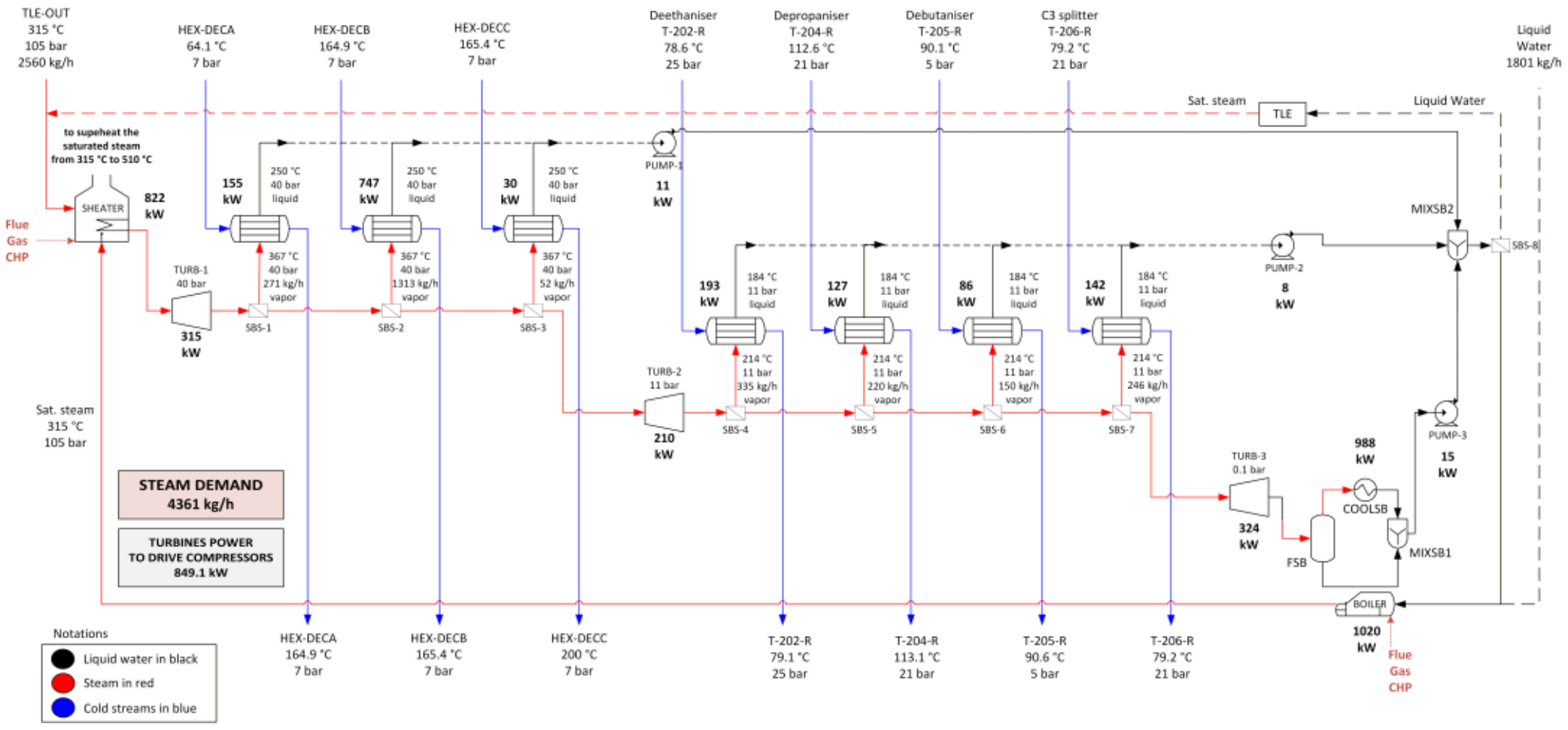

Fully electrified

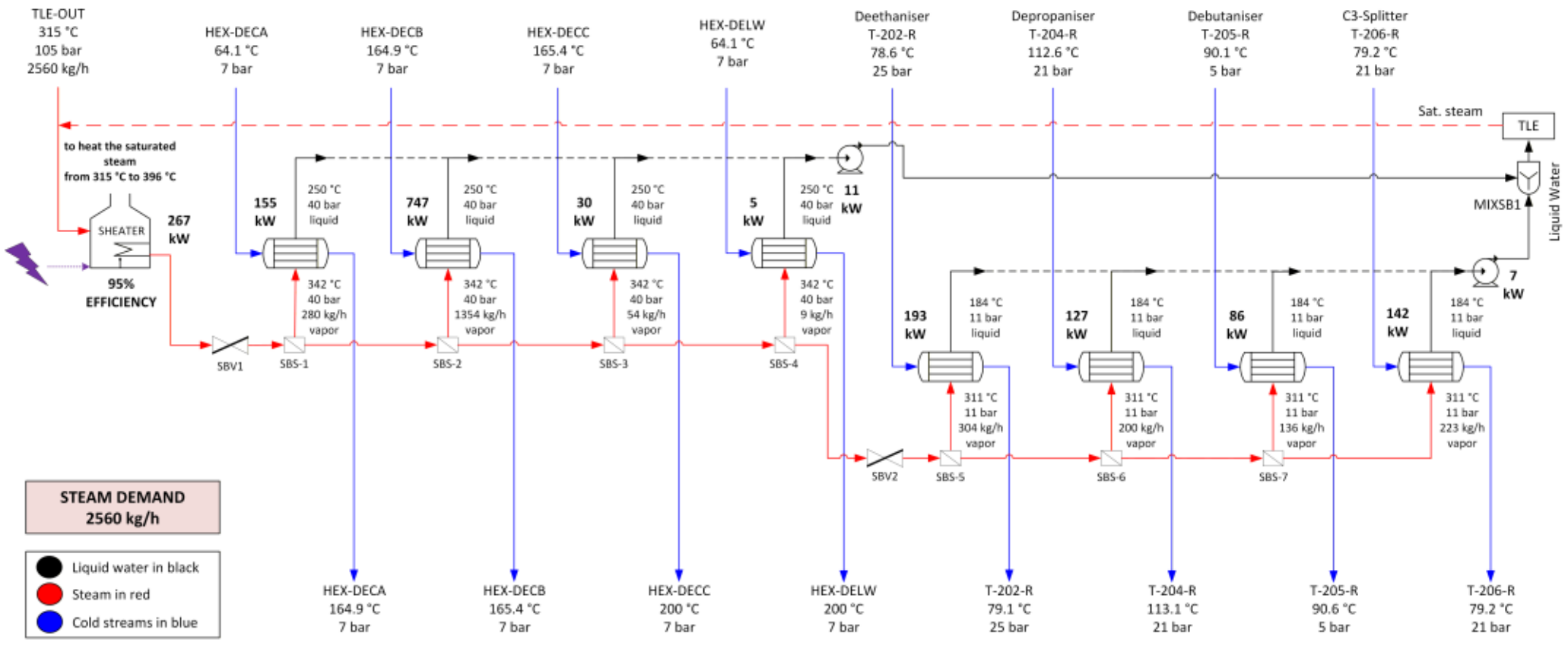

Figure S22: Graphical representation of the Steam Section: CHP-powered (top), fully electrified (bottom) (centralised configuration).

Table S11: Streams using steam as a hot utility (CHP-powered and fully electrified of decentralised vs. centralised configuration).

aa	Code	Tin (°C)	Tout (°C)	Heater (kW)	Mass Flow (kg·h⁻¹)	Tin (°C)	Pin (bar)	Tout (°C)	Unit
**Decentralised**									
**CHP-powered**									
**Blocks**					**Steam**				
24	HEX-DECA	67.4	164.9	151.3	266.1	366.8	40.0	250.4	CST
25	HEX-DECB	164.9	164.9	737.2	1296.4	366.8	40.0	250.4	CST
26	HEX-DECC	165.4	200.0	30.1	52.9	366.8	40.0	250.4	CST
30	T-202-R	77.8	77.8	152.2	263.9	214.3	11.0	184.1	CST
34	T-204-R	120.2	120.7	100.0	173.4	214.3	11.0	184.1	CST
35	T-205-R	92.3	92.8	53.8	93.3	214.3	11.0	184.1	CST
**Total CST**				**1224.6**	**2146.0**				
**Fully electrified**									
**Blocks**					**Steam**				

24	HEX-DECA	67.4	164.9	151.3	317.8	250.4	40.0	250.4	CST
25	HEX-DECB	164.9	164.9	737.2	1548.6	250.4	40.0	250.4	CST
26	HEX-DECC	165.4	200.0	30.1	63.2	250.4	40.0	250.4	CST
30	T-202-R	77.8	77.8	152.2	271.2	191.7	11.0	184.1	CST
34	T-204-R	120.2	120.7	100.0	178.2	191.7	11.0	184.1	CST
35	T-205-R	92.3	92.8	53.8	95.9	191.7	11.0	184.1	CST
**Total CST**				**1224.6**	**2459.9**				
29	HEX-DELW	67.4	200.0	4.7	9.9	250.4	40.0	250.4	HEX-DELW
**Total**				**1229.2**	**2484.9**				
**Centralised**									
**CHP-powered**									
**Blocks**					**Steam**				
25	HEX-DECA	64.1	164.9	154.6	271.9	366.8	40.0	250.4	CST
26	HEX-DECB	164.9	165.4	746.9	1313.4	366.8	40.0	250.4	CST
27	HEX-DECC	165.4	200.0	29.8	52.4	366.8	40.0	250.4	CST
31	T-202-R	78.6	79.1	193.4	335.4	214.3	11.0	184.1	CST
35	T-204-R	112.6	113.1	127.0	220.2	214.3	11.0	184.1	CST
36	T-205-R	90.1	90.6	86.3	149.7	214.3	11.0	184.1	CST
37	T-206-R	79.2	79.2	141.8	245.9	214.3	11.0	184.1	CST
**Total CST**				**1479.9**	**2588.8**				
**Fully electrified**									
**Blocks**					**Steam**				
25	HEX-DECA	64.1	164.9	154.6	280.4	341.8	40.0	250.4	CST
26	HEX-DECB	164.9	165.4	746.9	1354.5	341.8	40.0	250.4	CST
27	HEX-DECC	165.4	200.0	29.8	54.0	341.8	40.0	250.4	CST
31	T-202-R	78.6	79.1	193.4	303.8	311.0	11.0	184.1	CST
35	T-204-R	112.6	113.1	127.0	199.5	311.0	11.0	184.1	CST
36	T-205-R	90.1	90.6	86.3	135.6	311.0	11.0	184.1	CST
37	T-206-R	79.2	79.2	141.8	222.8	311.0	11.0	184.1	CST
**Total CST**				**1479.9**	**2550.5**				
30	HEX-DELW	64.1	200.0	5.2	9.2	341.8	40.0	250.4	HEX-DELW
**Total**				**1485.1**	**2559.8**				

Table S12: Main equipment involved in the Steam Section (CHP-powered and fully electrified of decentralised vs. centralised configuration).

**Block**	**Duty (kW)**	**Pin (bar)**	**Pout (bar)**	**Tin (°C)**	**Tout (°C)**	**Mass Flow (kg·h^{-1})**
**Decentralised**						
**CHP-powered**						
SUPERHEATER	711.8	105.0	105.0	314.6	510.0	3775.3

TURB-1	272.9	105.0	40.0	510.0	366.8	3775.3
TURB-2	166.6	40.0	11.0	366.8	214.3	2159.9
TURB-3	297.7	11.0	0.1	214.3	45.8	1629.3
PUMP-1	10.4	40.0	105.0	250.4	255.6	1615.4
PUMP-2	4.5	11.0	105.0	184.1	190.9	530.6
PUMP-3	13.7	0.1	105.0	45.8	53.0	1629.3
FSB	0.0	0.1	0.1	45.8	45.8	1629.3
COOLSB	908.6	0.1	0.1	45.8	45.8	1367.4
BOILER	688.5	105.0	105.0	314.6	510.0	3775.3
**Fully electrified**						
SUPERHEATER	61.8	105.0	105.0	314.6	328.0	2484.9
PUMP-1	12.5	40.0	105.0	250.4	255.1	1939.6
PUMP-2	4.6	11.0	105.0	184.1	190.9	545.3
VALVE-1	0.0	105.0	40.0	328.0	250.5	2484.9
VALVE-2	0.0	40.0	11.0	250.5	191.7	545.3
**Centralised**						
**CHP-powered**						
SUPERHEATER	822.3	105.0	105.0	314.6	510.0	4361.0
TURB-1	315.2	105.0	40.0	510.0	366.8	4361.0
TURB-2	210.0	40.0	11.0	366.8	214.3	2723.3
TURB-3	323.8	11.0	0.1	214.3	45.8	1772.2
PUMP-1	10.6	40.0	105.0	250.4	255.1	1637.7
PUMP-2	8.0	11.0	105.0	184.1	190.9	951.1
PUMP-3	14.9	0.1	105.0	45.8	53.0	1772.2
FSB	0.0	0.1	0.1	45.8	45.8	1772.2
COOLSB	988.2	0.1	0.1	45.8	45.8	1487.2
BOILER	1020.1	105.0	105.0	159.0	314.6	1801.0
**Fully electrified**						
SUPERHEATER	266.9	105.0	105.0	314.6	395.7	2560.0
PUMP-1	11.0	40.0	105.0	250.4	255.1	1698.1
PUMP-2	7.3	11.0	105.0	184.1	190.9	861.7
VALVE-1	0.0	105.0	40.0	395.7	341.8	2560.0
VALVE-2	0.0	40.0	11.0	341.8	311.0	861.7

Cooling Water Section

Cooling water networks follow the same closed-loop design in both configurations, operating with a 10 °C approach (20 °C in, 30 °C out). The decentralised CST requires 3941 kW of cooling, compared to 4670 kW in the centralised case. This difference reflects the additional duty of condensing and cooling the full PYRO-GAS stream in centralised processing (Figure S17).

In the decentralised case, the CST accounts for 1725 kW of cooling demand (Table S13), while the refrigeration and steam sections contribute 1307 kW (COOLPC) and 908 kW (COOLSB), respectively, for a total of 3941 kW. In the centralised case, the CST requires

2331 kW, with additional contributions of 1450 kW from the refrigeration section and 988 kW from the steam section, for a total of 4670 kW (Table S13).

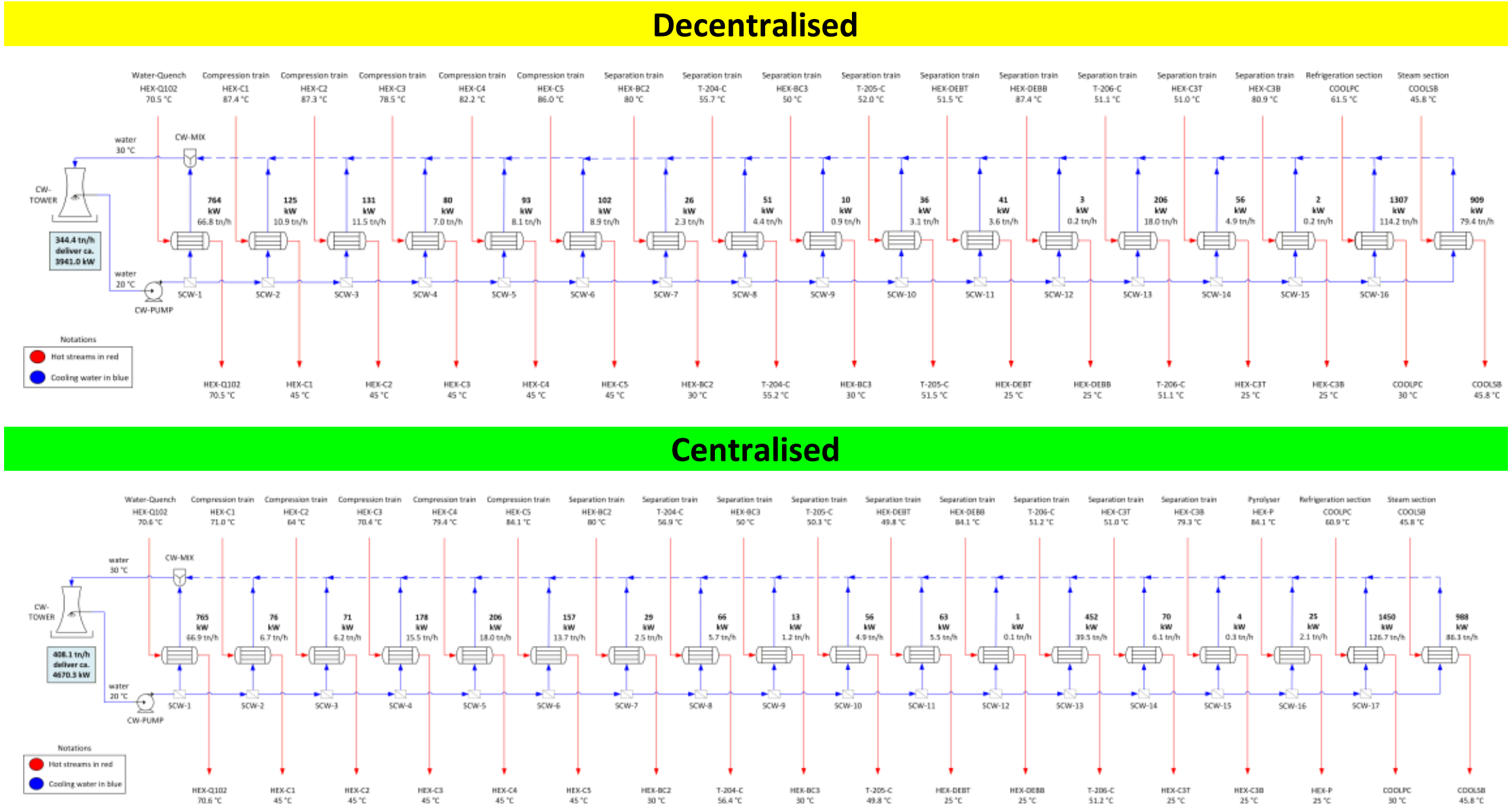

Figure S23: Graphical representation of the Cooling Water Section (decentralised vs. centralised configuration).

The sum of cooling duties from the refrigeration and cooling-water section matches the cold-utility requirements obtained after heat integration (2607.8 kW in the decentralised configuration and 3223.8 kW in the centralised configuration), affirming both the internal heat recovery strategy and the accuracy of the utility model.

Table S13: Streams using cooling water as a cold utility (decentralised vs. centralised configuration).

**Code**	**Section**	**Duty (kW)**	**Tin (°C)**	**Tout (°C)**	**Tin (°C)**	**Tout (°C)**	**Mass Flow ($t \cdot h^{-1}$)**
**Decentralised**							
**Blocks**					**Cooling water**		
HEX-Q102	CST	763.9	70.5	70.5	30.0	20.0	66.8
HEX-C1	CST	125.3	87.4	45.0			10.9
HEX-C2	CST	131.1	87.3	45.0			11.5
HEX-C3	CST	79.7	78.5	45.0			7.0
HEX-C4	CST	92.6	82.2	45.0			8.1
HEX-C5	CST	101.5	86.0	45.0			8.9
HEX-BC2	CST	26.1	80.0	30.0			2.3
T-204-C	CST	50.9	55.7	55.2			4.4
HEX-BC3	CST	10.3	50.0	30.0			0.9
T-205-C	CST	35.8	52.0	51.5			3.1
HEX-DEBT	CST	40.7	51.5	25.0			3.6
HEX-DEBB	CST	2.8	87.4	25.0			0.2
T-206-C	CST	205.9	51.1	51.1			18.0
HEX-C3T	CST	56.5	51.0	25.0			4.9

HEX-C3B	CST	2.1	80.9	25.0			0.2
**Total**		**1725.3**			**Total**		**150.8**
COOLPC	Refrigeration	1307.2	61.5	30.0	30.0	20.0	114.2
COOLSB	Steam	908.6	45.8	45.8			79.4
**Total**		**3941.0**			**Total**		**344.4**
**Centralised**							
**Blocks**					**Cooling water**		
HEX-Q102	CST	765.1	70.6	70.6	30.0	20.0	66.9
HEX-C1	CST	76.4	71.0	45.0			6.7
HEX-C2	CST	70.8	64.0	45.0			6.2
HEX-C3	CST	177.6	70.4	45.0			15.5
HEX-C4	CST	206.2	79.4	45.0			18.0
HEX-C5	CST	157.2	84.1	45.0			13.7
HEX-BC2	CST	29.0	80.0	30.0			2.5
T-204-C	CST	65.7	56.9	56.4			5.7
HEX-BC3	CST	13.3	50.0	30.0			1.2
T-205-C	CST	55.8	50.3	49.8			4.9
HEX-DEBT	CST	63.3	49.8	25.0			5.5
HEX-DEBB	CST	1.1	84.1	25.0			0.1
T-206-C	CST	451.9	51.2	51.2			39.5
HEX-C3T	CST	70.0	51.0	25.0			6.1
HEX-C3B	CST	3.9	79.3	25.0			0.3
HEX-P	Pyrolyser	24.5	84.1	25.0			2.1
**Total**		**2231.8**			**Total**		**195.0**
COOLPC	Refrigeration	1450.3	60.9	30.0	30.0	20.0	126.7
COOLSB	Steam	988.2	45.8	45.8			86.3
**Total**		**4670.3**			**Total**		**408.1**

VIII. Life-Cycle GHG Emissions

Breakdown of greenhouse gas (GHG) emissions across the eight evaluated scenarios, illustrating the contribution of individual process sections and the influence of process configuration, electrification level, and electricity source.

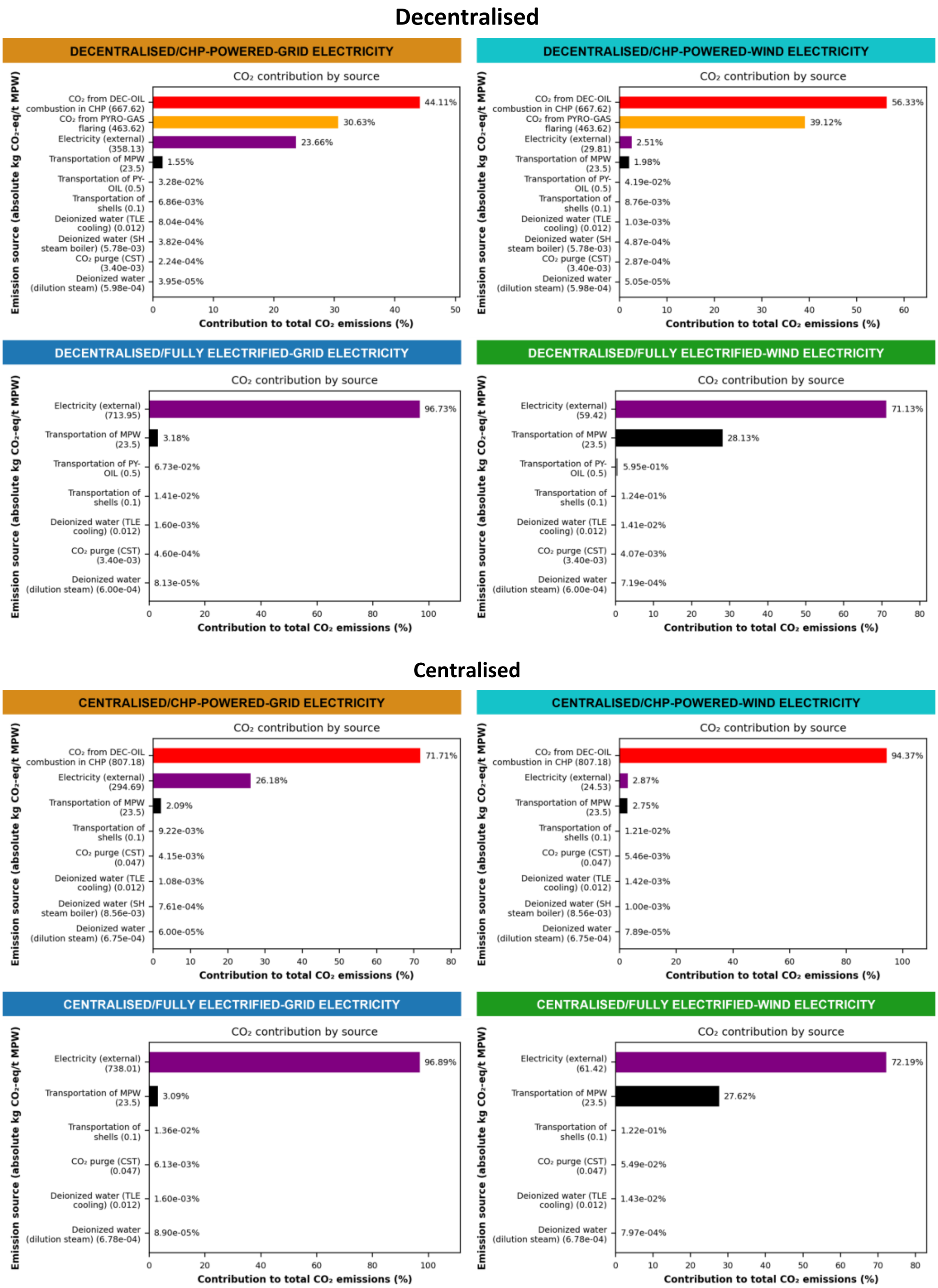

Figure S24: Complete breakdown of GHG emissions of process across eight scenarios: impact of configuration, electrification level, and electricity source (decentralised configurations at the top, centralised configurations at the bottom).

IX. Ecoinvent Database

Overview of the background life-cycle inventory (LCI) datasets used for material and energy supply in the environmental assessment.

Table S14: Background data for the supply of materials and energy retrieved from the ecoinvent v3.9 and v3.10 databases. The "Dataset" column reports the corresponding Ecoinvent dataset identifiers, which can be used in EcoQuery (e.g., https://ecoquery.ecoinvent.org/3.10/cutoff/dataset/20377) to access the corresponding dataset entries.[8]

Utilities/Processes	LCI Process Name	Geographic Region	Amount of Referent Product	kg $CO_{2\text{-eq}}$ (GWP100)	Comments	Dataset:
**Transportation of MPW**	market for transport, freight, lorry 7.5-16 metric ton, EURO5	RER	t*km (this is delivering the transportation service of 1 metric ton across the distance of 1 km)	0.235	**Included activities start**:<br>This activity starts with the service generation.<br>**Included activities end:**<br>This activity ends with the service supplied to its consumers. Transport or losses are considered irrelevant to this product.<br>**Details**: The vehicle operates with diesel; its emission standard is classified as EURO5. The average freight load factor of a 7.5-16 metric ton lorry is 3.29 tonnes, with a gross vehicle weight (GVW) of 9.29 tonnes. It has a lifetime capacity of 540,000 km. Data for transport is calculated for an average load factor, including empty return trips.	18657
**Transportation of PY-OIL**	market for transport, freight, sea, tanker for petroleum, heavy fuel oil	RER	t*km (this is delivering the transportation service of 1 metric ton across the distance of 1 km)	0.00665	**Included activities start**:<br>This activity starts with the service generation.<br>**Included activities end:**<br>This activity ends with the service supplied to its consumers. Transport or losses are considered irrelevant to this product.<br>**Details**: This service only considers the transportation of liquid goods by a tanker for petroleum. The ship operates with heavy fuel oil and has a mass of 7,774,400 kg. The DWT (load capacity) of the container ship is 36,000 tonnes, and it is estimated to transport an average of 4,100 million tonne.kms per year for 25 years. The transport node indirectly considers empty trips (if any) by considering the total amount of freight transported throughout the lifetime of the mobile infrastructure.	20377
**Transportation of shells**	transport, freight, lorry, all sizes, EURO6 to generic market for transport, freight, lorry, unspecified	RER	t*km (this is delivering the transportation service of 1 metric ton across the distance of 1 km)	0.153	This is a linking activity.<br>**Included activities start**:<br>This activity starts with the service generation.<br>**Included activities end:**<br>This activity ends with the service supplied to its consumers. Transport or losses are considered irrelevant to this product.<br>**Details**: The vehicle operates with diesel, and it provides a fleet average that includes different lorry classes as well as EURO classes. This service combines data for transport, which is calculated for an average load factor, including empty return trips	18337
**Deionized water for TLE cooling or dilution steam or**	market for water, deionised	Europe, without Switzerland	1 kg deionized water	0.000407	**Included activities start**:<br>This activity starts at the gate of the activities that produce water, deionized, within the geography of Europe without Switzerland.<br>**Included activities end:**	15160

**superheated steam production**					This activity ends with the supply of water, deionized, to the consumers of this product. Transport or losses are considered irrelevant to this product.<br>**Details**: Deionized water is free of most mineral ions (such as iron, sodium, calcium, copper, sulphate, chloride among others). It is used for the manufacture of products in the following sectors: cooling systems, machines lubrication, cosmetic and pharmaceutical, medicine production and food processing	
**Grid electricity**	market group for electricity, high voltage	RER	1kWh	0.316	Represents all markets contained in the geography of the dataset.<br>**Included activities start**:<br>This activity starts at the gate of markets that deliver 'electricity, high voltage', in geographies contained within the geography of this dataset.<br>**Included activities end**:<br>This activity ends with the supply of 'electricity, high voltage', to the consumers of this product within the geography of Europe.<br>**Details**: This product represents electrical energy measured in kWh. If electricity is taken from a market for electricity, the transmission infrastructure, country-specific losses and transformation losses (for markets for medium and low voltage) are included. Covers voltages above 24 kV.	13694
**Renewable electricity (wind harvested)**	electricity production, wind, >3MW turbine, onshore	Netherlands	1kWh	0.0263	Represents the production of high voltage electricity at onshore grid-connected wind power plants with a capacity of more than 3MW (3MW excluded) in Netherlands.<br>**Included activities start:**<br>From operation of the wind power plant to high voltage electricity at the plant. According to (European Communities 2001), electricity produced with wind turbines in a wind power plant is connected to medium voltage or high voltage systems, depending on the size of the windfarm. Normally, each wind turbine contains a transformer from low voltage to medium voltage. It is assumed here that there are no stand-alone grid-connected small wind turbines and that all electricity is connected to the network in the form of high voltage electricity.<br>**Included activities end:**<br>This activity ends with the production of 1kWh of high voltage electricity produced at the power plant and delivered to the grid. This dataset includes - the operation and maintenance of the wind power plant - change of lubricating oil every year. This dataset doesn’t include - raw materials extraction, decommissioning and waste treatment as these activities are already included in infrastructure datasets - transformation of the electricity produced in order to deliver into the grid as this is already included in the infrastructure datasets.	6345
**Incineration**	treatment of waste plastic, mixture, municipal incineration	GLO	1 kg plastic waste, mixture	2.38	**Included activities start:**<br>The activity starts with the waste being available at the gate of the treatment facility. Collection and transportation of waste to the treatment plant is not included.<br>**Included activities end:**<br>The activity ends with the waste being treated and waste-specific emissions from the incineration process and auxiliary material consumption for flue gas cleaning are released to the environment (air and water). Infrastructure and waste-specific energy demands for Municipal Solid Waste Incineration (MSWI) and net energy generation are included.<br>**Details**: The waste is treated in the incineration plant with flue gas cleaning (controlled combustion). Auxiliary material consumption for this step of air pollution control (APC) is included in the process. Air pollution control includes processstatic precipitator for fly ash (ESP), wet flue gas scrubber and 25% SNCR, 42.77% SCR-high dust, 32.68% SCR-low dust-DeNOx facilities and 0% without DeNOx (weighted according to mass of burnt waste). Efficiency of iron scrap separation from slag: 58%. Efficiency of	29914

					aluminum scrap separation from slag: 31%. Efficiency of copper scrap separation from slag: 31%. Gross electric efficiency technology mix 15.84% and Gross thermal efficiency technology mix 28.51%. Net energy production: 3.93MJ·kg^{-1} electric energy and 7.67MJ·kg^{-1} thermal energy.	
**Open burning**	treatment of waste plastic, mixture, open burning	The modeling parameters represent a generic open burning process, not geographically specific.	1 kg plastic waste, mixture	2.44	Represents the activity of waste disposal of 'waste plastic, mixture' in uncontrolled, open burning, without flue gas treatment. Residues (minus any actually recycled metals) are left on site. Plastic waste mixture is primarily generated in a wide range of production activities in many different sectors, like building infrastructure, production of plastics, chemicals, aircraft, ferries, heavy machinery, etc. **Included activities starts**: The activity starts with the waste being available at the location (gate) of the uncontrolled disposal. Collection and transportation of waste to the uncontrolled disposal location is not included. **Included activities ends**: The activity ends with the waste being disposed of in an uncontrolled manner and waste-specific emissions from open burning are released to the environment (air and soil).	18054
**Landfill**	treatment of waste plastic, mixture, sanitary landfill	RoW	1 kg plastic waste, mixture	0.0933	**Included activities start**: The activity starts with the waste being available at the gate of the treatment facility. Collection and transportation of waste to the treatment plant is not included. **Included activities end**: The activity ends with the waste being treated and waste-specific emissions from the landfill is released to the environment (air due to landfill gas, water due to landfill leachate, and subsequently into groundwater). **Details**: This is an inventory for a sanitary (controlled) landfill for municipal solid waste. Ultimate average landfill height is 20 meters. Operation phase duration is 30 years. The total raw landfill gas mass produced in the landfill from this waste is 0.007913 kg. The total CH_4 mass in the raw landfill gas is 0.006151 kg. The percentage of landfill gas directly emitted is 47 wt.%. The percentage of landfill gas captured and flared is 18.02w%. The percentage of landfill gas captured and utilised is 34.98%.	11079

References

(1) Wu, J.; Prausnitz, J. M. Phase Equilibria for Systems Containing Hydrocarbons, Water, and Salt: An Extended Peng−Robinson Equation of State. *Ind. Eng. Chem. Res.* **1998**, *37* (5), 1634–1643. https://doi.org/10.1021/IE9706370.

(2) Wagner, W.; Pruß, A. The IAPWS Formulation 1995 for the Thermodynamic Properties of Ordinary Water Substance for General and Scientific Use. *J. Phys. Chem. Ref. Data* **2002**, *31* (2), 387–535. https://doi.org/10.1063/1.1461829.

(3) Eleftheriou G. Kastrinaki. *Engineering Physical Properties*, 3rd ed.; Tziola: Thessaloniki, 2004.

(4) Steffen, V.; Antonio, E. Steady - State Modeling of Equilibrium Distillation. In *Distillation - Innovative Applications and Modeling*; IntechOpen, 2016; pp 3–30. https://doi.org/10.5772/66833.

(5) Xiao, J.; Peng, Y.; Bénard, P.; Chahine, R. Thermal Effects on Breakthrough Curves of Pressure Swing Adsorption for Hydrogen Purification. *Int. J. Hydrogen Energy* **2016**, *41* (19), 8236–8245. https://doi.org/10.1016/j.ijhydene.2015.11.126.

(6) Zhu, X.; Shi, Y.; Li, S.; Cai, N. Elevated Temperature Pressure Swing Adsorption Process for Reactive Separation of CO/CO2 in H2-Rich Gas. *Int. J. Hydrogen Energy* **2018**, *43* (29), 13305–13317. https://doi.org/10.1016/j.ijhydene.2018.05.030.

(7) Kheimi, M.; Salamah, S. K. Simulation of Temperature Swing Adsorption Process to Purify Hydrogen for Fuel Cell Uses by SAPO34 as Adsorbent. *Chemosphere*. 2023. https://doi.org/10.1016/j.chemosphere.2023.139454.

(8) ecoinvent. *ecoinvent*. https://ecoinvent.org/. (accessed 2026-07-13).
